# Transcriptomic analysis of marine endophytic fungi extract identifies highly enriched anti-fungal fractions targeting cancer pathways in HepG2 cell lines

**DOI:** 10.1186/s12864-020-6684-z

**Published:** 2020-03-30

**Authors:** Ethel Juliet Blessie, Wasco Wruck, Benaiah Annertey Abbey, Audrey Ncube, Nina Graffmann, Vincent Amarh, Patrick Kobina Arthur, James Adjaye

**Affiliations:** 1 West African Center for Cell Biology of Infectious Pathogens, Department of Biochemistry, Cell and Molecular Biology, University of Ghana, Accra, Ghana; 2 Institute for Stem Cell Research and Regenerative Medicine, Medical faculty, Heinrich-Heine University, Düsseldorf, Germany

**Keywords:** Marine endophytic fungi, Seaweed, Anti-cancer extract, Anti-fungal resistance, HepG2, Proliferation, Cancer pathways, Protein interaction network

## Abstract

**Background:**

Marine endophytic fungi (MEF) are good sources of structurally unique and biologically active secondary metabolites. Due to the increase in antimicrobial resistance, the secondary metabolites from MEF ought to be fully explored to identify candidates which could serve as lead compounds for novel drug development. These secondary metabolites might also be useful for development of new cancer drugs. In this study, ethyl acetate extracts from marine endophytic fungal cultures were tested for their antifungal activity and anticancer properties against *C. albicans* and the human liver cancer cell line HepG2, respectively. The highly enriched fractions were also analyzed by high performance liquid chromatography coupled with high resolution mass spectrometry (HPLC-HRMS) and their effect on the HepG2 cells was assessed via transcriptomics and with a proliferation assay.

**Results:**

We demonstrated that the fractions could reduce proliferation in HepG2 cells. The detailed transcriptome analysis revealed regulation of several cancer- and metabolism-related pathways and gene ontologies. The down-regulated pathways included, cell cycle, p53 signaling, DNA replication, sphingolipid metabolism and drug metabolism by cytochrome P450. The upregulated pathways included HIF-1 signaling, focal adhesion, necroptosis and transcriptional mis-regulation of cancer. Furthermore, a protein interaction network was constructed based on the 26 proteins distinguishing the three treatment conditions from the untreated cells. This network was composed of central functional components associated with metabolism and cancer such as TNF, MAPK, TRIM21 and one component contained APP.

**Conclusions:**

The purified fractions from MEF investigated in this study showed antifungal activity against *C. albicans* and *S. cerevisiae* alone or both and reduced proliferation of the human liver cancer cell line HepG2 implicating regulation of several cancer- and metabolism-related pathways. The data from this study could be instrumental in identifying new pathways associated with liver cancer anti-proliferative processes which can be used for the development of novel antifungal and anti-cancer drugs.

## Background

The majority of neglected tropical diseases are caused by fungal pathogens, as well as helminths and protozoans. Efforts towards drug discovery that utilizes fungal cells as the target organism has the potential of identifying compounds that exclusively target fungal pathogens and compounds that share cellular targets with other non-pathogenic fungal eukaryotes. Fungi residing in terrestrial and marine environments are being explored for the development of novel antifungal compounds due to their potential to produce biologically active metabolites [1]. Metabolites from terrestrial and marine fungi are interesting because they have been proven to be a good source of structurally unique and biologically active secondary metabolites which can serve as starting materials for development of antifungal drugs [1]. The relevance of antifungal drugs in clinics is threated by the continual emergence of drug resistant fungal pathogens, thereby complicating patient management [2]. Although new antifungal agents have been introduced to combat this problem, the development of resistance to anti-fungal drugs has increased, particularly in patients with severe immunosuppression undergoing long-term treatment [2]. Hence, there is a global demand for new antifungal agents that eliminates fungal pathogens with minimal toxicity to the host. However, such ideal antifungals are difficult to develop because fungi are eukaryotes hence most substances toxic to fungi are also likely to be toxic to the human host [3]. Therefore, desirable antifungal agent should differentially target the fungal pathogen.

Metabolites from fungal isolates can also be screened for anticancer activities using target-oriented approaches. This has led to the approval of many molecularly targeted anticancer drugs. Development of cancer chemotherapeutics has focused on identifying compounds which target many cancer pathways while causing minimal toxicity to non-cancer cells [4]. Humans and yeast have similar genes including those known to be involved in cell proliferation and cancer [5]. *Saccharomyces cerevisiae* is used as a model for investigating many cellular processes in humans. These processes include cell cycle progression, DNA replication and segregation, maintenance of genomic integrity and stress responses. In cancer, these processes are affected by genetic and epigenetic alterations. Hence, yeast can be used as a suitable model organism for identifying novel compounds with anti-cancer activity during screening of chemical libraries [6].

In this project, extracts from marine endophytic fungi were partially purified to obtain fractions containing potential novel compounds. The effect of these fractions on proliferation and transcriptome of the human liver cancer cell line HepG2 were investigated. The evidence presented in this study will inform future effort to scale up the fermentation of this fungal isolate to purify the potential novel compounds for detailed characterization.

## Results

### Antifungal activities of the MEF 134 crude extract and the partially purified dichloromethane fraction

**Fig. 1:**
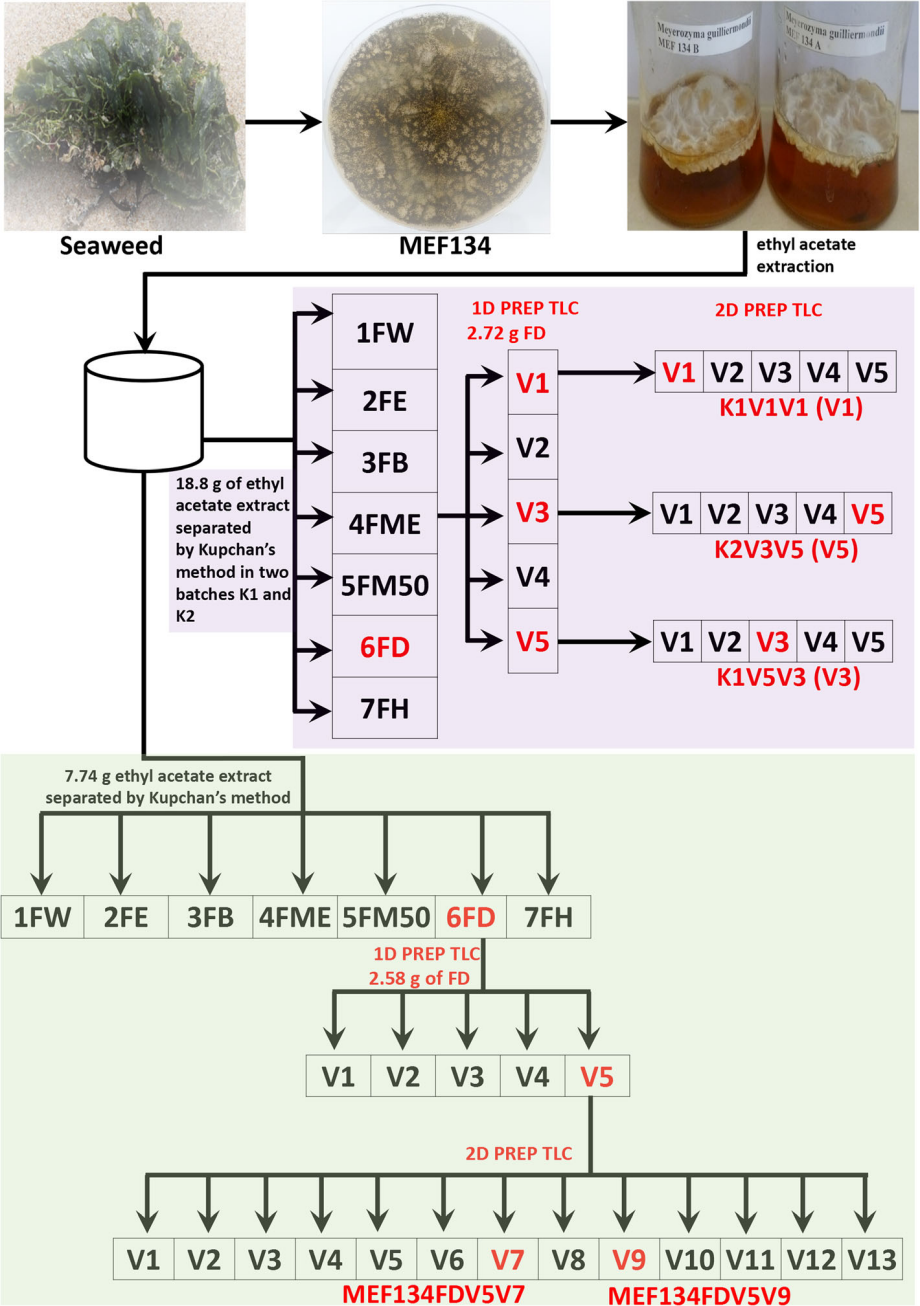
Schematic representation of the partial purification of crude extracts from MEF 134. MEF 134 was isolated from seaweed and was cultured in 20 L of broth. The crude extract obtained from the culture was separated by Kuchan fractionation and two rounds of preparative TLC. Samples marked in red were selected based on their antifungal activities and were further analysed using transcriptomics and mass spectromtery

A total of 143 morphologically distinct MEFs were isolated from mature seaweeds that were obtained from several beaches in Ghana. These MEF isolates were cultured in broth for 4 months and the secondary metabolites produced in each culture were extracted using ethyl acetate (Fig. 1). Preliminary screening of the crude extracts from these 143 MEF isolates revealed that the extract from the isolate designated MEF 134 showed the highest antifungal activity against *Candida albicans* (Table 1). However, the MEF 134 extract did not exhibit antifungal activity against the nonpathogenic fungi, *Saccharomyces cerevisiae* (Table 1). From these observations, a large-scale fermentation (20 L) culture was set up for the MEF 134 isolate in order to obtain sufficient crude extract for fractionation and purification of the constituent bioactive compounds. The detailed procedure for the Kupchan fractionation and the preparative thin layer chromatography (TLC) of the MEF 134 crude extract is summarized in Fig. 1. The full sample analysis by mass spectrometry is provided in Additional Information 1. Two rounds of Kupchan fractionation were conducted; 7.74 g of the MEF 134 crude extract was used as starting material for the first round and 18.3 g of the same extract for the second round of Kupchan fractionation (Fig. 1). The dichloromethane (DCM) fractions (FD) obtained from the first (FD-K1) and second (FD-K2) rounds of the Kupchan fractionation were active against both *Candida albicans* and *Saccharomyces cerevisiae* (Table 1). An amount of 2.58 g of the DCM fraction, obtained from the first round of the Kupchan fractionation procedure, was run on TLC plates coated with silica gel (Fig. 1). The FD K1V1 fraction that was obtained from the first round of TLC was only active against *Saccharomyces cerevisiae* while the FD K1V5 fraction was active against *Candida albicans* (Table 1). The second round of Kupchan fractionation yielded 2.72 g of the DCM fraction, which was also run on TLC plates. The FD K2V3 TLC fraction from the second round of Kupchan fractionation was active against both organisms recording zones of inhibition of 12.5 and 10 mm against *Candida albicans* and *Saccharomyces cerevisiae*, respectively (Table 1).

**Table 1:** Antifungal activity of MEF 134 Crude extract, DCM fractions, and fractions from the first round of preparative TLC

MEF 134 Samples	Zone of Inhibition in mm (Disc size 5 mm)	
	***Candida albicans***	***Saccharomyces cerevisiae***
Crude extract	9	0
FD-K1	12	10.5
FD-K2	8	6.5
FD K1V1	0	11.5
FD K2V3	12.5	10
FD K1V5	18.5	–

Fluconazole (5 μg) = 23 mm

Fluconazole was used as a positive control for the antifungal assay against *C. albicans*. – represents not available

**Fig. 2:**
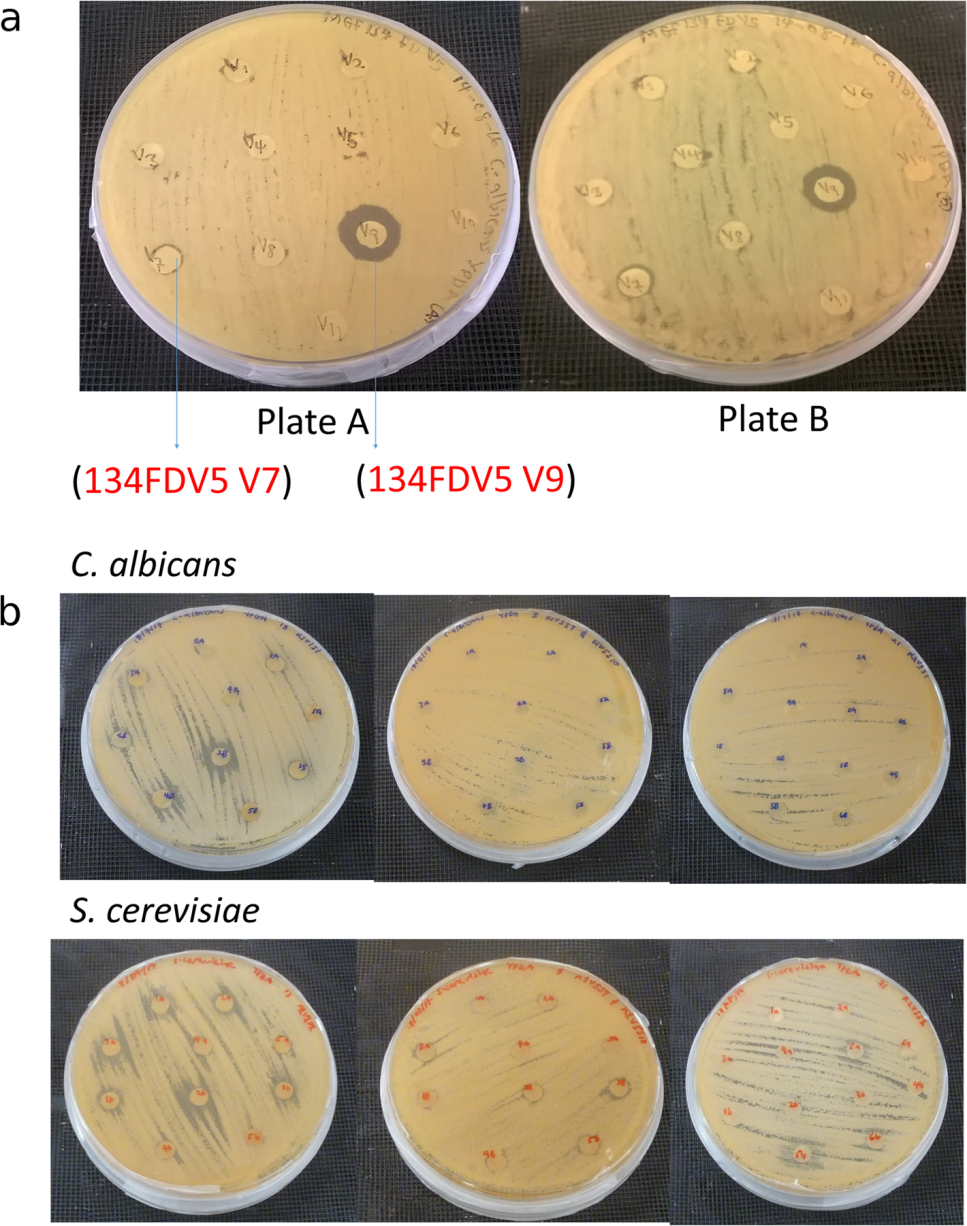
Representative images showing growth inhibition of *C. albicans* and *S. cerevisiae* by the antifungal fractions from MEF 134. **a** Growth inhibition of *C. albicans* by FDV5V7 and FDV5V9 fractions. **b** Plate cultures showing the antifungal activities of FDK1V1V1, FDK1V5V3 and FDK2V3V5 against *S. cerevisiae* and *C. albicans*

The fractions from the first round of preparative TLC, which exhibited antifungal activity, were re-spotted on silica gel TLC plates and the TLC procedure was repeated using the relevant solvent system. The FD K1V1V1, FD K1V5V3 and FD K2V3V5 fractions obtained from the second round of TLC analysis of FD K1V1, FD K1V5 and FD K2V3 did not show antifungal activity against *Candida albicans* but showed activity against *Saccharomyces cerevisiae* (Table 2; Fig. 2). Two other fractions from the second round of TLC analysis, FD V5V7 and FD V5V9, showed activity against *Candida albicans* (Table 2; Fig. 2a).

**Table 2:** Antifungal activity of the DCM fractions from the second round of preparative TLC

MEF 134 Fractions	Zone of Inhibition in mm (Disc size 5 mm)	
	***Candida albicans***	***Saccharomyces Cerevisiae***
FD V5V7	8	–
FD V5 V9	13	–
FD K1V1V1	0	10.5
FD K1V5V3	0	8.5
FD K2V3V5	0	11

Fluconazole (5 μg) = 23 mm

Fluconazole was used as a positive control for the antifungal assay against *C. albicans*. – represents not available

**Fig. 3:**
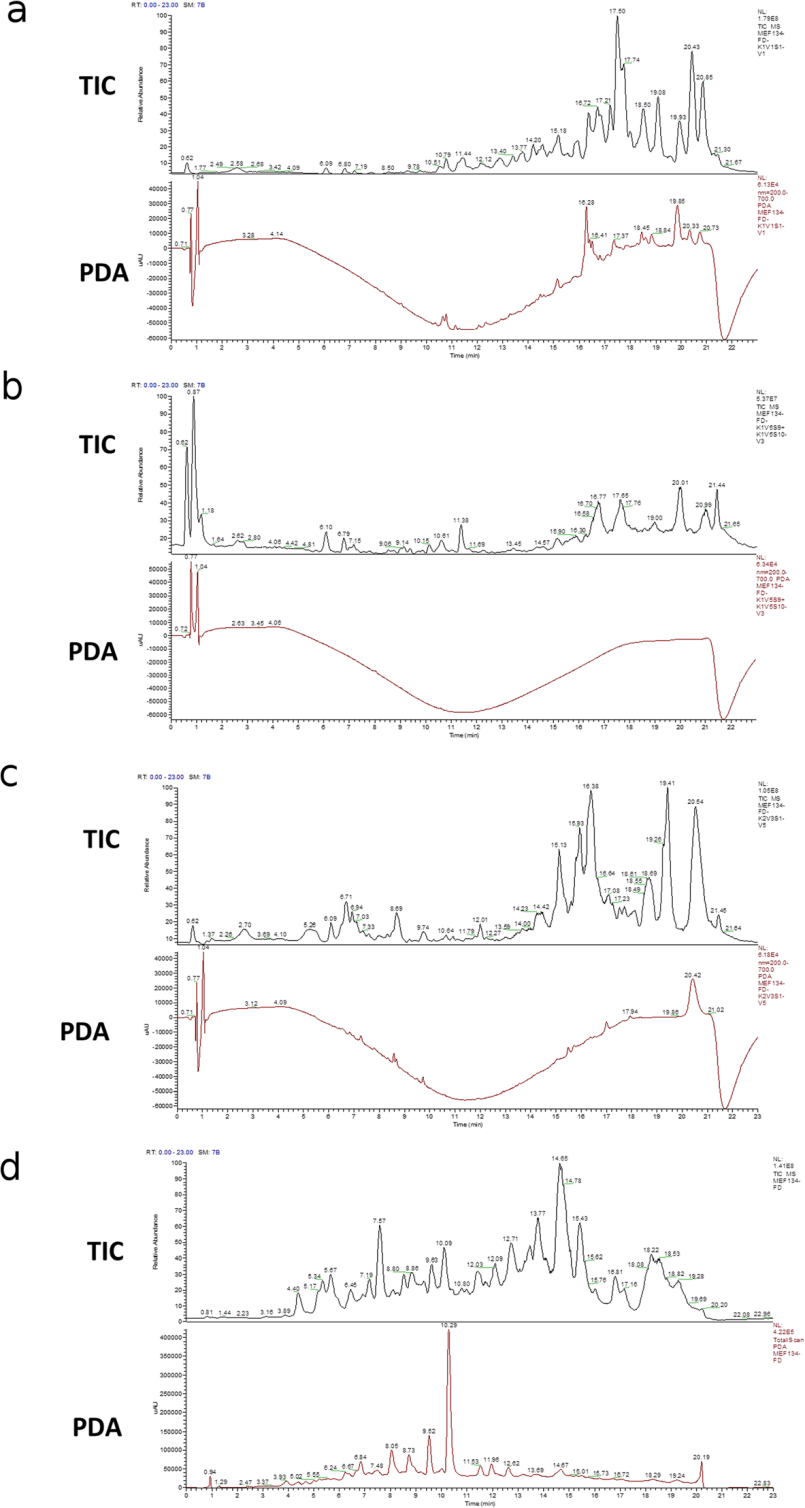
HPLC-HRMS full scan analysis of selected DCM fractions from the second round of preparative TLC analysis. The fractions that were used for the analysis were FD K1V1V1 (**a**), FD K1V5V3 (**b**), FD K2V3V5 (**c**) and the DCM fraction from the MEF 134 crude extract (**d**). Spectra from total ion chromatogram (TIC) and photodiode array (PDA) detectors are shown for each fraction

The second round of Kupchan fractionation was conducted with 18.3 g of extract as new starting material obtained repeating the extraction of the matured culture. Here 2.7 g of the dichloromethane fraction obtained was again separated on the preparative plates made of aluminum oxide. It turned out to be difficult to elute the fractions from the aluminum oxide stationary phase. Hence 12 different solvent systems were employed to perform the elutions since it takes considerable amount of time and resources to obtain the extract. The active fractions obtained (Fig. 2b) were analyzed with an antifungal bioassay and High-performance liquid chromatography (HPLC) together with High resolution mass spectrometry (HRMS) (Table 2 and Fig. 3).

### HPLC- HRMS analysis of MEF 134 DCM fractions

The DCM fraction from the MEF 134 crude extract (FD) and selected fractions from the second round of the preparative TLC (FD K1V1V1, FD K1V5V3 and FD K2V3V5) were analyzed by high performance liquid chromatography coupled with high resolution mass spectrometry (HPLC-HRMS). The HPLC-HRMS analysis of the FD K1V1V1 fraction revealed 11 major peaks; 5 of these 11 major peaks were important for this study because of their interesting fragmentation patterns (Fig. 3a). The fragmentation pattern of the masses suggest that the compounds have complex structures (Data not shown). Additionally, the FD K1V5V3 fraction had 6 major peaks and three of these major peaks were also interesting because of the fragmentation pattern (Fig. 3b). The FD K2V3V5 fraction generated 11 major peaks (Fig. 3c) while 17 major peaks were detected for the DCM fraction from the MEF134 crude extract (FD; Fig. 3d).

### Effect of the MEF 134 fractions on proliferation of HepG2 cells

**Fig. 4:**
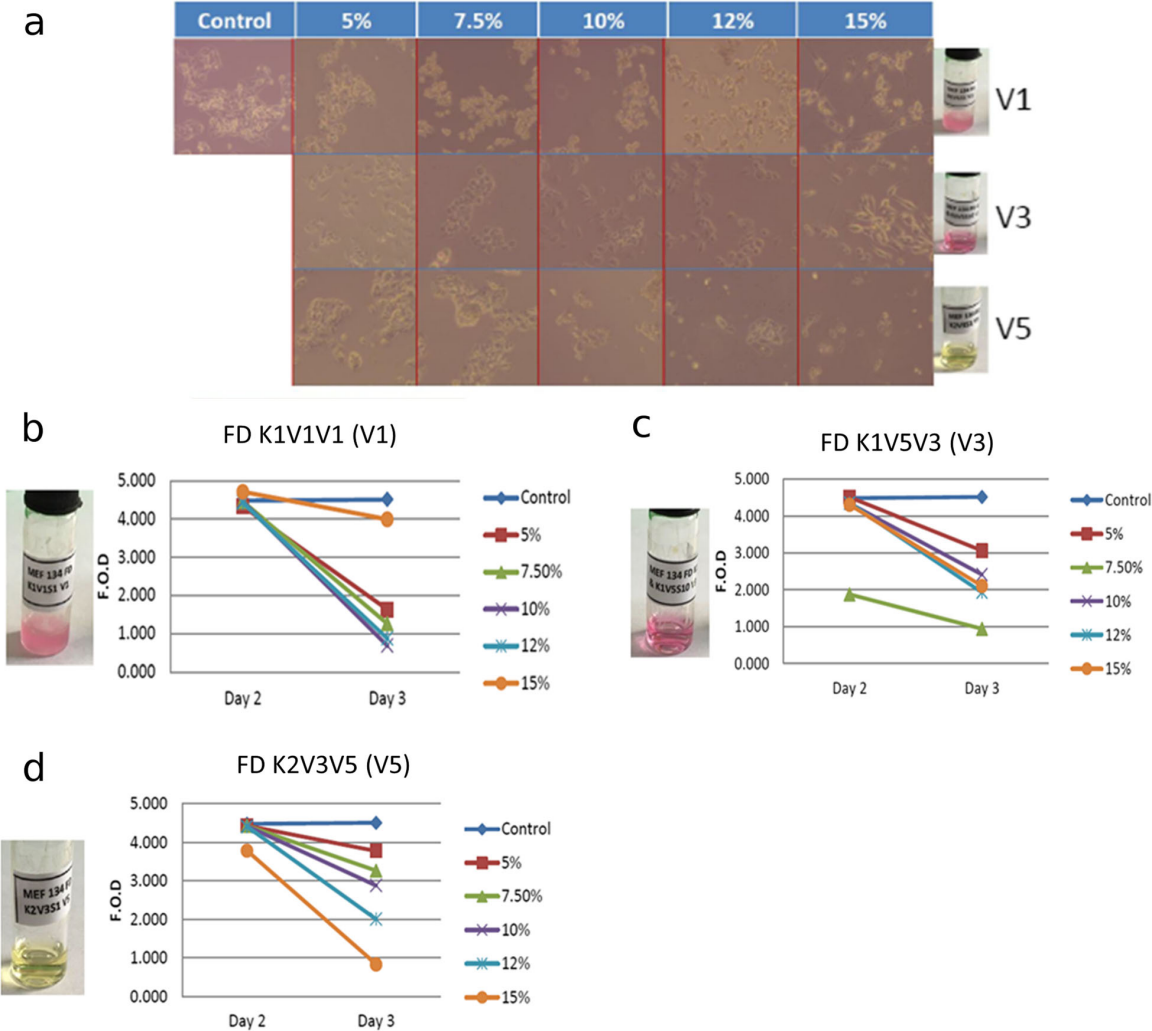
Inhibition of proliferation of HepG2 cells by the three partially purified fractions from the MEF 134 crude extracts. **a** Representative light microscopy images of HepG2 cells treated with the MEF 134 fractions. The dose of each fraction used for the assay is indicated in percentages (w/v). **b**-**d** Quantification of the anti-proliferative effect of the fractions. The fractions used for the assay were FD K1V1V1 (V1), FD K1V5V3 (V3) and FD K2V3V5 (V5). All measurements were performed in biological duplicates. All three fractions had antifungal activity against *S. cerevisiae* only

The effect of the FD K1V1V1 (V1), FD K1V5V3 (V3) and FD K2V3V5 (V5) fractions on proliferation of HepG2 cells was also investigated. All the three fractions reduced proliferation of HepG2 cells. The cells were first observed using light microscopy, comparing control cells to the treated cells. It could be observed how the untreated cells grew in the typical cell morphology; triangular and growing in islands or clumps, whereas the treated cells were somehow dissociated, were rounder in shape and could be found in smaller clumps, as well as single cells. The extent of reduction in cell proliferation seemed to be dose-dependent for fraction V5 whereas there were deviations from the dose-dependent effect for fractions V1 and V3 (Fig. 4). In the case of V1, a concentration of 5% massively reduced the proliferation of HepG2 cells. Therefore, we decided to reduce the input concentrations of all compounds for all further experiments in order to maintain cell proliferation.

### Effect of the MEF 134 fractions on viability of HepG2 cells

**Fig. 5:**
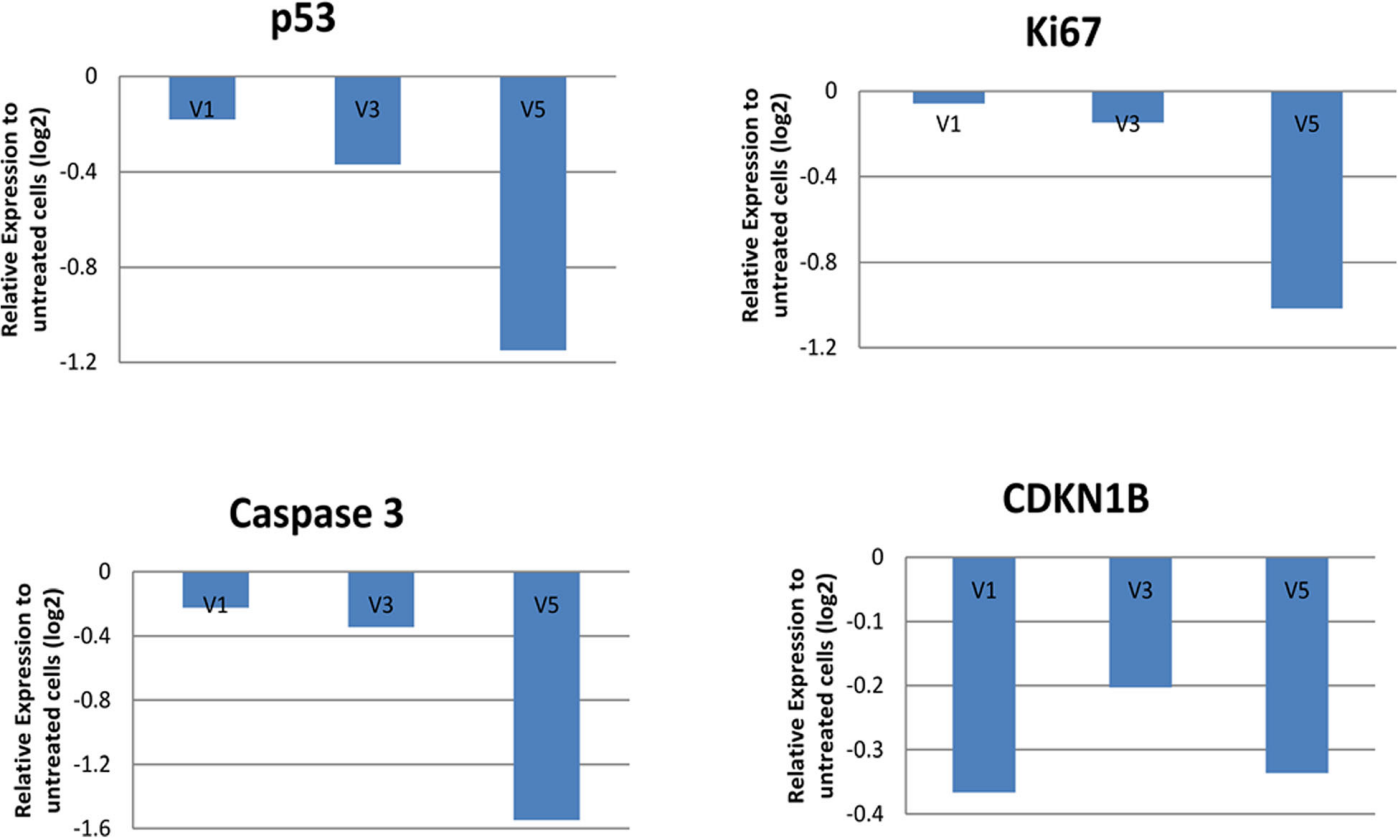
Reduction in the expression of markers of viability in HepG2 cells treated with MEF 134 fractions. HepG2 cells were treated with 2% of either FD K1V1V1 (V1), FD K1V5V3 (V3) or FD K2V3V5 (V5) and mRNA expression for the indicated genes was determined by qRT-PCR. Experiments were performed in biological duplicates. Gene expression was normalized to the RPL37A gene and fold change was calculated relative to the untreated cells

HepG2 cells were treated with 2% of the partially purified MEF 134 fractions (FD K1V1V1 (V1), FD K1V5V3 (V3) and FD K2V3V5 (V5)) in order to assess the effect of these fractions on cell viability. Following treatment, the mRNA expression profile of 4 key markers (P53, KI67, Caspase3, and CDKN1B) of cell viability were analyzed and compared to the expression profile from the untreated cells (Fig. 5). Expression of all the 4 selected genes was reduced in the treated HepG2 cells relative to untreated cells.

### Transcriptome analysis of HepG2 cells treated with the MEF 134 fractions

**Fig. 6:**
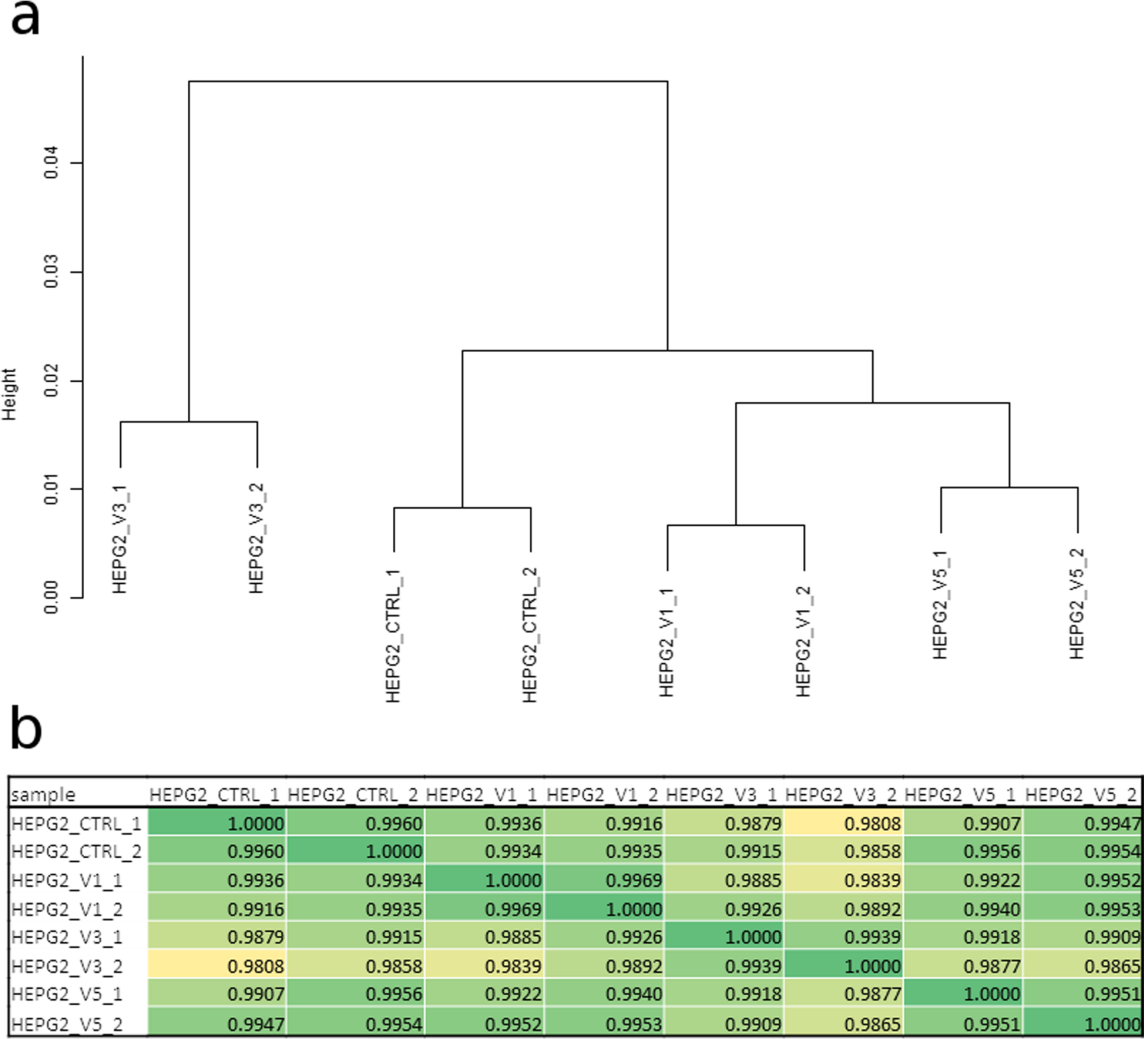
Analysis of variability in transcriptome of untreated and treated HepG2 cells. **a** Clustering analysis of RMA-normalized microarray data from untreated and treated HepG2 cells. The clustering analysis was conducted using complete linkage as agglomeration method and Pearson correlation as similarity measure. **b** Correlation analysis of the three treatment conditions and the untreated HepG2 cells. The Pearson correlation coefficient between each treatment or untreated condition was estimated, as indicated in the table. All correlation coefficients were close to the possible maximum of 1 demonstrating a high overall similarity of the samples

Cluster analysis of RMA-normalized microarray data showed that duplicates of the treated and untreated HepG2 cell samples clustered together, as expected (Fig. 6a). HepG2 cells treated with V1 and V5 were together in a cluster and thus had the highest similarities between experiments. The V1-V5 cluster was further extended by HepG2 untreated cells demonstrating higher similarity of V1- and V5-treated cells with the untreated cells compared to the V3-treated HepG2 cells. HepG2 cells treated with V3 clustered separately suggesting that V3 treatment had the highest effect at the transcriptome level. This is reflected by the Pearson correlation analysis which indicated that the V3 treatment had the lowest correlation to all other samples (Fig. 6b). Moreover, the V1 treatment had the highest correlation with V5 treatment although there was intra-sample variability. Furthermore, one of the V1 samples had a slightly higher correlation to control compared to the V5 treated sample. Overall, the Pearson correlation coefficients ranged between 0.9808–0.9969 and between 0.9939–0.9969 for duplicates.

**Fig. 7:**
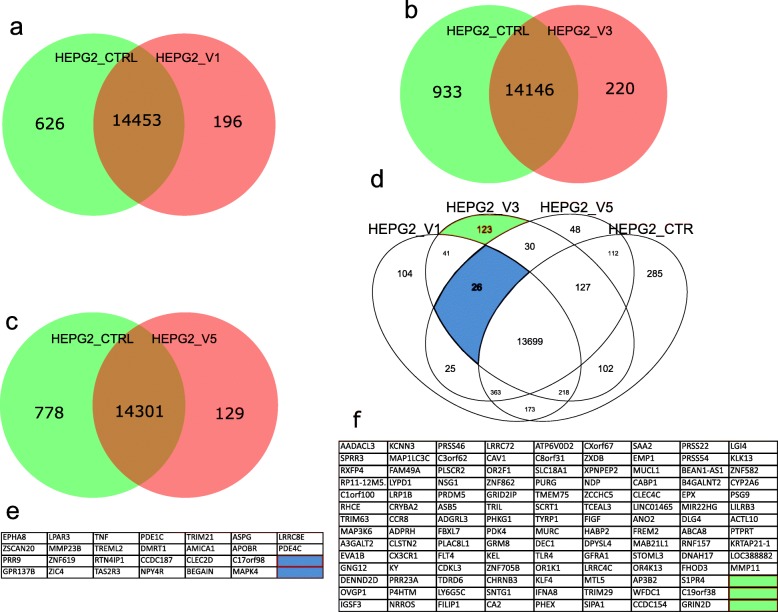
Comparison of the number of HepG2 genes that were commonly expressed or uniquely expressed for each treatment condition and the untreated cells. **a** Comparison of V1-treated HepG2 cells and untreated HepG2 cells. **b** Comparison of V3-treated HepG2 and untreated HepG2 cells. **c** Comparison of V5-treated HepG2 cells and untreated HepG2 cells. **d** Four-way comparison of the number of HepG2 genes expressed in all three treatment conditions and the untreated cells. The segment marked blue represent the common gene expression signature for the treated and untreated HepG2 cells. Expressed genes were detected using a detection *p* value threshold of *p* < 0.05. **e** 26 genes related to the blue segment in (**d**) of expression in all treatments but not in control. **f** 123 genes expressed exclusively in V3 related to the green segment in (**d**)

Analysis of the gene expression at a threshold *p* value < 0.05 showed that most of the genes that were expressed in HepG2 transcriptome were common to the treated and untreated conditions. In addition, fewer genes were expressed in the treated HepG2 cells compared to the untreated cells (Fig. 7, Tables S1-S4). Pairwise comparison of the HepG2 cells treated with V1 versus the untreated HepG2 cells generated the highest number of genes (14453) that were commonly expressed (Fig. 7a, Additional Table 1) while the comparison of HepG2 cells treated with V3 versus the untreated HepG2 cells generated the lowest number of genes (14146) that were commonly expressed under these conditions (Fig. 7b, Additional Table 2). These observations might serve as a confirmation that treatment of HepG2 cells with V3 had the highest effect on the transcriptome of HepG2 (Fig. 7b, Additional Table 2). A 4-way comparison of the genes expressed in the treated conditions and the untreated cells also confirmed that most of the genes (13699) were commonly expressed in all the three treatment conditions and the untreated cells (Fig. 7d, Additional Table 4). The 4-way comparison also revealed that 285 genes were exclusively expressed in the untreated cells while 123 genes (Fig. 7f), 104 genes and 48 genes were exclusively expressed in the V3, V1 and V5 treatment conditions, respectively.

### Transcription factor analysis

Transcription factors motif enrichment analysis employing the CentriMo tool from the MEME suite identified several transcription factors. In line with expectations the TATA-box binding protein (TBP) appears on top of several compounds’ analysis results were sorted by significance of the E-value. Transcription factors of the HOX (e.g. HOXA13, HOXC13), KLF (e.g. KLF5, KLF16), SP (e.g. SP1, SP2), CDX (e.g. CDX1, CDX2) TF families appear as prominent results across the components (Additional Tables 13–19). Interestingly, exclusively in the most significant TFs in down-regulation via the V3 component several TFs of the SOX family (Sox7, Sox13, Sox15, Sox17, Sox18) and POU3F1 appear (Additional Table 19).

### Overrepresented pathways and gene ontologies (GOs)

**Fig. 8:**
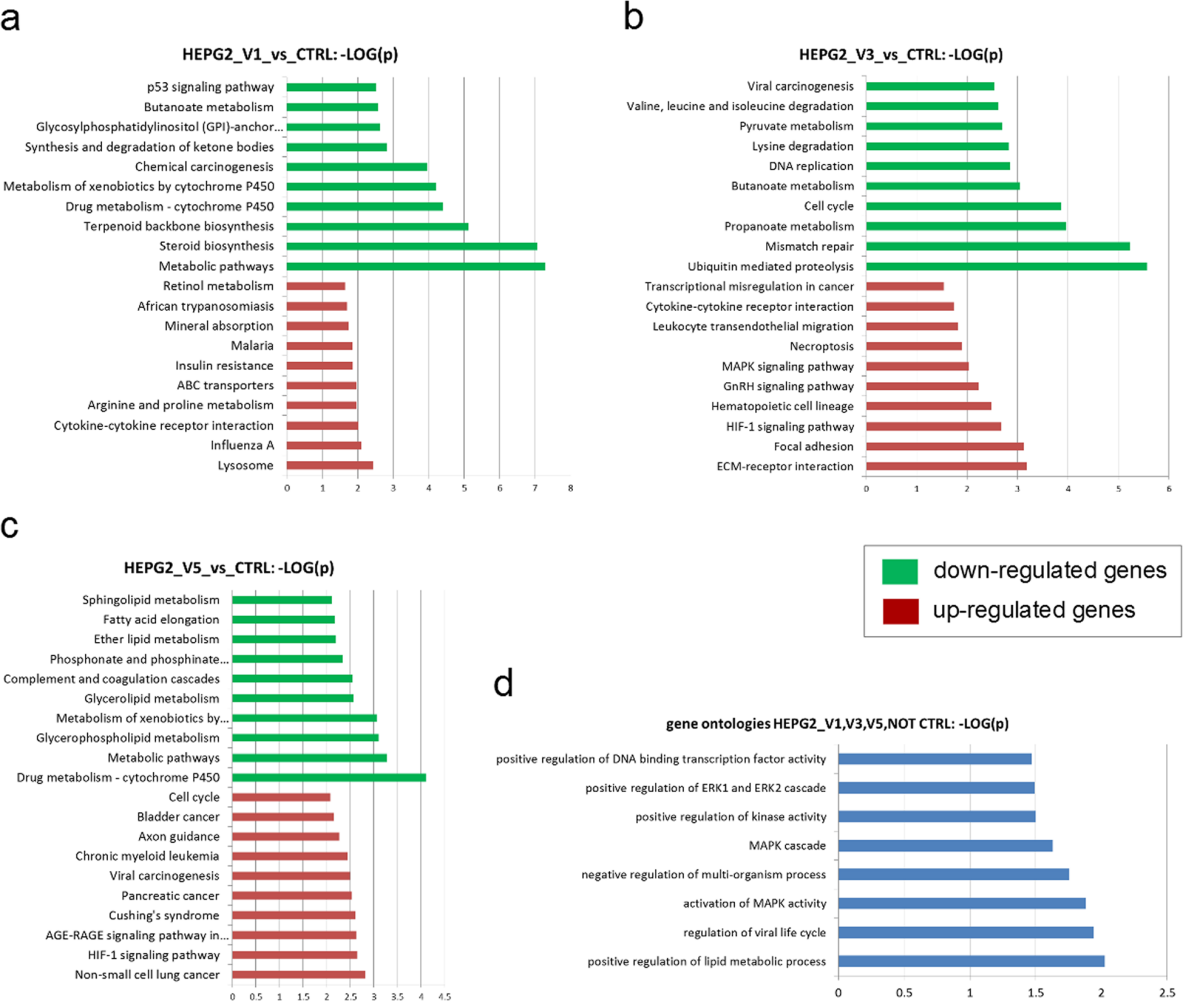
KEGG pathways affected by treatment of HepG2 cells with partially purified fractions from MEF 134 extract. **a** Cellular pathways up-regulated or down-regulated in V1-treated cells relative to the untreated cells. **b** Cellular pathways upregulated or down-regulated in V3-treated cells relative to the untreated cells. **c** Cellular pathways upregulated or down-regulated in V5-treated cells relative to the untreated cells. In (**a-c**), green and red bars indicate overrepresentation of down-regulated and up-regulated genes, respectively. **d** Gene ontologies for the genes commonly expressed in all three treatment conditions. In (**a-d**), the bar charts show the negative logarithm (base 10) of the *p*-value; higher values correspond to higher significance. Overrepresentation of KEGG pathways and gene ontologies were analyzed using the hypergeometric test

Overrepresentation analysis for KEGG pathways was performed to identify genes that were either up-regulated (ratio < 0.75; *p*-value < 0.05) or down-regulated genes (ratio > 1.33; *p*-value < 0.05) from the intersection sets of the pairwise Venn diagrams. The analysis demonstrated that treatment of HepG2 cells with either V1, V3 or V5 had impact on many cancer- and metabolism-related pathways (Fig. 8a-c, Tables S5-S10).

### Genes and associated pathways regulated by treatment with V1

For the V1-treated HepG2 cells, cancer-related pathways, such as p53, chemical carcinogenesis and metabolic pathways such as cytochrome P450 metabolism emerged in the down-regulated genes while metabolic pathways for arginine, retinol and insulin resistance emerged in the up-regulated genes (Fig. 8a, Tables S5-S6). Visual inspection of the pathway charts showed that most of the steroid biosynthesis pathway (S1 Fig) was down-regulated. Interestingly, for pathways involved in drug metabolism such as cytochrome P450 (S2 Fig), the genes encoding ALDH3B, ADH4 and GSTA1 were down-regulated.

### Genes and associated pathways regulated by treatment with V3

For the V3-treated HepG2 cells, cancer-related pathways such as cell cycle, mismatch repair and viral carcinogenesis emerged in the down-regulated genes whereas cancer-related pathways such as MAPK-signaling and transcriptional misregulation were detected for the up-regulated genes (Fig. 8b, Tables S7-S8). The chart of the cell cycle pathway (Fig. S4) showed down-regulation of most genes involved in this pathway thus suggesting reduced cell cycle activity. In all subsystems (Hepatitis B/C, Epstein-Barr virus, human Papillomavirus, human T Lymphotrophic virus Type I and Kaposi’s sarcoma-associated Herpesvirus) of the viral carcinogenesis pathway (Fig. S5) genes were significantly down-regulated.

### Genes and associated pathways regulated by treatment with V5

For the V5-treated HepG2 cells, cancer-related pathways such as cell cycle and other various cancers emerged in the up-regulated genes while various metabolic pathways including cytochrome P450 metabolism emerged in the down-regulated genes (Fig. 8c). Contrary to the V3-treated HepG2 cells, the up-regulated genes in the V5-treated cells were significantly overrepresented in the cell cycle pathway. Moreover, the TGFB1 and the cyclin-dependent kinase inhibitors CDKN1A and CDKN2C were up-regulated (Fig. S6, Tables S9-S10).

### Common gene ontologies over-represented in all treatments

The set of genes that were expressed in the three treatment conditions, but not in the untreated HepG2 cells, were used to identify the specific gene expression that distinguishes the treated cells from the untreated cells. The set of 26 genes (Fig. 7e) expressed in common in all treatment conditions (V1, V3 and V5) but not in the HepG2 control revealed overrepresentation of gene ontologies annotated with cancer-associated MAPK/ERK-signaling (Fig. 8d, Additional Table 11). A detailed listing and description of these 26 genes generated with the DAVID annotation tool [7] is provided in Additional Table 12. In brief, there are genes which are (i) transcription factors (ZIC4, DMRT1, ZSCAN20, ZNF619, TRIM21), (ii) cell signaling associated (TREML2, PRR9, MAPK4, APOBR, EPHA8, GPR137B, NPY4R) and (iii) tumor necrosis factor (TNF) which is involved in both inflammation and apoptosis.

### Analysis of protein interaction networks activated by the treatment conditions

**Fig. 9:**
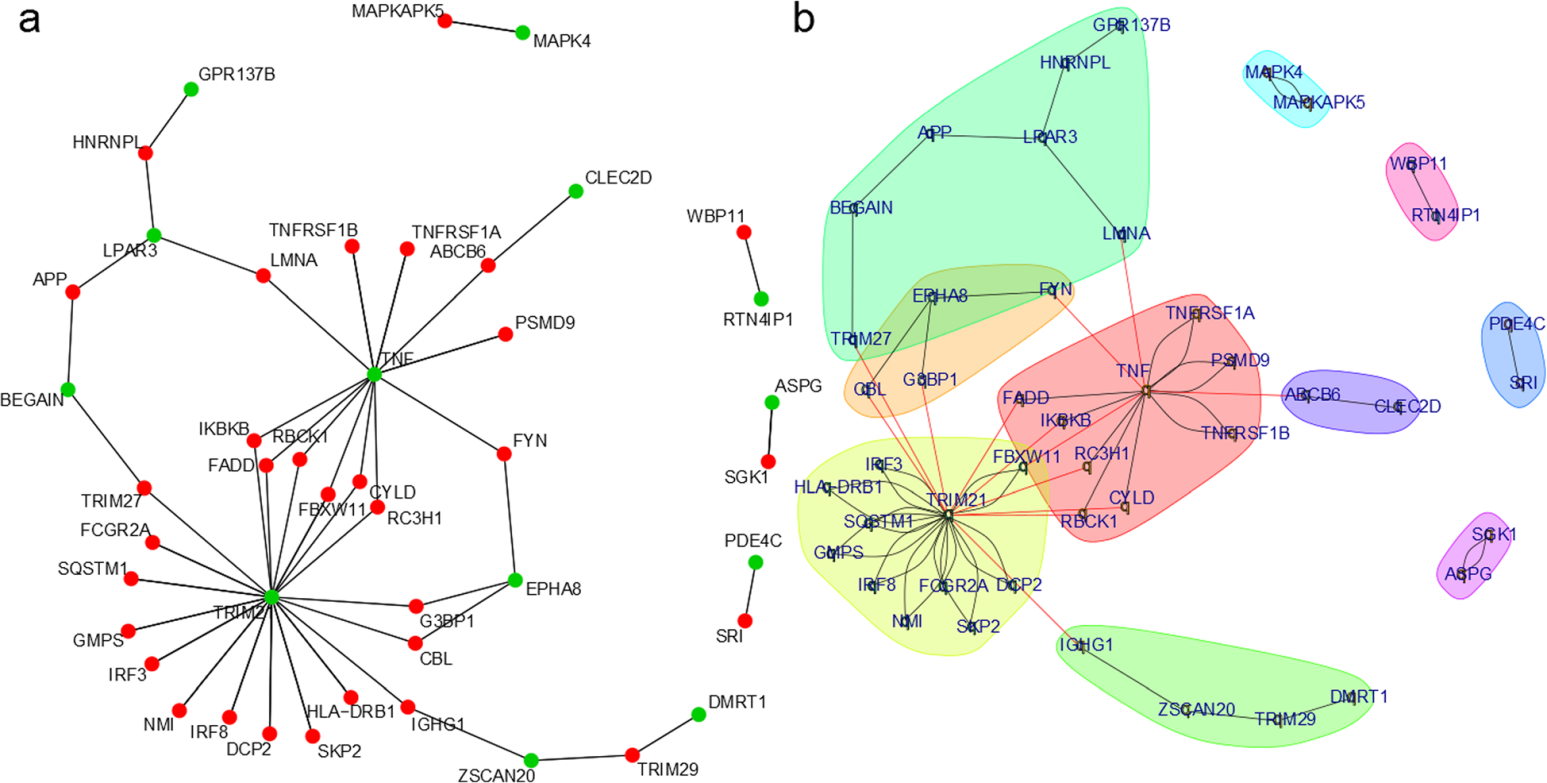
Protein interaction networks activated by treatment of HepG2 cells with partially purified fractions from MEF 134 extract. **a** Interconnections of the commonly expressed genes in treated HepG2 cells to a network with interactions in the Biogrid database. Genes from the original 26 gene set are colored green while the genes added as Biogrid interactions are colored red. **b** Community clustering of the protein interaction network. The Biogrid database was used to construct protein interaction networks using the genes expressed in common in all treatments conditions (V1, V3 and V5) but not in the untreated HepG2 cells

The set of 26 genes that were commonly expressed in all the three treatment conditions (V1, V3 and V5) but not in the untreated HepG2 cells were further analyzed for protein interaction networks based on the Biogrid database. Most of these 26 genes (green nodes, Fig. 9a) had interactions with other proteins, as reported in the Biogrid database (red nodes, Fig. 9a). Within the network, communities with similar features via community clustering were analyzed (Fig. 9b, Fig. S1). Several communities including those characterized by tumor necrosis factor (TNF, red), mitogen-activated protein kinase 4 (MAPK4, skyblue), tripartite motif containing 21 (TRIM21, yellow) and amyloid beta precursor protein (APP, green) were identified.

## Discussion

In this study, crude extract from the MEF 134 isolate showed antifungal activity against the pathogenic *Candida albicans* but not against *Saccharomyces cerevisiae*. Kupchan fractionation of the crude extract, followed two rounds of preparative TLC generated partially purified fractions that showed activity against either one or both of the fungal test organisms. These observations demonstrate the presence of antifungal agents and possible antagonists of these agents in the crude extract obtained from MEF 134.

The partially purified fractions were analyzed via HPLC-HRMS and complex patterns including multiple peaks were detected in the mass spectra. These fractions were also evaluated using the highly sensitive microarray-based analysis of gene expression which is capable of identifying prospective compounds that can serve as new leads for the development of new anticancer chemotherapy. The ability to generate milligram quantities of natural products was extremely resourceful and intensive and it provides a key evidence for making future commitments.

### Proliferation and viability of HepG2 after treatment with the fractions

Putative anticancer properties of the fractions were implicated by the reduction in proliferation of the human liver cancer cell line HepG2. Specifically, expression of 4 viability markers; *P53*, *KI67*, Caspase3, and *CDKN1B* was reduced in the HepG2 cells treated with the fractions compared to untreated cells. The reduction of *KI67* expression, which is a marker for proliferation [8], correlated positively with the outcome from the proliferation assay. The concomitant reduction of Caspase 3 indicated that these observations might not be related to increased apoptosis [9]. Interestingly, *p53* and *CDKN1B*, which are both negative regulators of cell cycle progression [10, 11], were down-regulated. This is might be due to complex interaction mechanisms and could reflect stress reactions. Fraction V5 showed the strongest negative effect on all genes except *CDKN1B*, indicating that it had the highest impact on overall cell survival.

### Transcriptome analysis

Analysis of the genes expressed exclusively in the V1-, V3- or V5-treated HepG2 cells but not in the untreated cells revealed many overrepresented cancer-related pathways. Moreover, the genes that were expressed in common for all the treatment conditions, but not in the untreated HepG2 cells, were used to construct a protein interaction network.

### Pathways regulated by treatment with V1

The down-regulation of ALDH3B in the drug metabolism pathway involving *cytochrome P450* (Additional Fig. 2) supports the central effect of cyclophosphamide as a chemotherapeutic agent, which relies on phosphoramide mustard produced only in cells that have low levels of ALDH [12]. Phosphoramide mustard induces apoptosis by causing DNA crosslinks between and within DNA strands at guanine N-7 positions. In the *p53 signaling pathway*, down-regulated genes were in the cancer-suppressing sub-paths leading to cell cycle arrest, prevention of DNA damage and inhibition of angiogenesis, metastasis and DNA repair. However, the p53 negative feedback sub-path was also down-regulated which might eventually lead to up-regulation of the p53 pathway.

### Pathways regulated by treatment with V3

The down-regulation of most genes involved in the cell cycle pathway (Additional Fig. 4) in V3-treated HepG2 cells is in accordance with the down-regulation of proliferation upon V3 treatment. The significant down-regulation of genes in all subsystems of the *viral carcinogenesis* pathway (Additional Fig. 5) indicate the inhibitory effect of the V3 treatment on many viral induced cancer hallmarks, e.g. down-regulation of proliferation by SKP2 in the Epstein-Barr virus.

### Pathways regulated by treatment with V5

For V5-treated HepG2 cells, the up-regulation of genes from the *cell cycle* pathway, particularly *TGFB1*, *CDKN1A* and *CDKN2C* (Additional Fig. 6, Additional Tables 9–10), is in agreement with reports that the usual association of the cyclin-CDK complexes with *CDKN1A* (alias *p21*) is absent in most transformed cells [13]. More general, cyclin-dependent kinase inhibitors are considered to function often as tumor-suppressors and lead to cell cycle arrest [14]. Thus, it confirms the reduction of proliferation upon treatment of HepG2 cells with the V5 fraction.

### Analysis of protein interaction networks activated by the treatments of HepG2 with the fractions

The protein interaction network derived from the set of 26 genes expressed in common in all treatments (V1, V3 and V5) but not in the untreated HepG2 cells revealed communities (Fig. 9b, Additional Fig. 1) with similar network features including those characterized by tumor necrosis factor (TNF, red), mitogen-activated protein kinase 4 (MAPK4, skyblue), tripartite motif containing 21 (TRIM21, yellow) and amyloid beta precursor protein (APP, green). The protein interactions of brain enriched guanylate kinase associated (BEGAIN) and lysophosphatidic acid receptor 3 (LPAR3) with APP, retrieved from Biogrid, have been reported as results from high-throughput in vitro experiments [15]. The role of aberrant expression of LPARs in cancer has also been established [16]. Although APP is mostly associated with Alzheimer’s disease, many recent studies have described its impact on cancer [17–19] . The tumor-suppressing activity of TRIM21 has been reported although there may be variability between different cancer types and treatment conditions. TRIM21 is a tumor-suppressor in hepatocellular carcinoma [20, 21] and it also down-regulates PAR4 (a tumor suppressor) in pancreatic cancer in the presence of cisplatin [22]. Even though the relevance of TNF in cancer has been proven, there are therapeutic effects and tumor advancing properties of its inflammatory response [23]. Moreover, anti- and pro-ontogenic properties has been reported for MAPK4 (alias ERK4), thus extending established knowledge about the major role typical MAP kinase pathways ERK1/2-MEK1/2 play in cancer [24]. Furthermore, MAPK-signaling is downstream of TNF [23] thus emphasizing the importance of these components of the network.

## Conclusions

In this study, crude extract from a marine endophytic fungal culture was fractionated to obtain partially purified fractions. These fractions showed antifungal activity against *C. albicans* or *S. cerevisiae* alone or both and reduced proliferation of the human liver cancer cell line HepG2. Detailed transcriptome analysis revealed that several cancer- and metabolism-related pathways and gene ontologies were regulated by treatment of HepG2 cells with the fractions. A protein interaction network distinguishing the fraction-treated HepG2 cells from the untreated HepG2 cells was also constructed. Major functional components of this network (TNF, MAPK, TRIM21 and APP) were associated with metabolism and cancer. It is anticipated that the data from this study would propel synthesis of the prominent compounds detected from the partially purified fractions. The synthesis of these compounds would be instrumental in identifying new compound(s) exhibiting antifungal or anti-proliferative activities. Such compounds could be used as starting material for the development of novel antifungal and anti-cancer drugs.

## Methods

### Large-scale fermentation of MEF 134 and extraction of secondary metabolites

Pure isolates of MEF 134 were inoculated into 20-l culture vessels containing 10 l of Yeast extract, Peptone, Malt, Dextrose (5 g/l; 5 g/l; 5 g/l; 30 g/l, respectively) broth prepared with filtered seawater. The inoculated broth was incubated at 30 °C for 4 months [25]. The secondary metabolites that were produced in the fermentation culture were extracted using equal volume of ethyl acetate [25]. The extracted secondary metabolites were dried at 45 °C under reduced pressure using a rotary evaporator (Buchi). The concentrated metabolites were reconstituted in methanol.

### Fractionation of MEF 134 crude extracts

The MEF 134 crude extract was fractionated using a modified Kupchan fractionation method followed by preparative Thin Layer Chromatography (TLC). The modified Kupchan fractionation was performed by partitioning the crude extracts among seven different solvent systems [26]. These solvents included; water, ethyl acetate, butanol, methanol & ethyl acetate, 50% methanol, dichloromethane and hexane. The fractions obtained from these solvents were designated FW (water fraction), FE (ethyl acetate fraction), FB (butanol fraction), FME (methanol & ethyl acetate fraction), FM50 (50% methanol fraction), FD (dichloromethane fraction) and FH (hexane fraction). The 7 fractions from each Kupchan fractionation was dried and reconstituted in either methanol (FW, FB, FM50) or ethyl acetate (FE, FME, FD, FH). The first round of preparative TLC was performed using 50 ml of mobile phase (35 ml ethyl acetate, 10 ml acetonitrile and 5 ml petroleum ether). The bands were cut and the compounds were eluted from the silica gel using methanol. The second round of preparative TLC was performed using 37.5 ml of petroleum ether and 12.5 ml of ethyl acetate as mobile phase for bands obtained near the solvent front of the TLC plate while 10 ml of ethyl acetate, 40 ml of acetonitrile and 0.3 ml of methanol was used as mobile phase for bands that showed minimal migration during the first round of preparative TLC. Dried and developed plates were visualized under UV light at high (365 nm) and low (254 nm) wavelengths.

### Determination of the antifungal activity of the MEF 134 crude extract and dichloromethane fractions

Antifungal activity of the crude extract and the dichloromethane fractions (FD) were tested by the agar plate disc-diffusion method. A volume of 30 μl (0.05 g) and 120 μl (0.2 g) from stock solution of crude extract and FD, respectively, were added on to sterile filter paper disc which were 5 mm in diameter. All other fractions were tested by this method at 0.01 g/disc. The discs were allowed to air-dry prior to the assay. Overnight cultures of *Saccharomyces cerevisiae* and *Candida albicans*, grown in 50 ml nutrient broth, were diluted to an optical density at 600 nm (OD_600_) of 0.7. The diluted cultures were uniformly inoculated on to YPDA (Yeast extract, 10 g/l; Peptone, 20 g/l; Dextrose, 20 g/l; Agar, 20 g/l) plates and the discs were placed at defined positions on the agar plate. Zones of inhibition were measured in mm, 12 h after incubation of inoculated plates at 30 °C.

### Determination of cytotoxicity of the MEF 134 fractions against HepG2 cells

Hepato-carcinoma (HepG2) cells (ATCC®HB-8065TM) were cultured in DMEM low glucose, 10% FCS, 1% Penicillin/Streptomycin (P/S) (all Gibco) at 37 °C and 5% CO_2_ in a humidified atmosphere. Cells were split with trypsin (Gibco) when they reached confluency. Monolayers were treated with the FD K1V1V1, FD K1V5V3 and FD K2V3V5 fractions at the indicated concentrations (5, 7.5, 10, 12 and 15%; w/v). Cell proliferation was determined by the resazurin metabolic assay [27]. After treatment for 2 or 3 days with the indicated concentrations of the three fractions, cells were incubated for 4 h with fresh medium containing 10% of resazurin solution consisting of 0.1 mg/mL resazurin (Sigma-Aldrich) in phosphate buffer saline (PBS) (Gibco). Resazurin reduction was measured with a spectrophotometer (Bio-tek instruments) at 570 and 600 nm. A final resazurin value (F.O.D.) was calculated as the difference between the O.D. 570/O.D. 600 nm of the treated sample and that of the negative control (resazurin media incubated for 4 h in the absence of cells). The procedure was carried out for 3 days at the same time, in duplicate.

### RNA isolation and quantitative real time PCR (qRT-PCR)

HepG2 cells were lysed in 300–500 μl Trizol and total RNA was isolated with the Direct-zol™ RNA Isolation Kit (Zymo Research) according to the user’s manual including the optional on-column DNase digestion. The TaqMan Reverse Transcription (RT) Kit (Applied Biosystems) was used for cDNA synthesis. Real time PCR was performed in technical triplicates of biological duplicates using Power SYBR Green Master Mix (life technologies) on a VIIA7 (life technologies). Mean Ct values were calculated and normalized to RPL37A as a housekeeping gene. Fold change was calculated relative to the untreated control. The data were shown as mean values (log2).

### Transcriptome analysis

Untreated HepG2 cells and cells treated with FD K1V1V1 (V1), FD K1V5V3 (V3) and FD K2V3V5 (V5) were hybridized in duplicates on the Affymetrix Human Clariom S Array (Affymetrix, Thermo Fisher Scientific) at the BMFZ (Biomedizinisches Forschungszentrum) core facility of the Heinrich-Heine Universität, Düsseldorf. Data analysis of the Affymetrix raw data was performed in the R/Bioconductor [28] environment using the package oligo [29]. The obtained data were background-corrected and normalized by employing the Robust Multi-array Average (RMA) method from the package oligo. The heatmap.2 function from the gplots package was employed to generate hierarchical clustering and heatmaps using Pearson correlation as similarity measure and color scaling per rows containing genes [30]. Besides the dendrogram (Fig. 6a) and table of Pearson correlations (Fig. 6b) quality was controlled by pairwise scatter plots of logarithmic (base 2) expression values (Additional Fig. 8) assessing the variance between duplicates. Venn diagrams were drawn based on gene expression employing package VennDiagram [31]. A gene was considered to be expressed when its detection *p* value was less than 0.05. The detection p value was calculated as described in the supplementary methods in Graffmann et al. [32]. Up-regulated genes were detected via the criteria detection-*p*-value in compound-treatment and control less than 0.05, limma-p-value for differential expression less than 0.05 and ratio greater than 1.33. Down-regulated genes were detected via the criteria detection-p-value in compound-treatment and control less than 0.05, limma-p-value for differential expression less than 0.05 and ratio less than 0.75.

### Gene ontology and pathway analysis

Based on the transcriptome analysis, over-represented gene ontology terms and KEGG (Kyoto Encyclopedia of Genes and Genomes) pathways [33] were determined. The GOstats package [34] was used for over-representation analysis of GO terms and the hypergeometric test provided by the R base package was used for over-representation analysis of KEGG pathways. KEGG pathway annotation had been downloaded from the KEGG database in March 2018. *P*-values were adjusted by the q-value method by Storey et al. [35]. Visualization of significant genes in KEGG pathway charts was achieved with the R/Bioconductor pathView package [36].

### Transcription factor analysis

We identified transcription factors (TFs) via motif enrichment analysis in the differentially expressed genes employing the CentriMo (version 5.1.0) tool from the MEME suite [37]. Briefly, we downloaded upstream DNA sequences (2000 bases) of all genes from UCSC GRCh38/hg38 in fasta format. We extracted the first 300 bases upstream of the transcription start site as recommended in the CentriMo manual. Sequences of up- and down regulated genes in the treatments V1, V3 and V5 compared to control were submitted to the tool CentriMO from the MEME suite, using default parameters except for using “anywhere” as kind of local motif enrichment to search for (command: centrimo --oc centrimo_out --verbosity 2 --dfile description --local --score 5.0 --ethresh 10.0 down_hepg2v1_up300_fasta.txt motif_databases/EUKARYOTE/jolma2013.meme motif_databases/JASPAR/JASPAR2018_CORE_vertebrates_non-redundant.meme motif_databases/MOUSE/uniprobe_mouse.meme).

### Construction of protein interaction networks

Based on the Venn diagram analysis, a protein interaction network was constructed using the set of 26 genes that were expressed in common in all treatments (V1, V3 and V5) but not in the untreated HepG2 cells. Interactions annotated with the taxonomy id 9606 (*Homo sapiens*) were filtered from the Biogrid database version 3.4.161 [38]. From this dataset, all protein interactions containing at least one protein coded by the above-mentioned set of 26 genes were extracted. As this network was already too complex, it was reduced by adding only the *n* = 30 interacting proteins with the most interactions to proteins coded by genes from the original set. These interactions were plotted employing the R package network [39], marking proteins from the original set in green. An in-betweenness clustering analysis was performed via the method cluster_edge_betweenness() from the R package igraph [40] in order to identify communities of related proteins within the network.

## Supplementary information

**Additional file 1:.** MEF 134 Sample Analysis by Mass Spectrometry.

**Additional file 2: Table S1.** Venn diagram HepG2 V1 vs. HepG2 control sets, (following tables provided as sheets in the excel file) **Table S2.** Venn diagram HepG2 V3 vs. HepG2 control sets **Table S3:.** Venn diagram HepG2 V5 vs. HepG2 control sets **Table S4.** Venn diagram HepG2 V1, HepG2 V3, HepG2 V5 and HepG2 control sets **Table S5.** Overrepresented KEGG pathways in the down-regulated genes between HepG2 V1 vs. HepG2 control sets **Table S6.** Overrepresented KEGG pathways in the up-regulated genes between HepG2 V1 vs. HepG2 control set **Table S7.** Overrepresented KEGG pathways in the down-regulated genes between HepG2 V3 vs. HepG2 control sets **Table S8.** Overrepresented KEGG pathways in the up-regulated genes between HepG2 V3 vs. HepG2 control sets **Table S9.** Overrepresented KEGG pathways in the down-regulated genes between HepG2 V5 vs. HepG2 control sets **Table S10.** Overrepresented KEGG pathways in the up-regulated genes between HepG2 V5 vs. HepG2 control sets **Table S11.** Overrepresented gene ontologies in the subset of 26 genes expressed in HepG2 V1, V3 and V5 but not in HepG2 control from the venn diagram in Fig. 7d **Table S12.** Detailed listing and functional annotation via the DAVID functional annotation of the subset of 26 genes expressed in HepG2 V1, V3 and V5 but not in HepG2 control from the venn diagram in Fig. 7d **Table S13.** Results of CentriMo (MEME suite) motif enrichment analysis of the 300 bp upstream of the transcription start site for the genes down-regulated in HEPG2 treated with V1 vs. untreated HEPG2. **Table S14.** Results of CentriMo (MEME suite) motif enrichment analysis of the 300 bp upstream of the transcription start site for the genes down-regulated in HEPG2 treated with V3 vs. untreated HEPG2. **Table S15.** Results of CentriMo (MEME suite) motif enrichment analysis of the 300 bp upstream of the transcription start site for the genes down-regulated in HEPG2 treated with V5 vs. untreated HEPG2. **Table S16.** Results of CentriMo (MEME suite) motif enrichment analysis of the 300 bp upstream of the transcription start site for the genes up-regulated in HEPG2 treated with V1 vs. untreated HEPG2. **Table S17.** Results of CentriMo (MEME suite) motif enrichment analysis of the 300 bp upstream of the transcription start site for the genes up-regulated in HEPG2 treated with V3 vs. untreated HEPG2. **Table S18.** Results of CentriMo (MEME suite) motif enrichment analysis of the 300 bp upstream of the transcription start site for the genes up-regulated in HEPG2 treated with V5 vs. untreated HEPG2. **Table S19.** Summary of the 20 most significant results of all CentriMo (MEME suite) motif enrichment analyses.

**Additional file 3: Figure S1.** KEGG pathway chart of pathway Steroid Biosynthesis in genes down-regulated in HepG2 cells treated with V1 vs. control.

**Additional file 4: Figure S2.** KEGG pathway chart of pathway Drug metabolism – cytochrome P450 in genes down-regulated in HepG2 cells treated with V1 vs. control.

**Additional file 5: Figure S3.** KEGG pathway chart of pathway p53 signaling pathway in genes down-regulated in HepG2 cells treated with V1 vs. control.

**Additional file 6: Figure S4.** KEGG pathway chart of pathway cell cycle in genes down-regulated in HepG2 cells treated with V3 vs. control.

**Additional file 7: Figure S5.** KEGG pathway chart of pathway viral carcinogenesis in genes up-regulated in HepG2 cells treated with V3 vs. control.

**Additional file 8: Figure S6.** Fig: KEGG pathway chart of pathway cell cycle in genes up-regulated in HepG2 cells treated with V5 vs. control.

**Additional file 9: Figure S7.** Dendrogram of community clustering of protein interaction networks of HepG2 cells treated with V1, V3 and V5.

**Additional file 10: Figure S8.** Pairwise scatter plots of logarithmic (base 2) expression values of all samples of HepG2 cells treated with V1, V3, V5 and untreated versus each other.

## Data Availability

The datasets supporting the conclusions of this article are included within the article (and its additional files).

## Abbreviations

ADH4: Alcohol dehydrogenase 4
ALDH3B: Aldehyde dehydrogenase 3B
APOBR: Apolipoprotein B receptor
APP: Amyloid precursor protein
BEGAIN: Brain enriched guanylate kinase associated
CDKN1B: Cyclin dependent kinase inhibitor 1B
CDX1: Caudal type homeobox 1
DCM: Dichloromethane
DMRT1: Doublesex and mab-3 related transcription factor 1
DNA: Deoxyribonucleic acid
EPHA8: EPH receptor A8
FB: Butanol fraction
FD: Dichloromethane fractions
FD-K1: Dichloromethane fractions obtained from the first round of the Kupchan fractionation
FE: Ethyl acetate fraction
FH: Hexane fraction
FME: Methanol & ethyl acetate fraction
FM50: 50% methanol fraction
FW: Water fraction
GO: Gene ontology
GPR137B: G protein-coupled receptor 137B
GSTA1: Glutathione S-transferase alpha 1
HepG2: Name of a human liver cancer cell line
HIF: Hypoxia inducible factor 1
HOX: Homeobox
HPLC-HRMS: High performance liquid chromatography coupled with high-resolution mass spectrometry
KEGG: Kyoto Encyclopedia of genes and genomes
KLF: Kruppel like factor
LPAR3: Lysophosphatidic acid receptor 3
MAPK: Mitogen-activated protein kinase
MEF: Marine endophytic fungi
mRNA: Messenger ribonucleic acid
NPY4R: Neuropeptide Y receptor Y4
OD600: Optical density at 600 nm
PAR4: Pro-apoptotic WT1 regulator
POU3F1: POU class 3 homeobox 1
PRR9: Proline rich 9
P/S: Penicillin/Streptomycin
qRT-PCR: Quantitative real time PCR
RMA: Robust Multi-array Average
RT: Reverse Transcription
SOX: SRY-box transcription factor
SP1: Sp1 transcription factor
TBP: TATA-box binding protein
TF: Transcription factor
TGFB: Transforming growth factor beta
TLC: Thin layer chromatography
TNF: Tumor necrosis factor
TREML2: Triggering receptor expressed on myeloid cells like 2
TRIM21: Tripartite motif containing 21
ZIC4: Zic family member 4
ZNF619: Zinc finger protein 619
ZSCAN20: Zinc finger and SCAN domain containing 20
