# Supplementary material for: Transcriptomic analysis of marine endophytic fungi extract identifies highly enriched anti-fungal fractions targeting cancer pathways in HepG2 cell lines
Source: BMC Genomics. 2020 Mar 30;21:265. doi: 10.1186/s12864-020-6684-z (PMC7106652; doi:10.1186/s12864-020-6684-z)

MEF 134 Sample Analysis by Mass Spectrometry

1. MEF 134 Crude Extract **(Pages 5-17)**
2. MEF 134 FD fraction (Dichloromethane fraction of crude) **(Pages 18-35)**
3. MEF 134 FD-V5 (1st TLC fraction 5) **(Pages 36-45)**
4. MEF 134 FD-V5V7 (2nd TLC fraction 7 of 1st TLC fraction 5) **(Pages 46-57)**
5. MEF 134 FD-V5V9 (2nd TLC fraction 9 of 1st TLC fraction 5) **(Pages 58-66)**
6. MEF 134 FD K1V1V1 (fraction preparation illustrated in Fig 1) **(Pages 67-77)**
7. MEF 134 FD K1V5V3 (fraction preparation illustrated in Fig 1) **(Pages 78-83)**
8. MEF 134 FD K2V3V5 (fraction preparation illustrated in Fig 1) **(Pages 84-95)**

The HPLC-HRMS full scan was performed on the fractions from MEF 134. These fractions include; MEF 134 crude, MEF 134 FD, MEF 134 FDV5, MEF 134 FDV5V7, MEF134FDV5V9, MEF 134 FD K1V1V1, MEF 134 FD K1V5V3 and MEF 134 FD K2V3V5.

All the major peaks and masses from the absorbance and total ion chromatogram spectra from the crude extract were distinctly different compared to that obtained from the FD sample in terms of the mixture of compounds detected in corresponding peaks. Whilst the crude and the FD had several prominent masses in common, other co-eluting compounds found in the corresponding peak of the crude were absent or minor in the FD. This observation indicated that Kupchan fractionation achieved high enrichment to a large extent. The comparisons between the samples FD and FDV5 showed no observable peaks that were identical which demonstrates a remarkably higher levels of enrichment of bioactive compounds as determined in the bioassays. However,

fractions FDV5V7 and FDV5V9 contain similar compounds, for example molecular weight 354 (M+H) with the sum formula $C_{20}H_{40}O_2N_3$ and a similar compound with one C_2H_4 group more with mass 282 and sum formula $C_{22}H_{44}O_2N_3$ were identified. The two other compounds with mass 253 and sum formula $C_{15}H_{29}ON_2$ and compound with mass 237 and sum formula $C_{15}H_{29}N_2$ were also present in both fractions. There are three compounds with molecular weight above 1000 Da which are clear distinct for FDV5V9, these are 1031 Da, 1045 Da and 1059 Da their bioactivity can be readily determined in future experiments using size exclusion chromatography. This technique was not employed in this study due to the limited quantities of the extracts obtained from the 20 Litres of culture. These interesting observations save us great motivation to develop the capacity to culture and process at a high scale.

The HPLC-HRMS full scan of MEF134 FD K1V1V1 showed 11 major components. The mass spectrometry analyses identified some major masses which are 149.02, 337.23, 409.29, 265.26 and 391.30 Da.

The total ion current chromatogram of MEF134 FD K1V5V3 detected 6 major components. Mass spectrometry analyses of the components identified these masses; 340.23, 453.34 and 267.17.

From the total ion current chromatogram of MEF 134 FD K2V3V5, 11 major components were detected. Analysis of these components detected these major masses; 340.26, 425.30, 300.29 and 568.48. The major masses obtained from the 3 anti-cancer fractions were not similar which also indicating that different compounds in these three fractions are responsible for the activities seen.

1. MEF134 Crude
HPLC-HRMS Fullscan

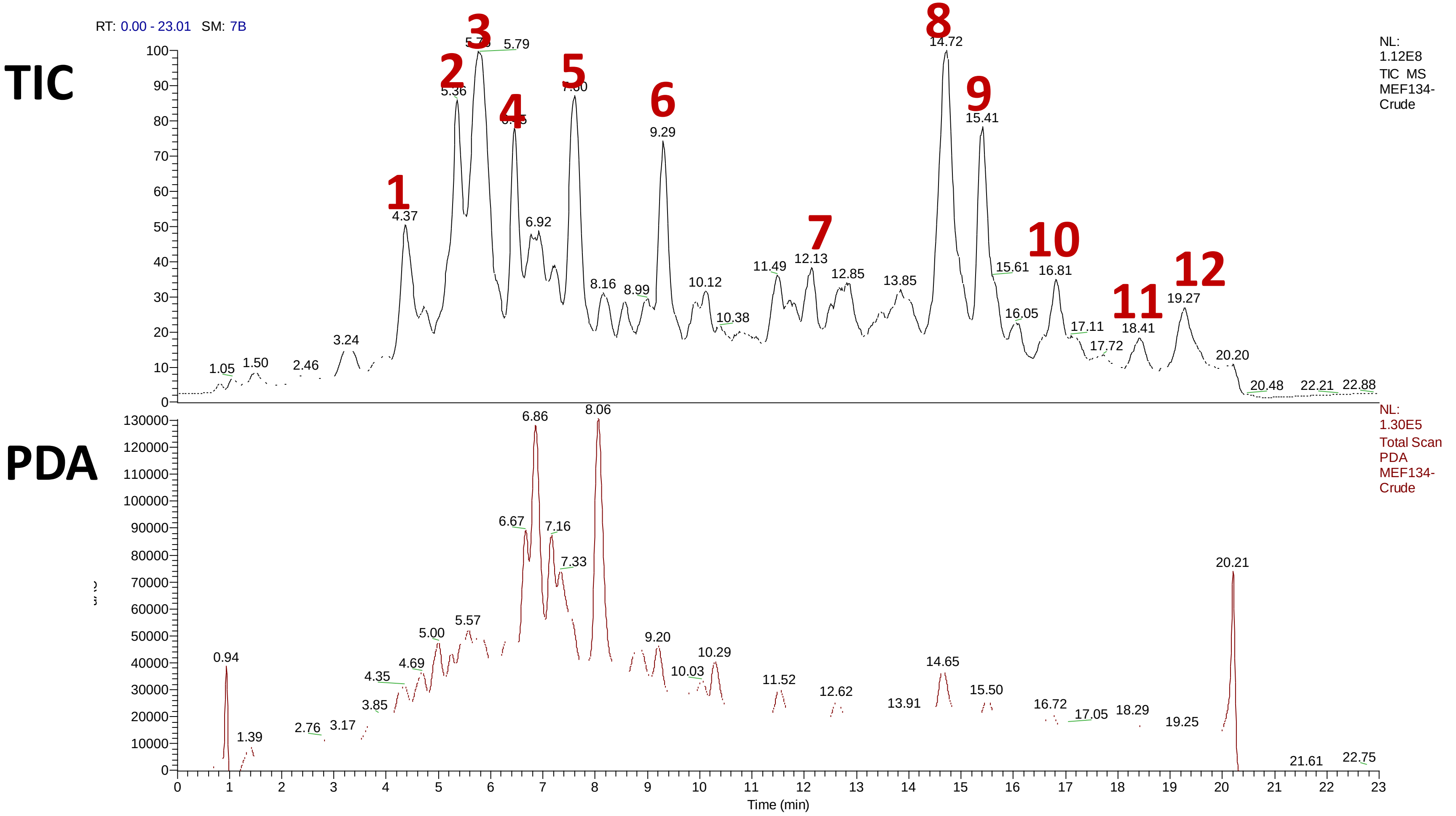

1. MEF134 Crude
HPLC-HRMS Spectra

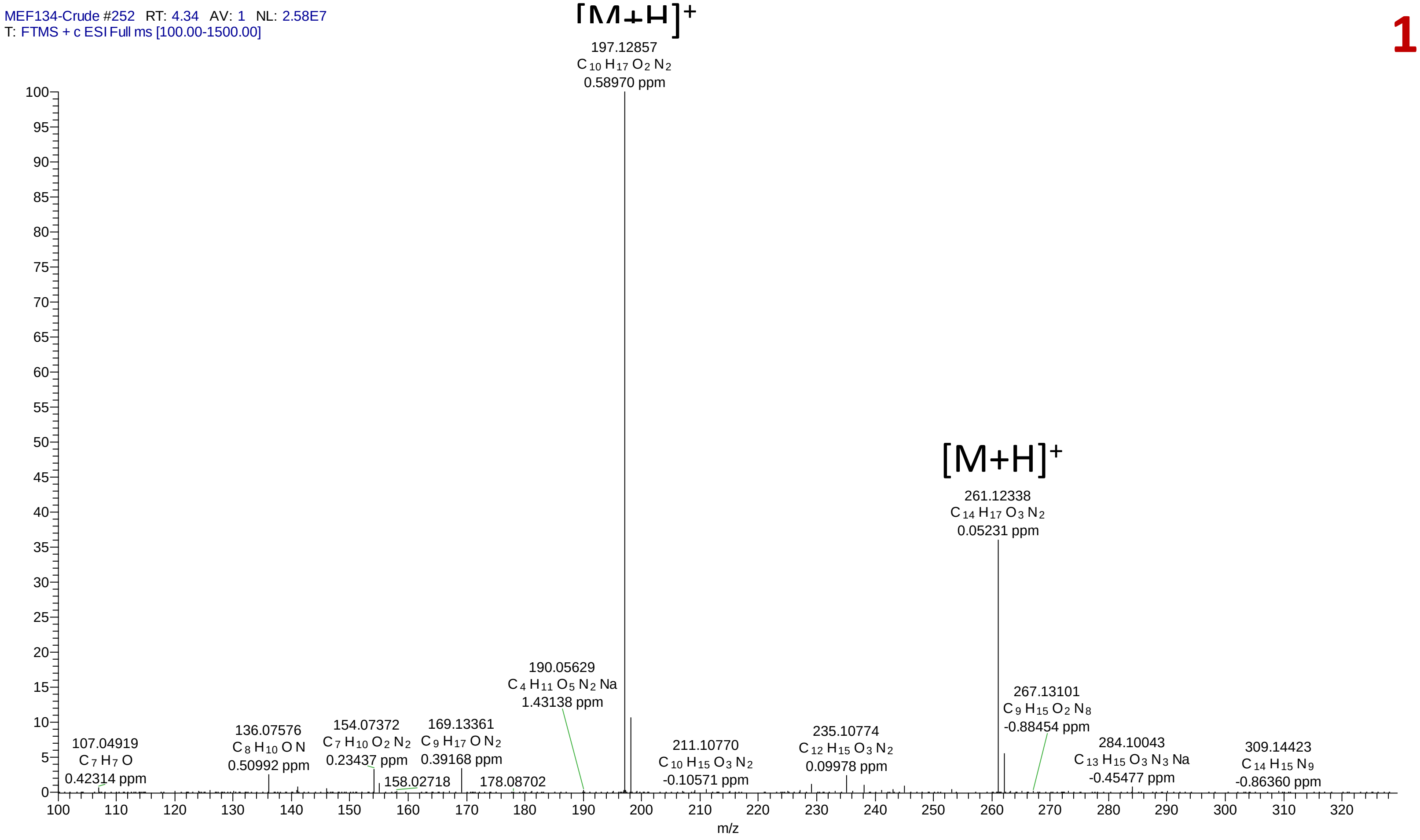

1

1. MEF134 Crude
HPLC-HRMS Spectra

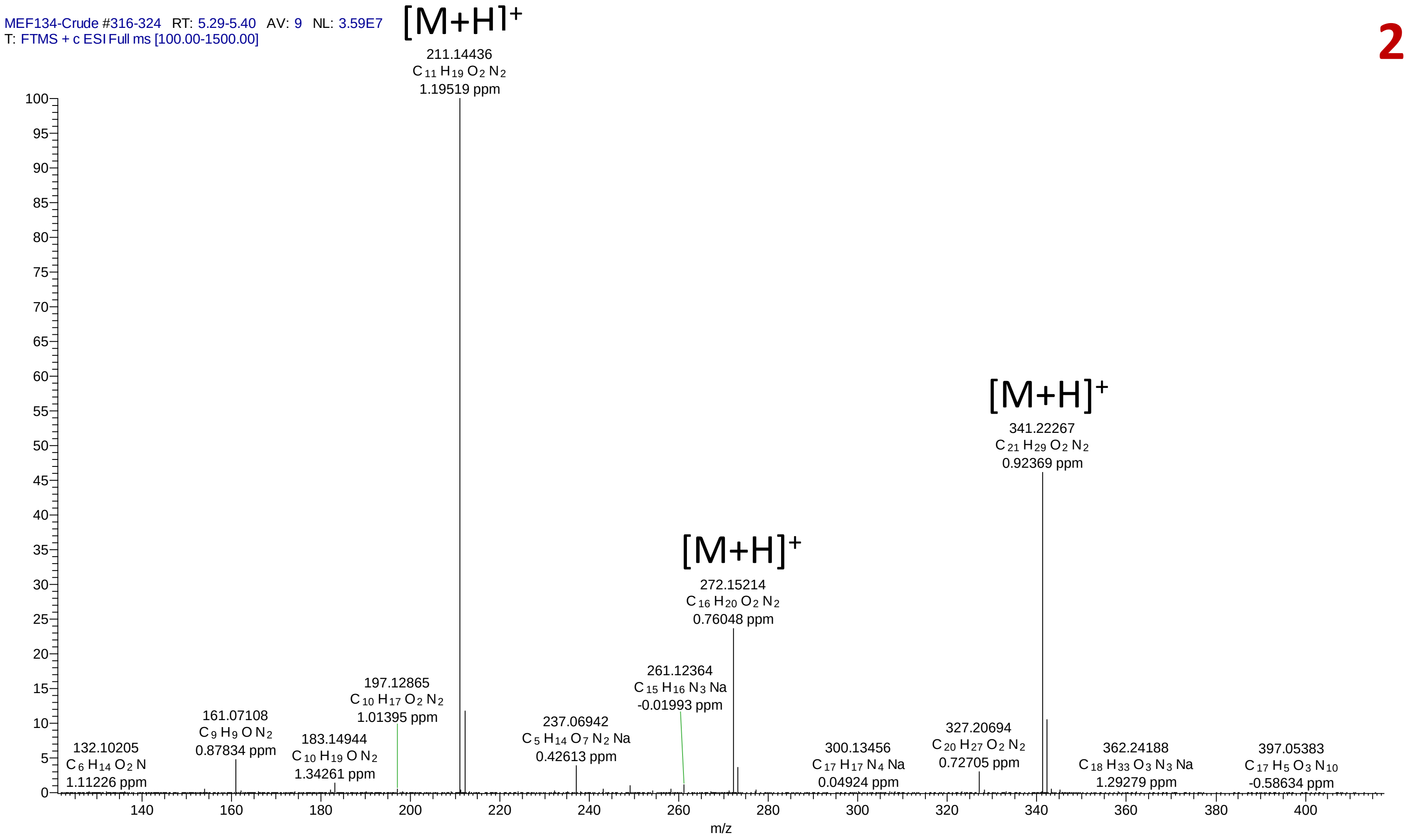

2

1. MEF134 Crude
HPLC-HRMS Spectra

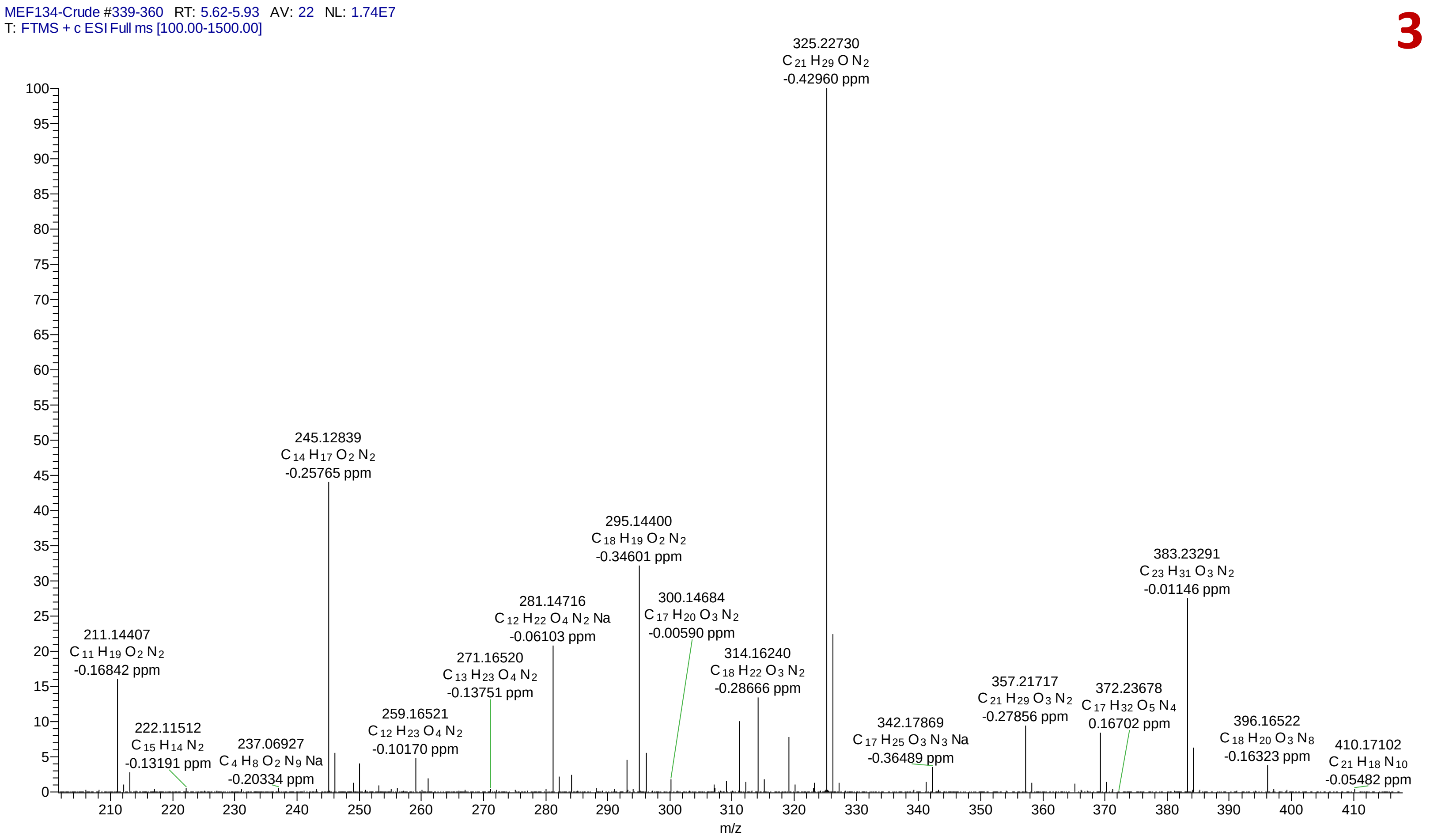

1. MEF134 Crude
HPLC-HRMS Spectra

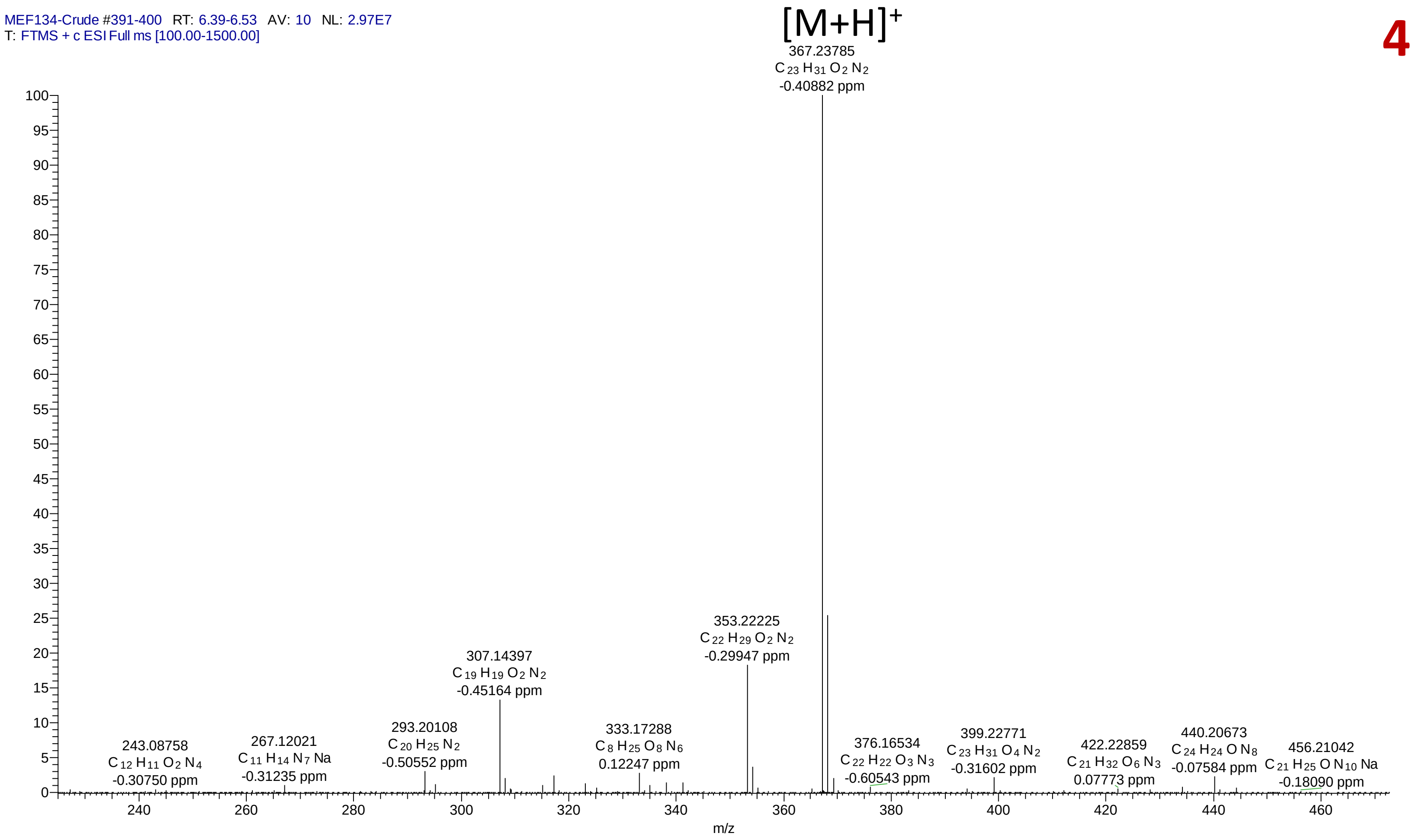

4

1. MEF134 Crude HPLC-HRMS Spectra

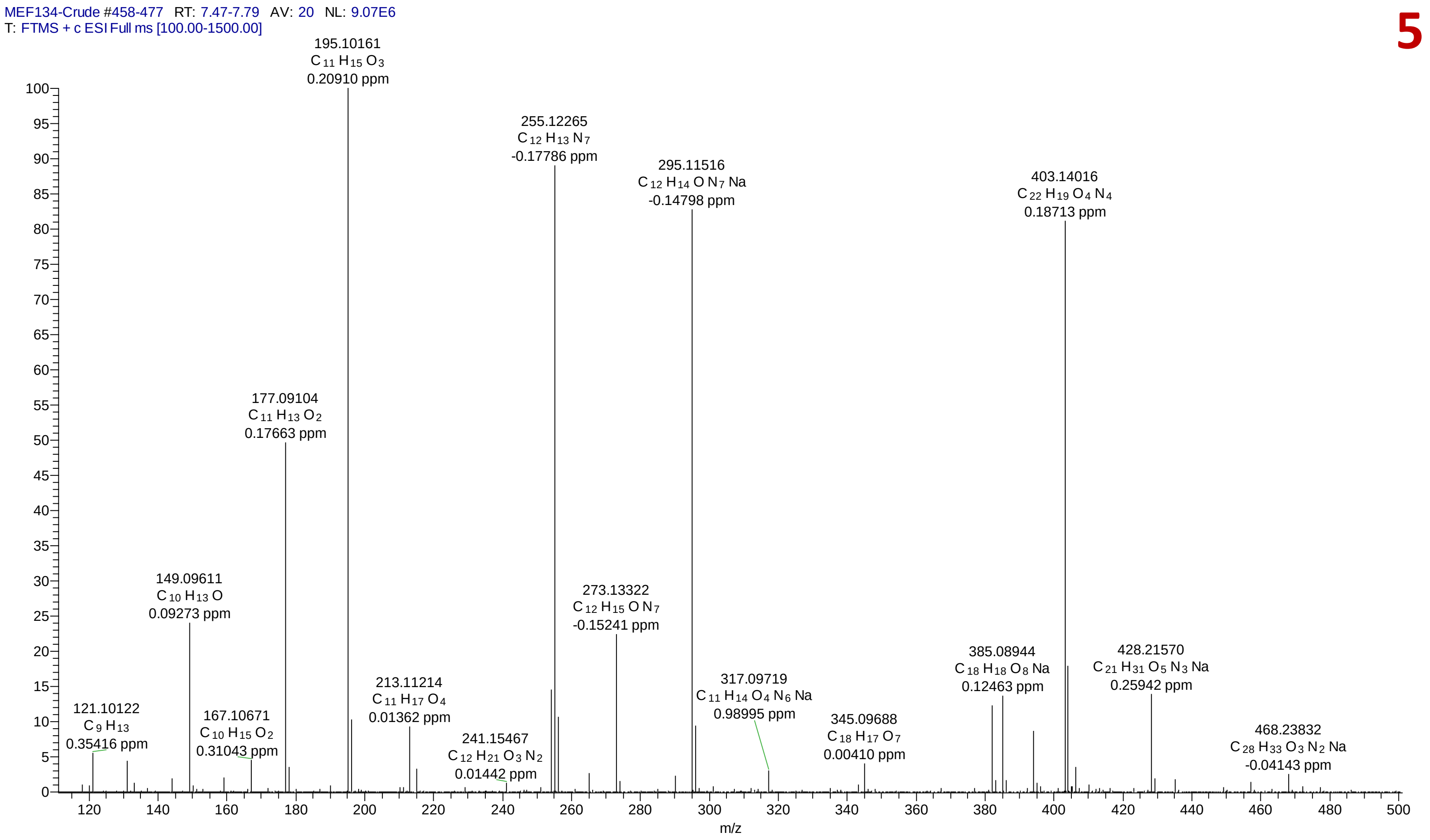

1. MEF Crude
HPLC-HRMS Spectra

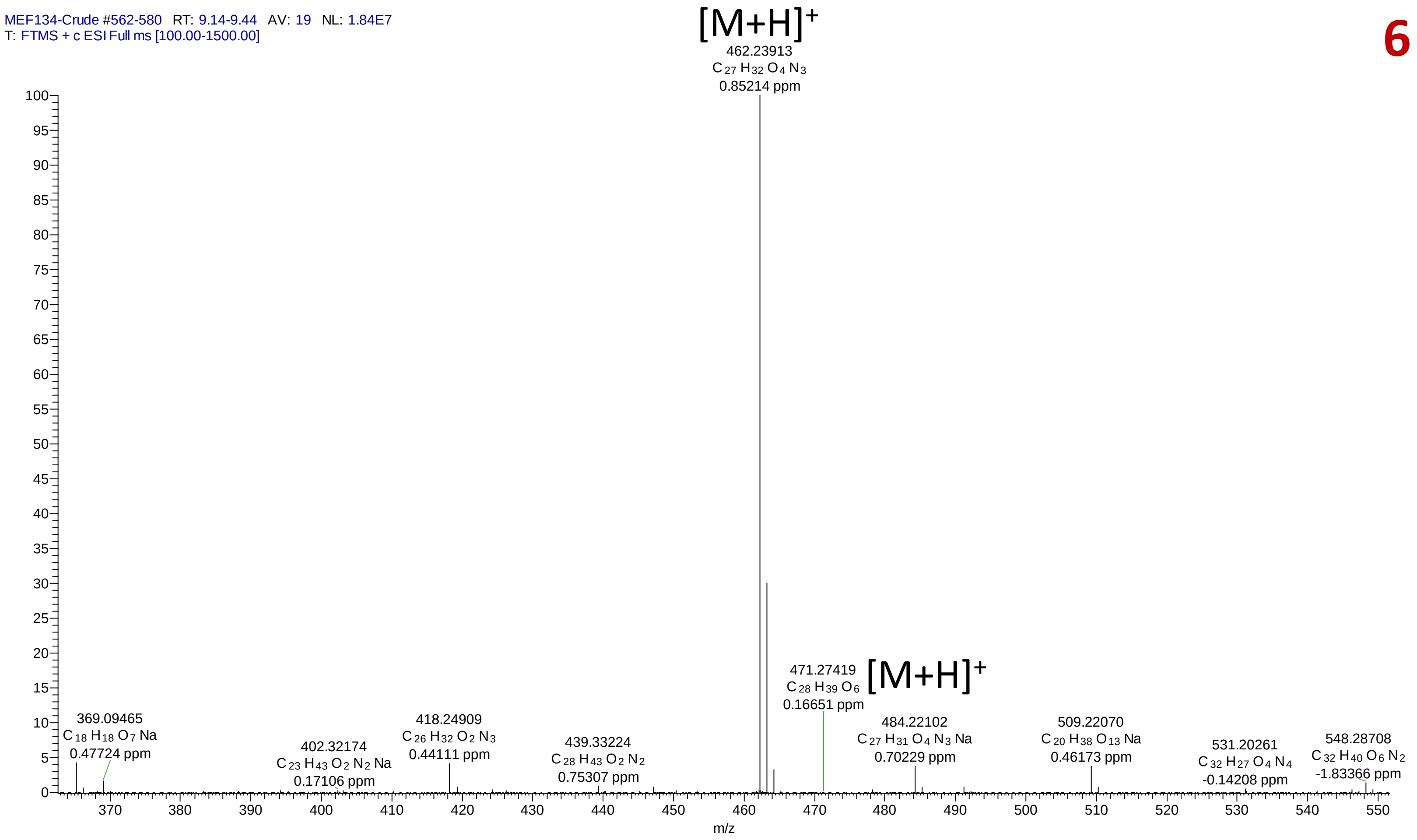

1. MEF134 Crude HPLC-HRMS Spectra

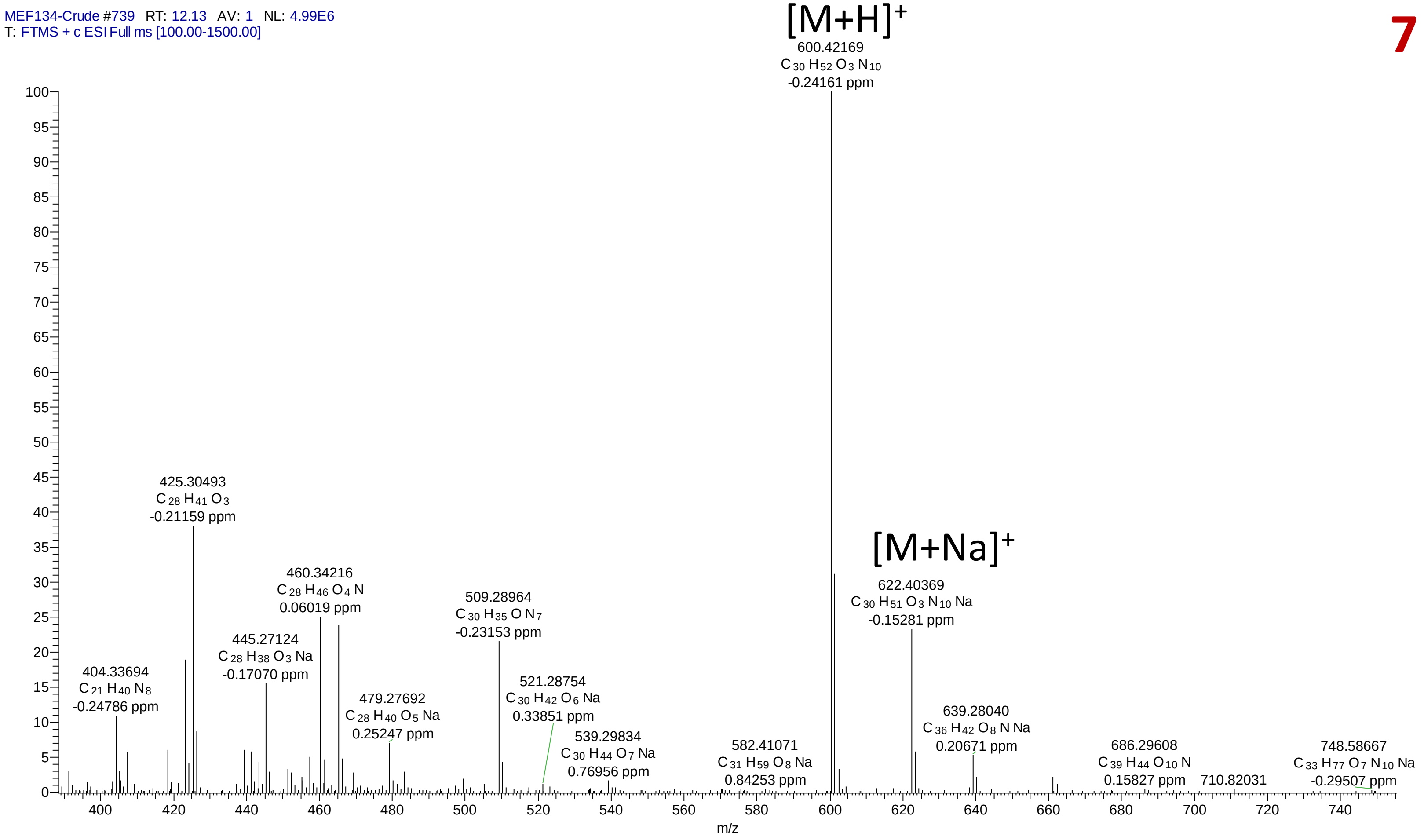

1. MEF134 Crude
HPLC-HRMS Spectra

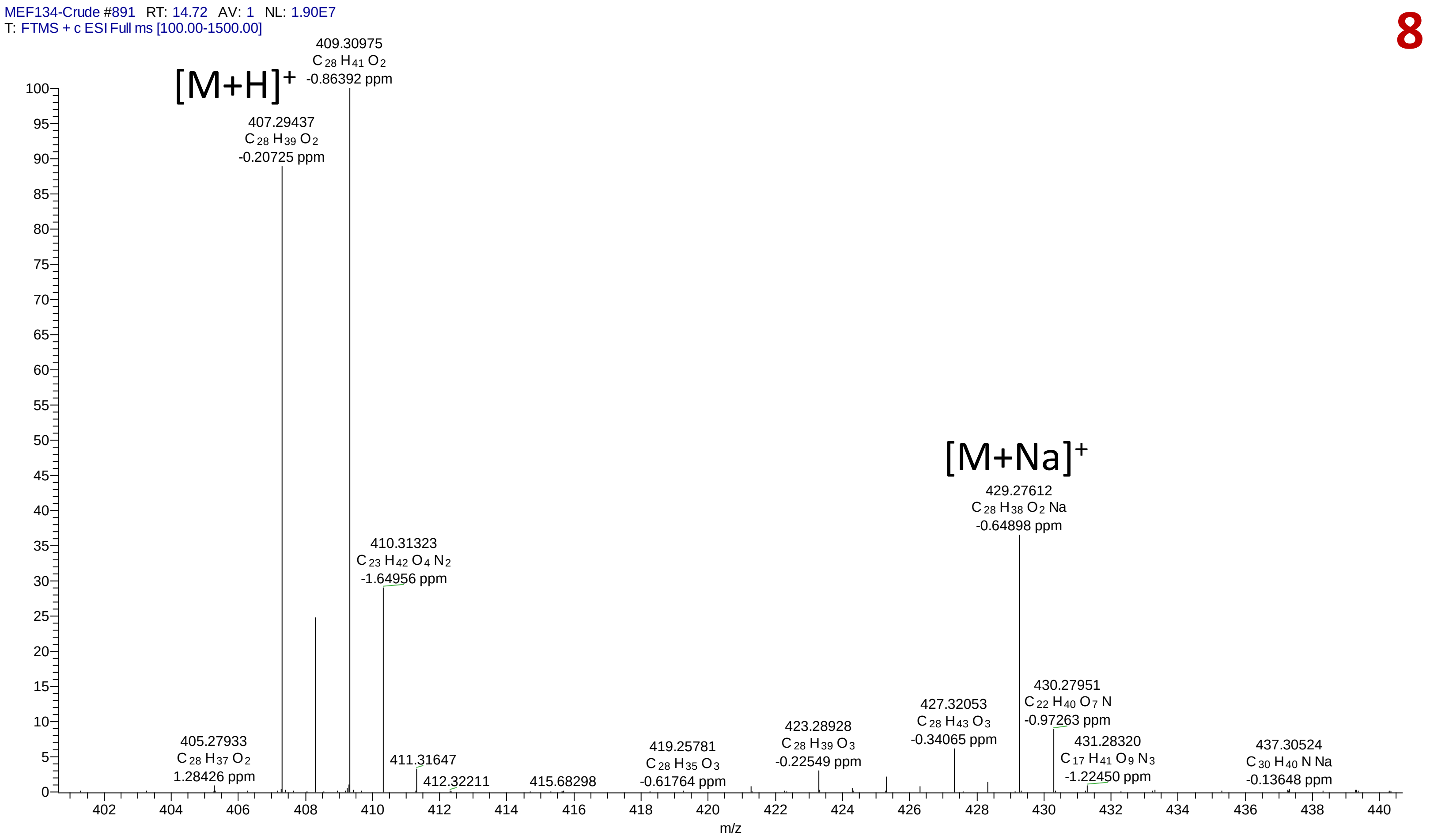

1. MEF134 Crude HPLC-HRMS Spectra

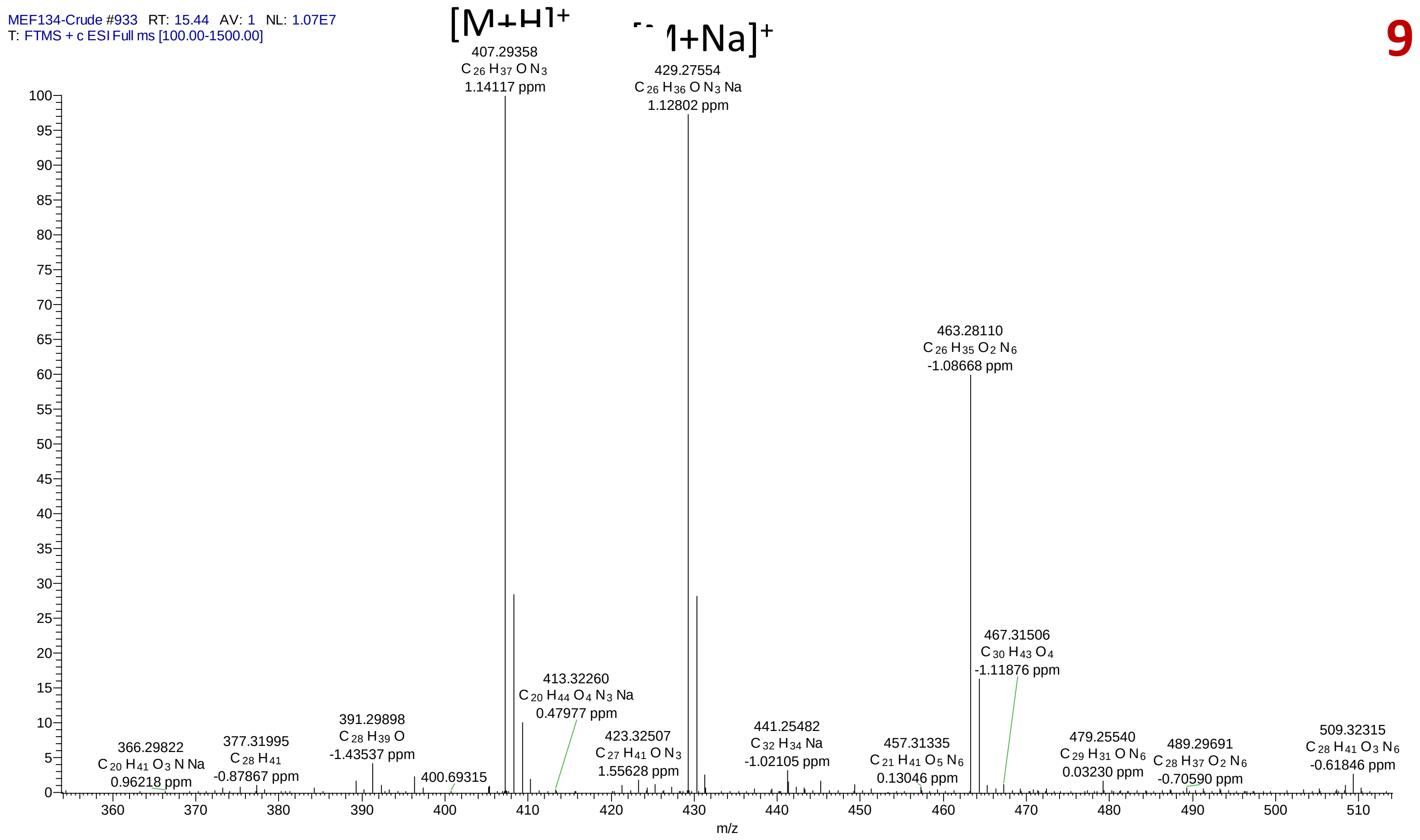

1. MEF134 Crude
HPLC-HRMS Spectra

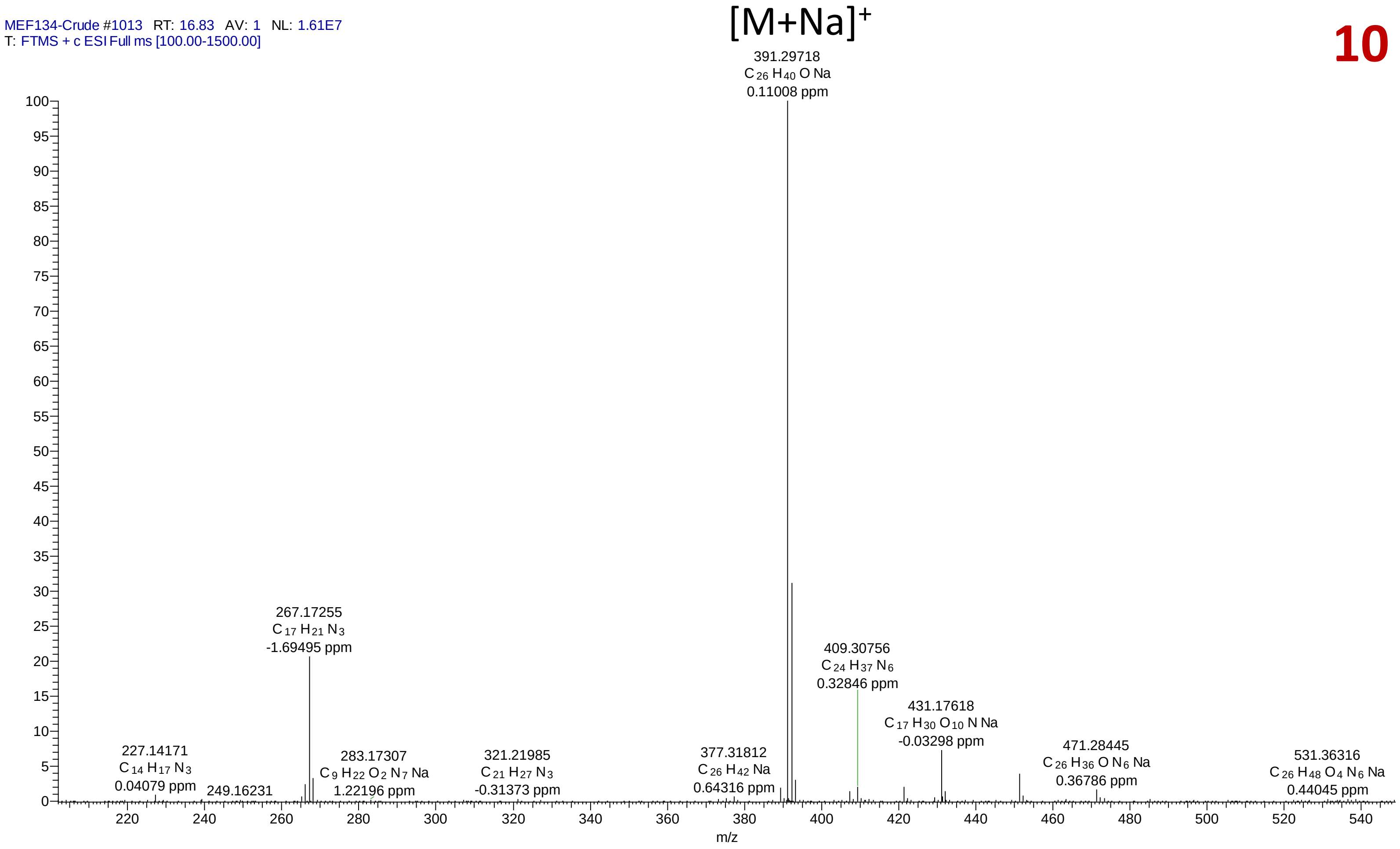

10

1. MEF134 Crude
HPLC-HRMS Spectra

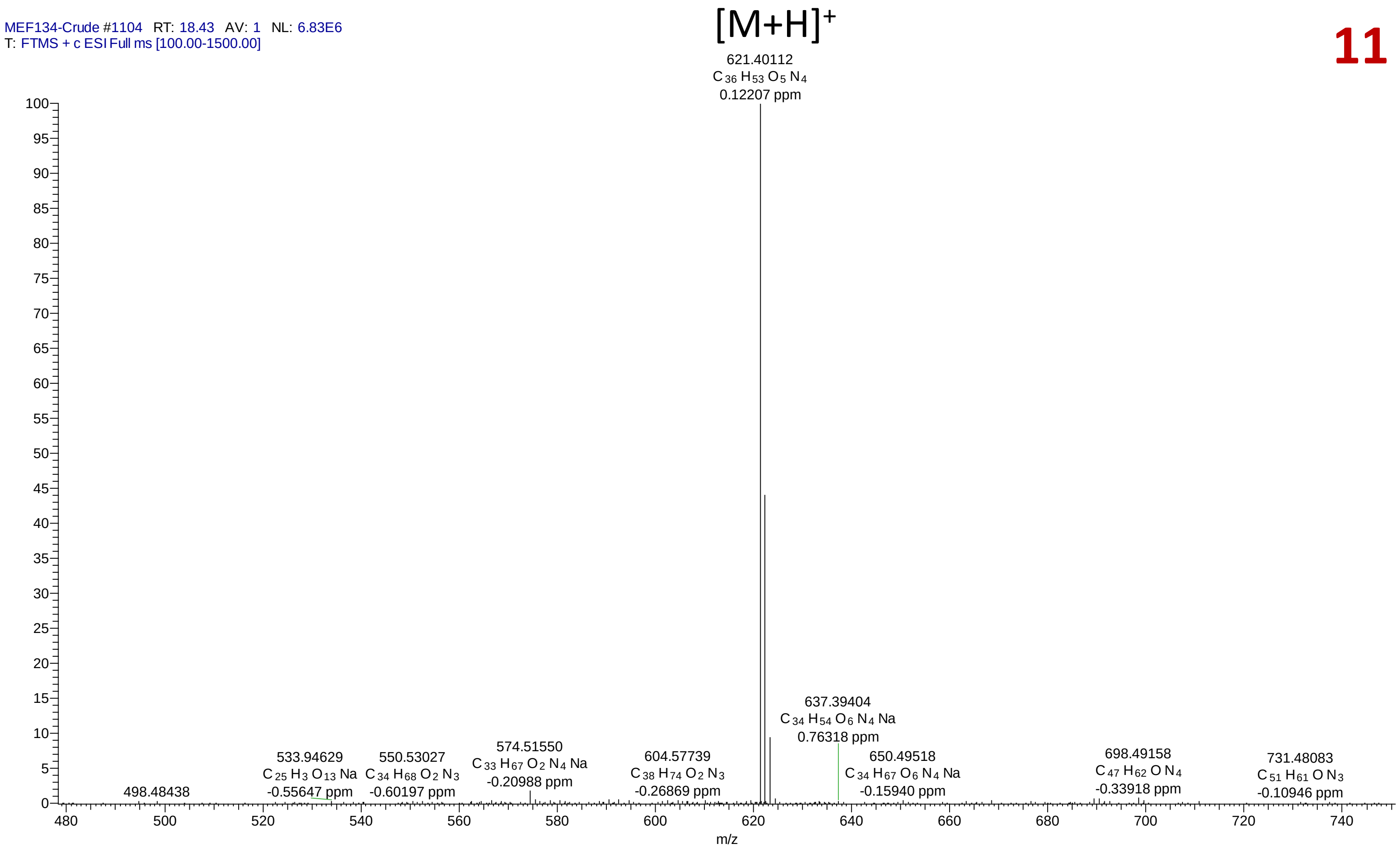

1. MEF134 Crude
HPLC-HRMS Spectra

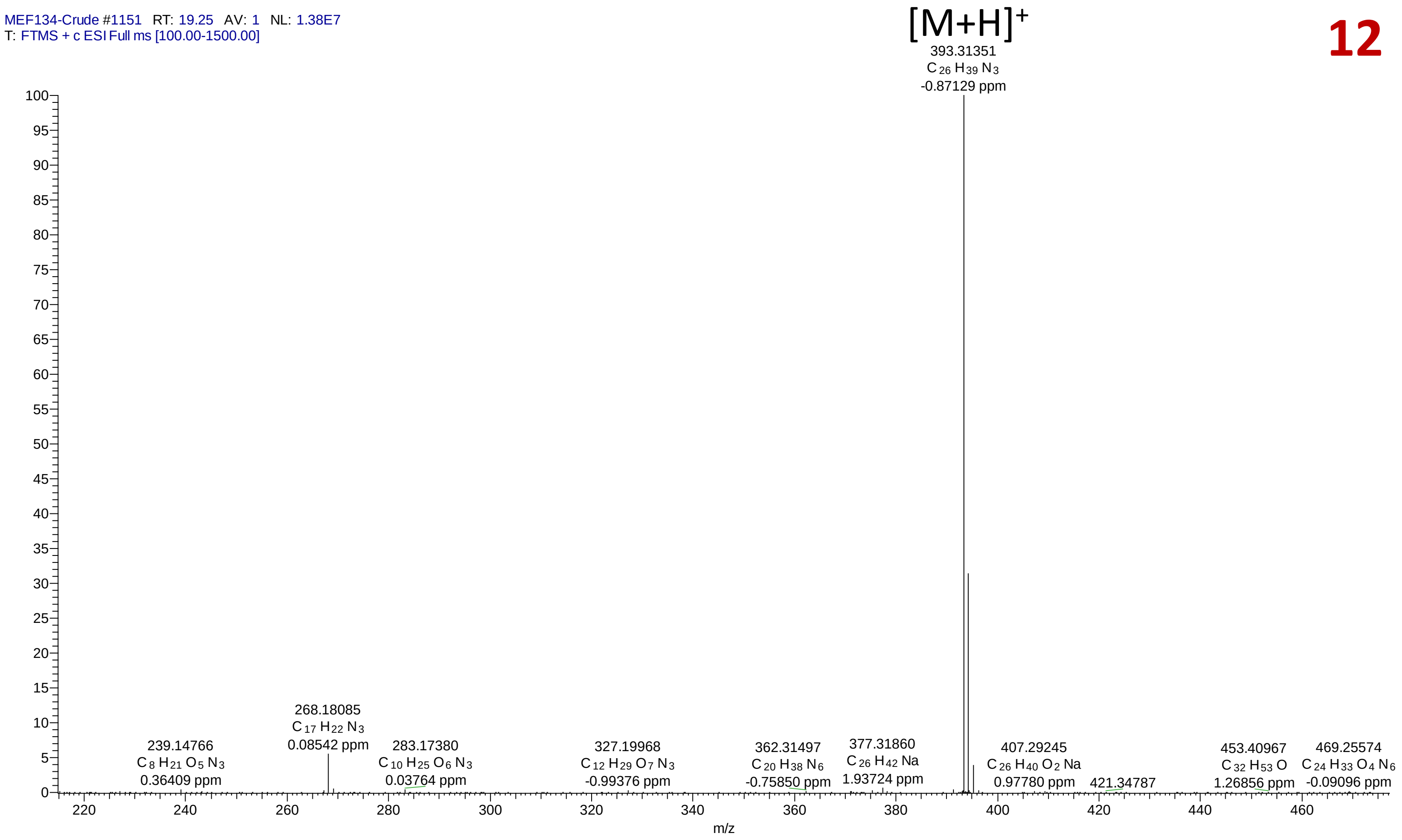

2. MEF134 FD
HPLC-HRMS Fullscan

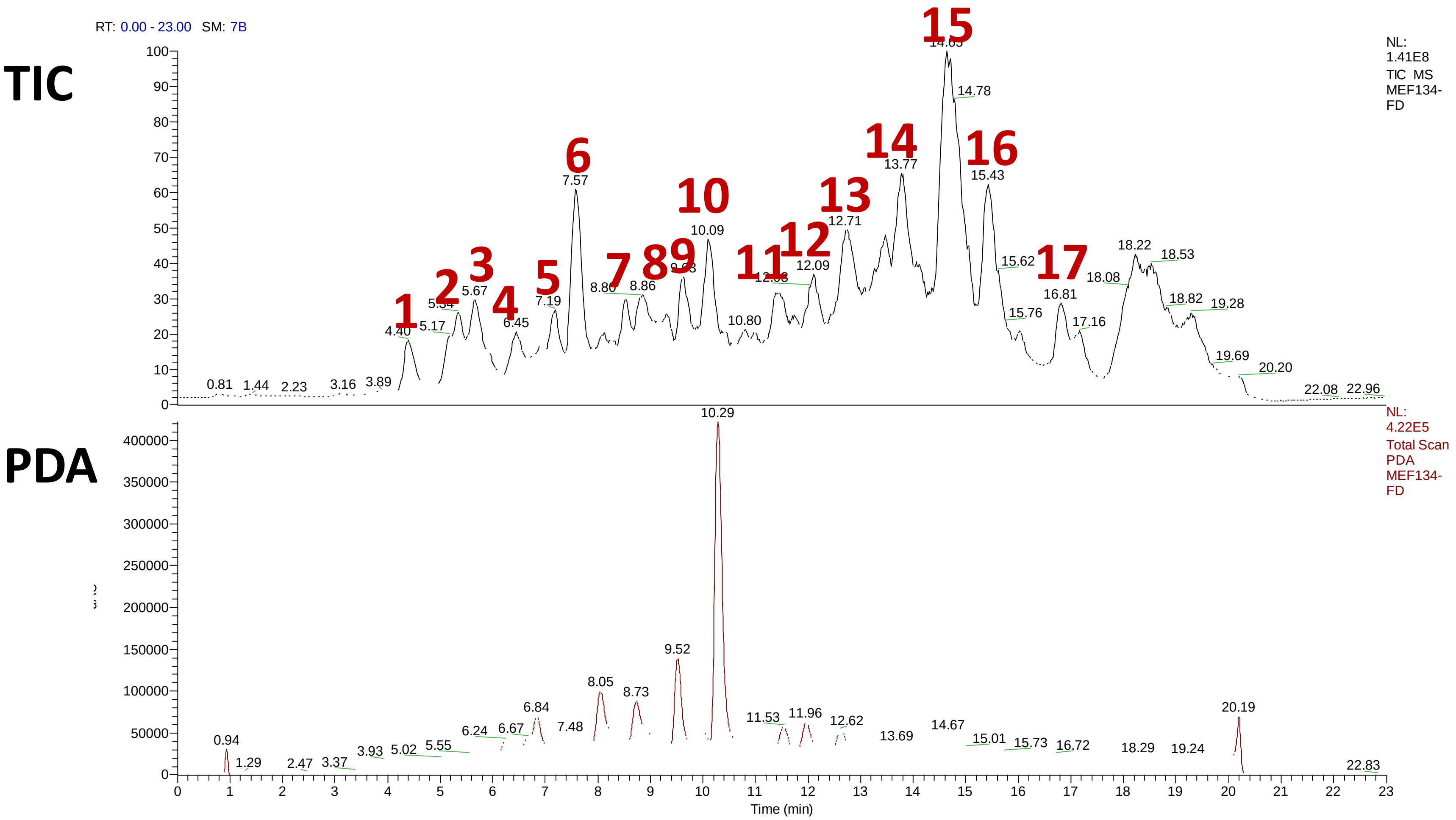

2. MEF134 FD
HPLC-HRMS Spectra

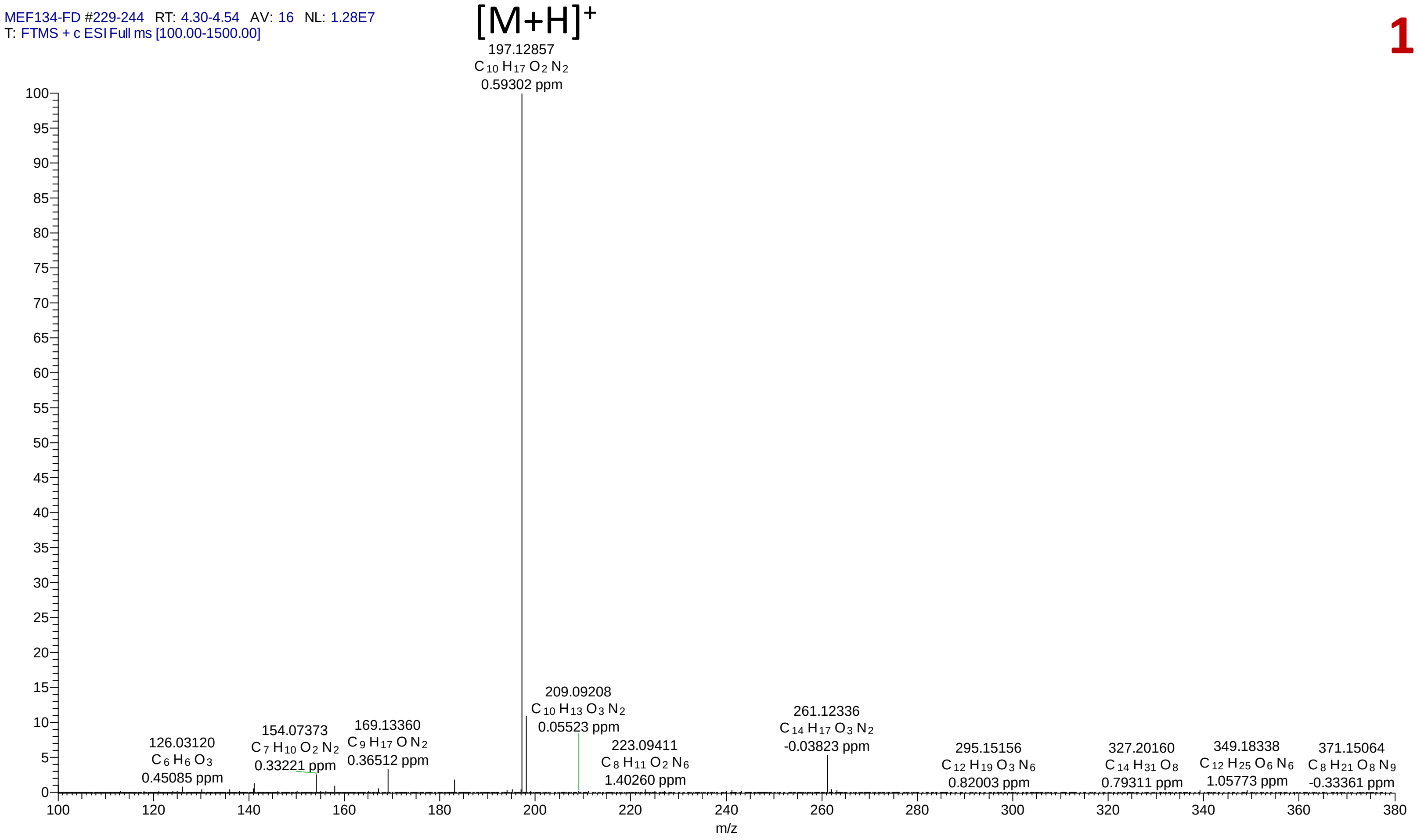

2. MEF134 FD
HPLC-HRMS Spectra

2

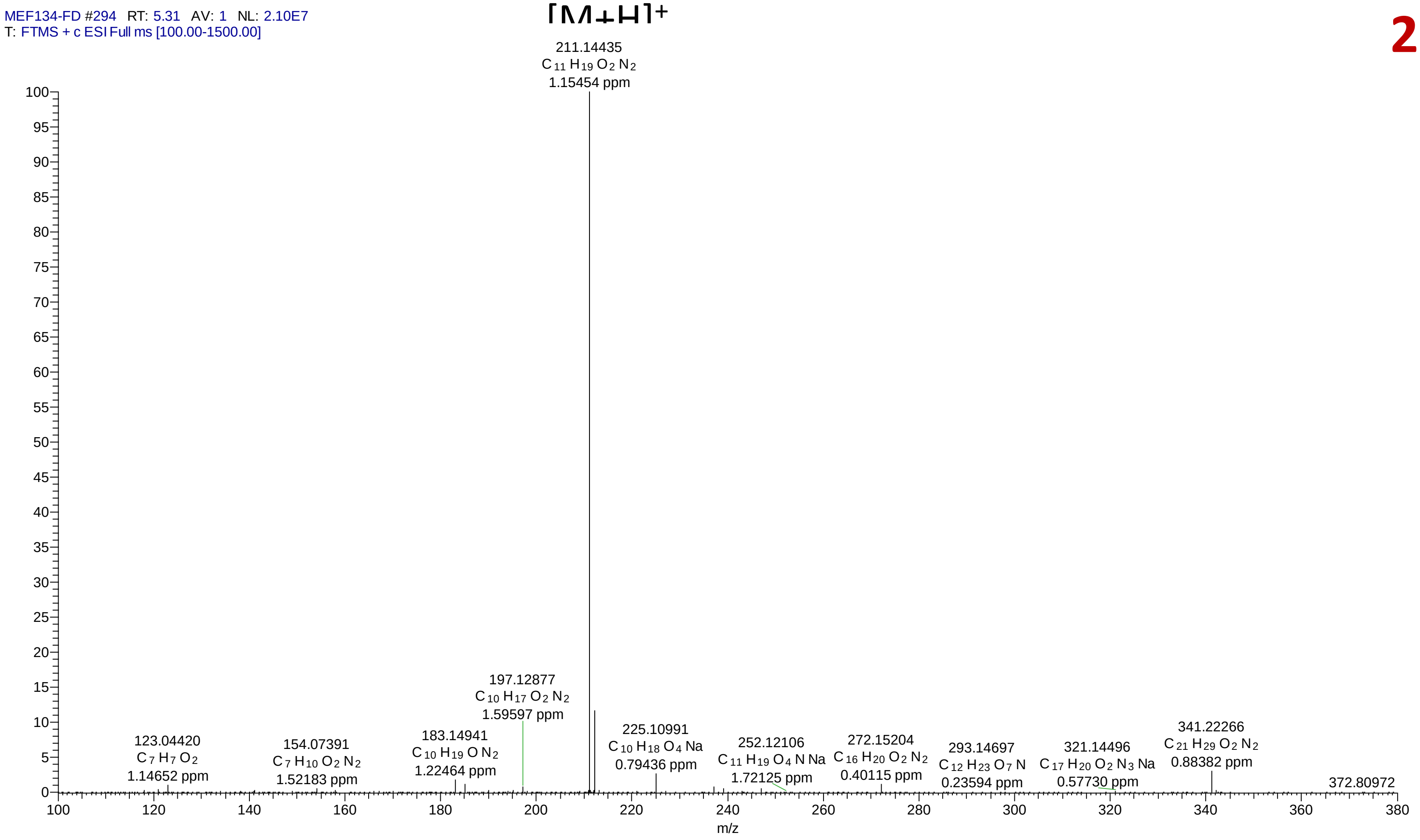

2. MEF134 FD
HPLC-HRMS Spectra

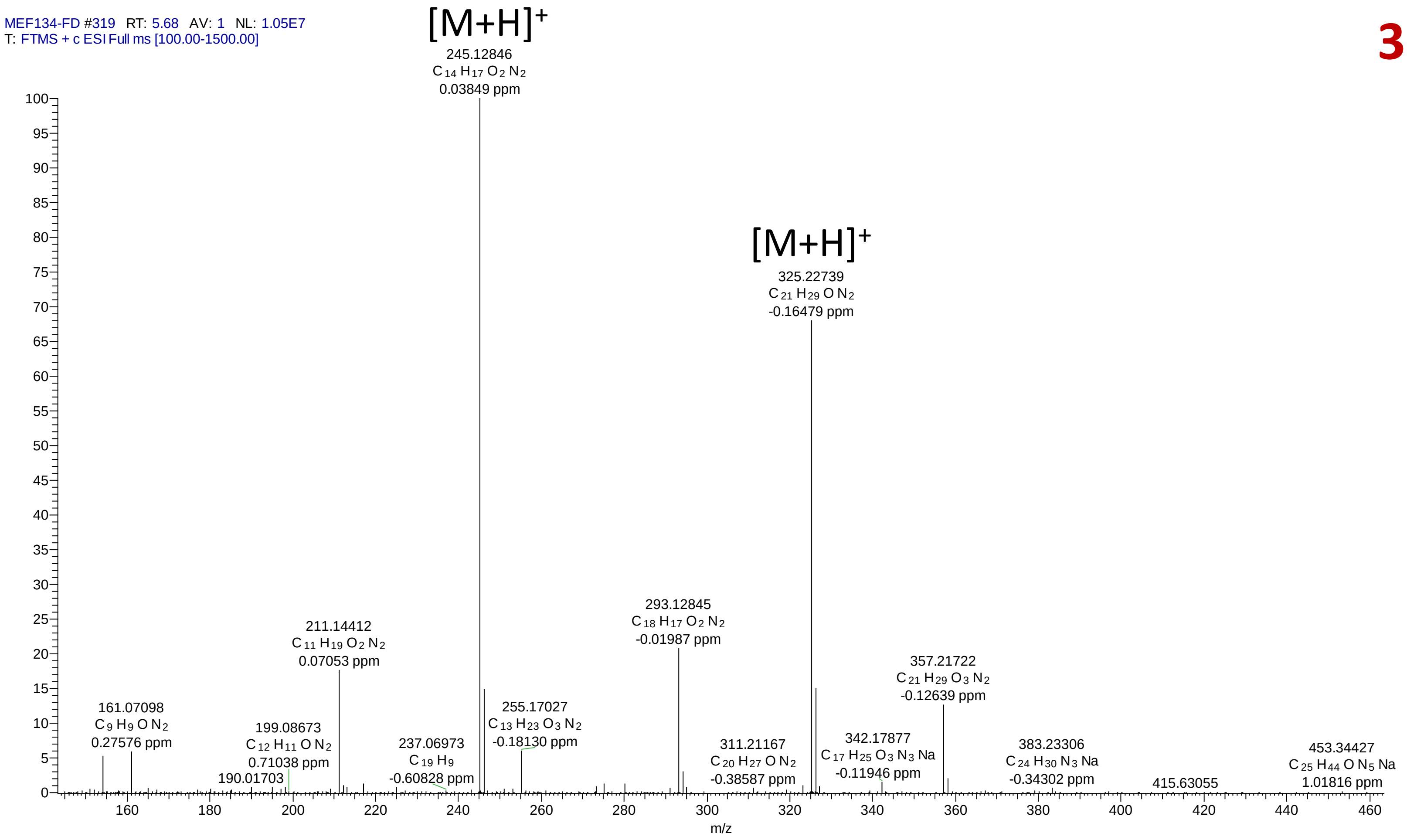

3

2. MEF134 FD
HPLC-HRMS Spectra

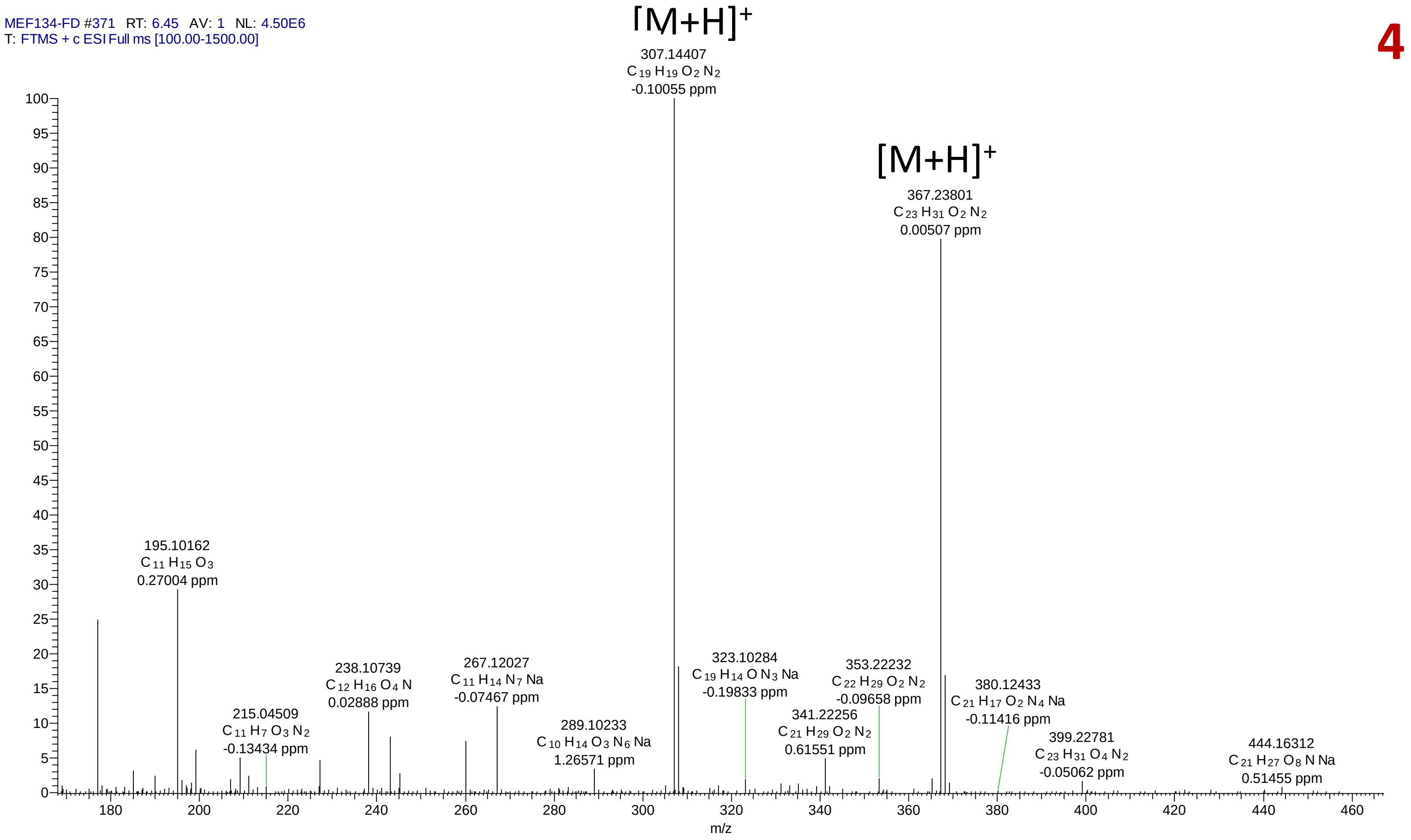

2. MEF134 FD HPLC-HRMS Spectra

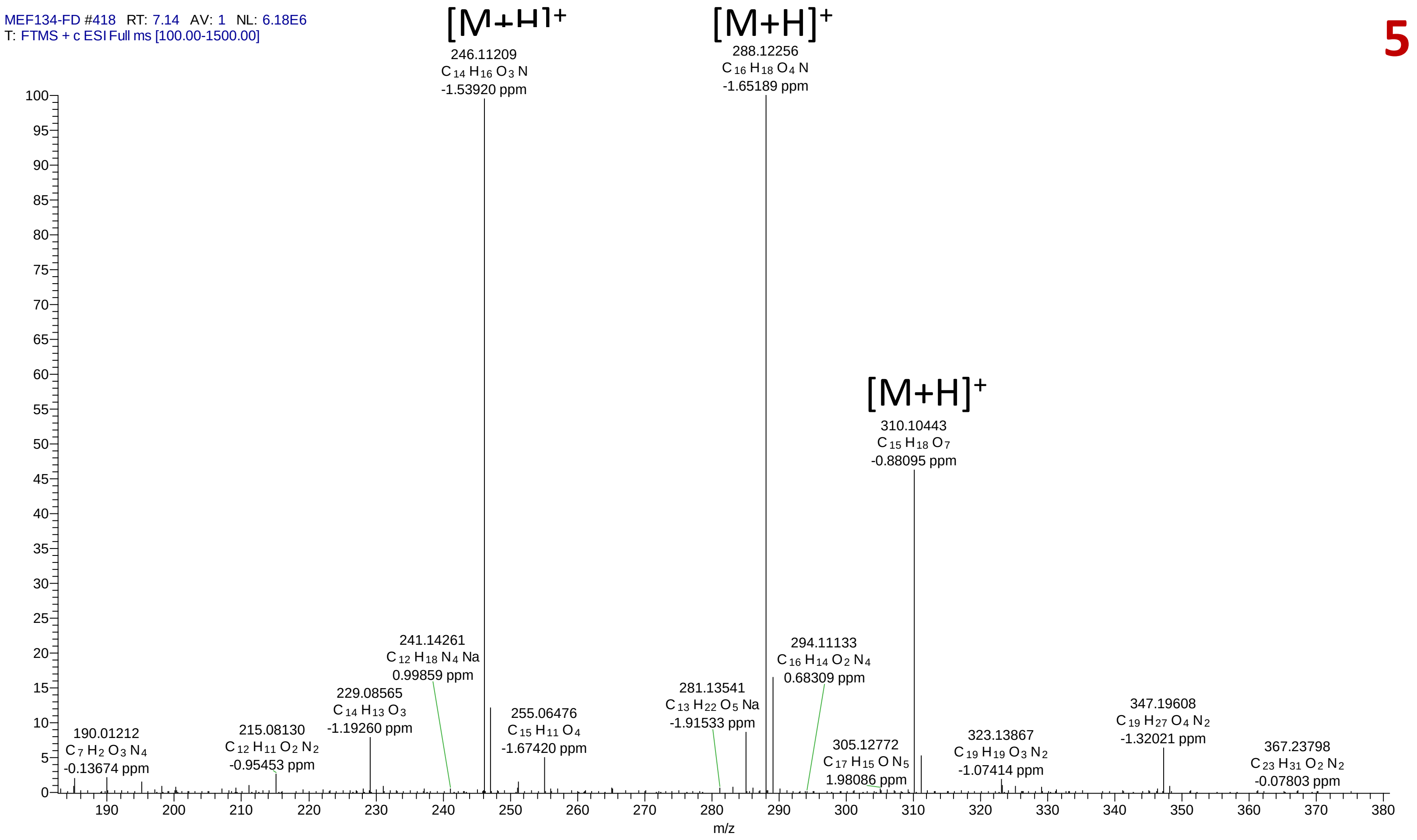

2. MEF134 FD
HPLC-HRMS Spectra

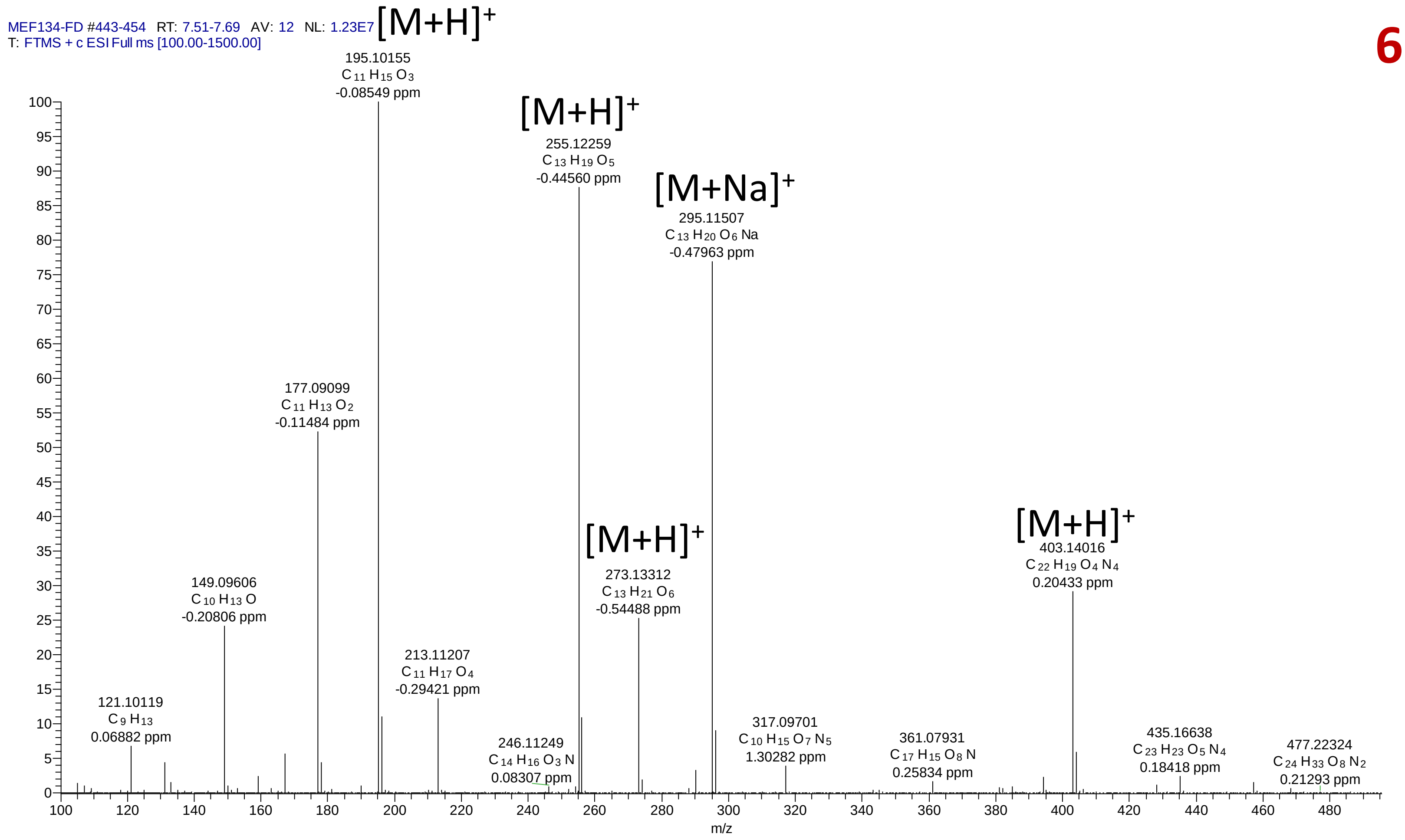

6

2. MEF134 FD
HPLC-HRMS Spectra

7

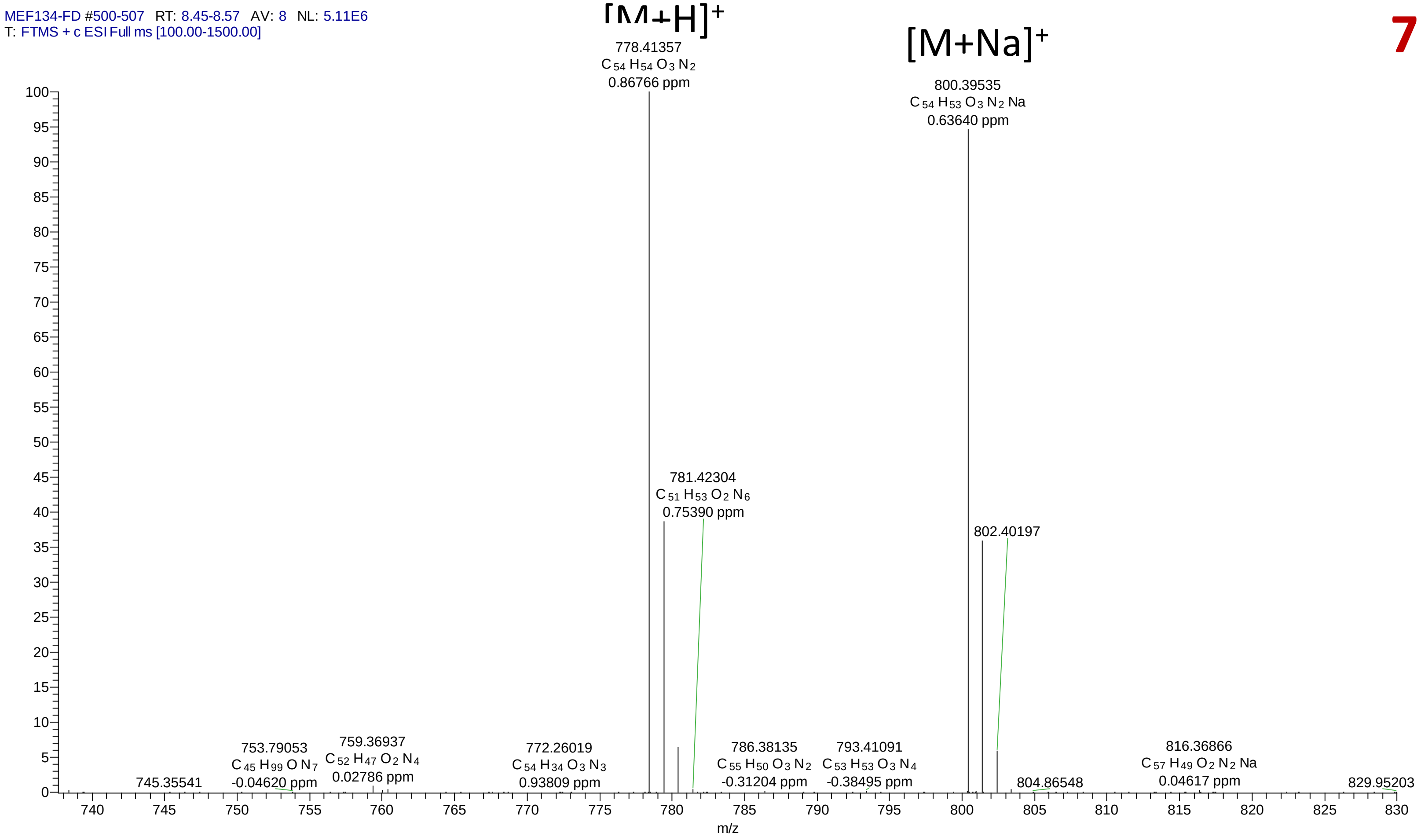

2. MEF134 FD
HPLC-HRMS Spectra

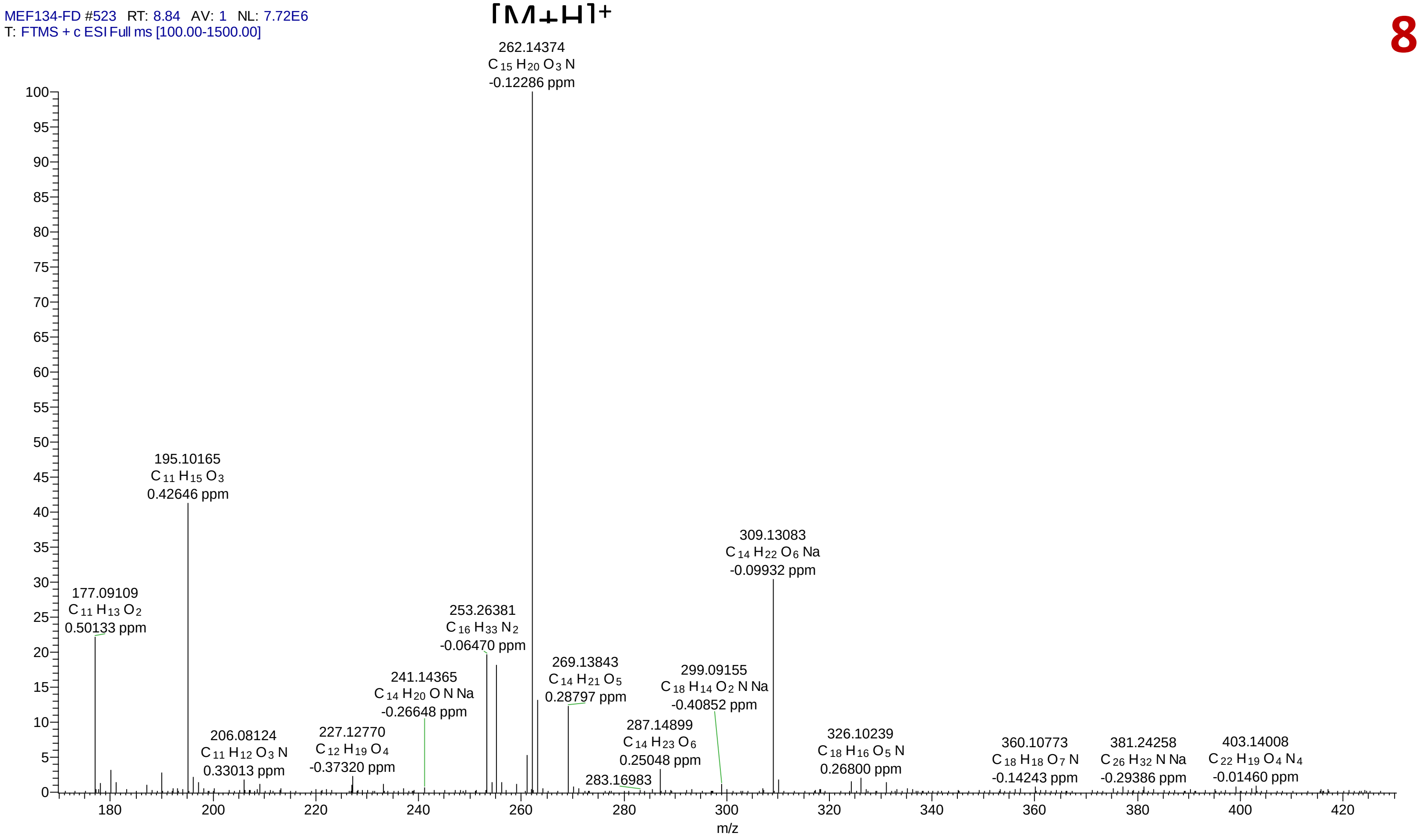

2. MEF134 FD
HPLC-HRMS Spectra

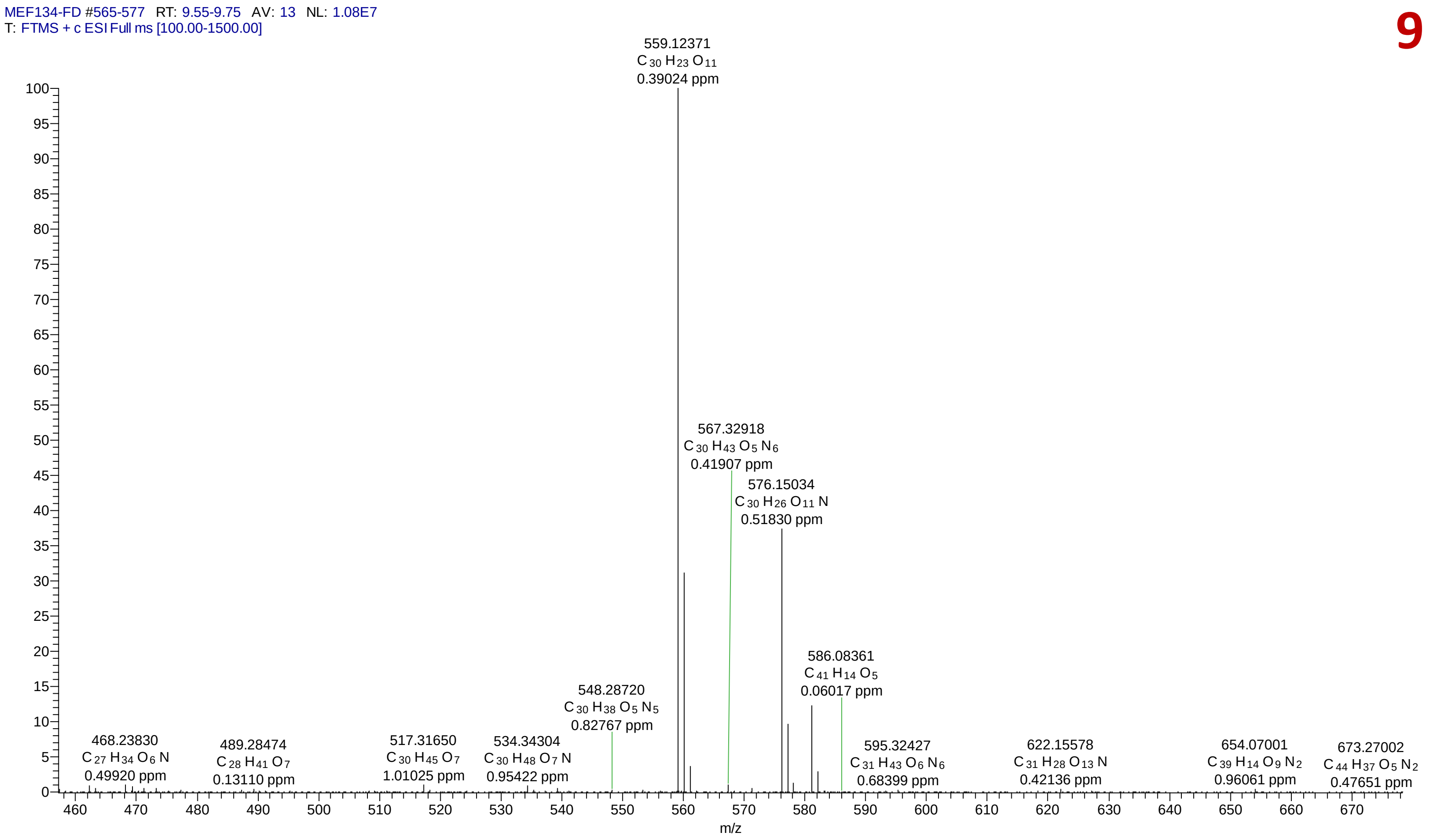

9

2. MEF134 FD
HPLC-HRMS Spectra

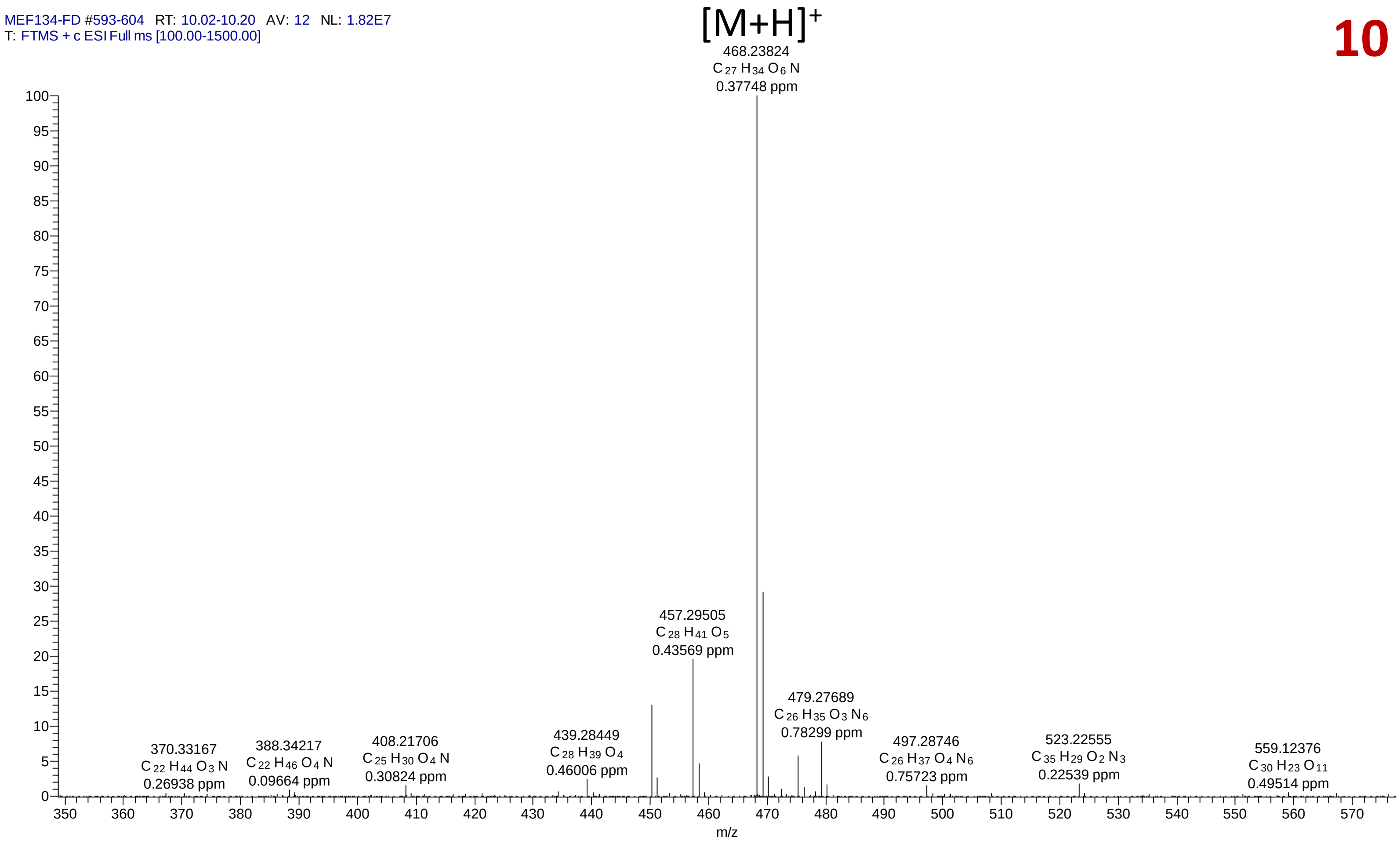

2. MEF134 FD
HPLC-HRMS Spectra

11

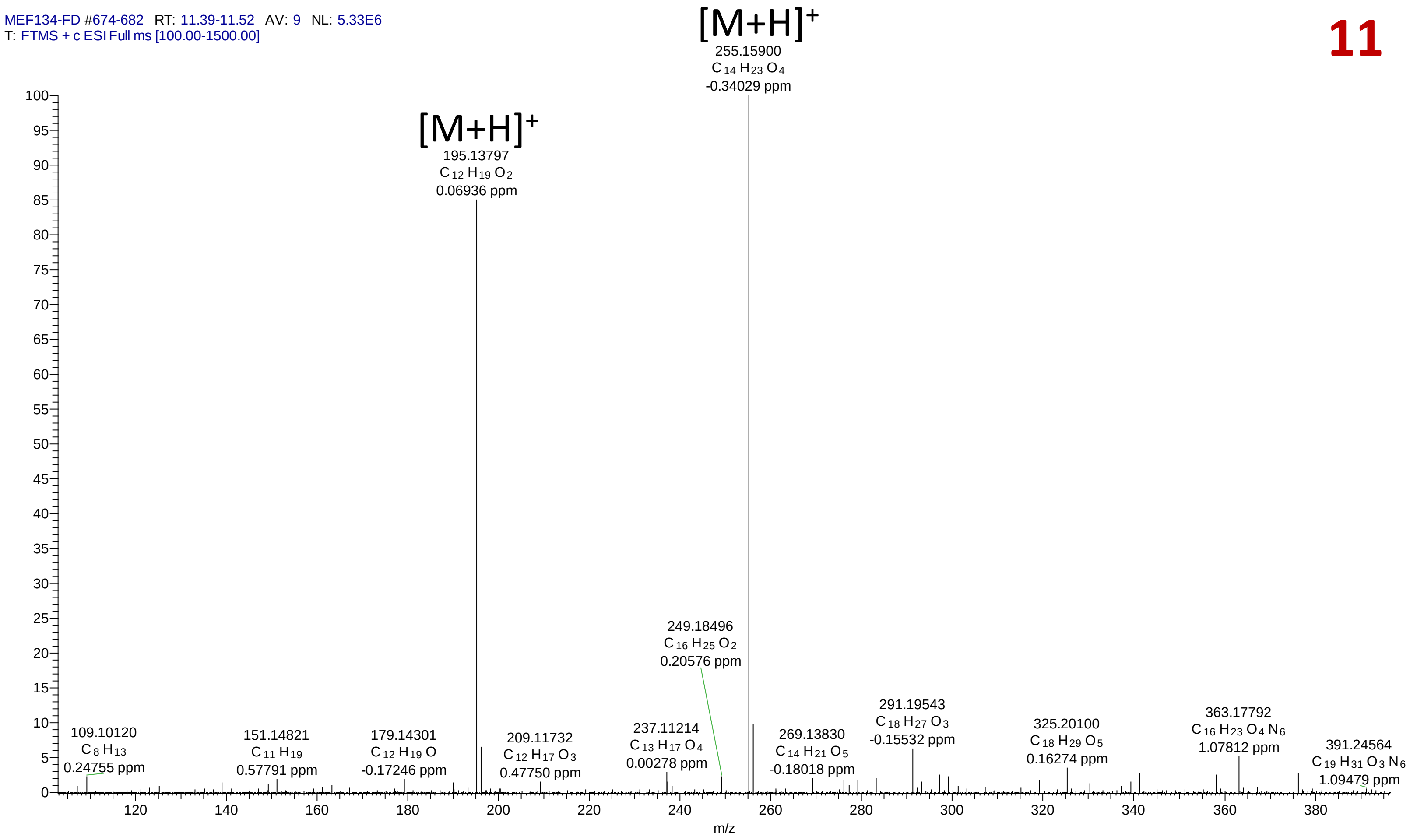

2. MEF134 FD
HPLC-HRMS Spectra

12

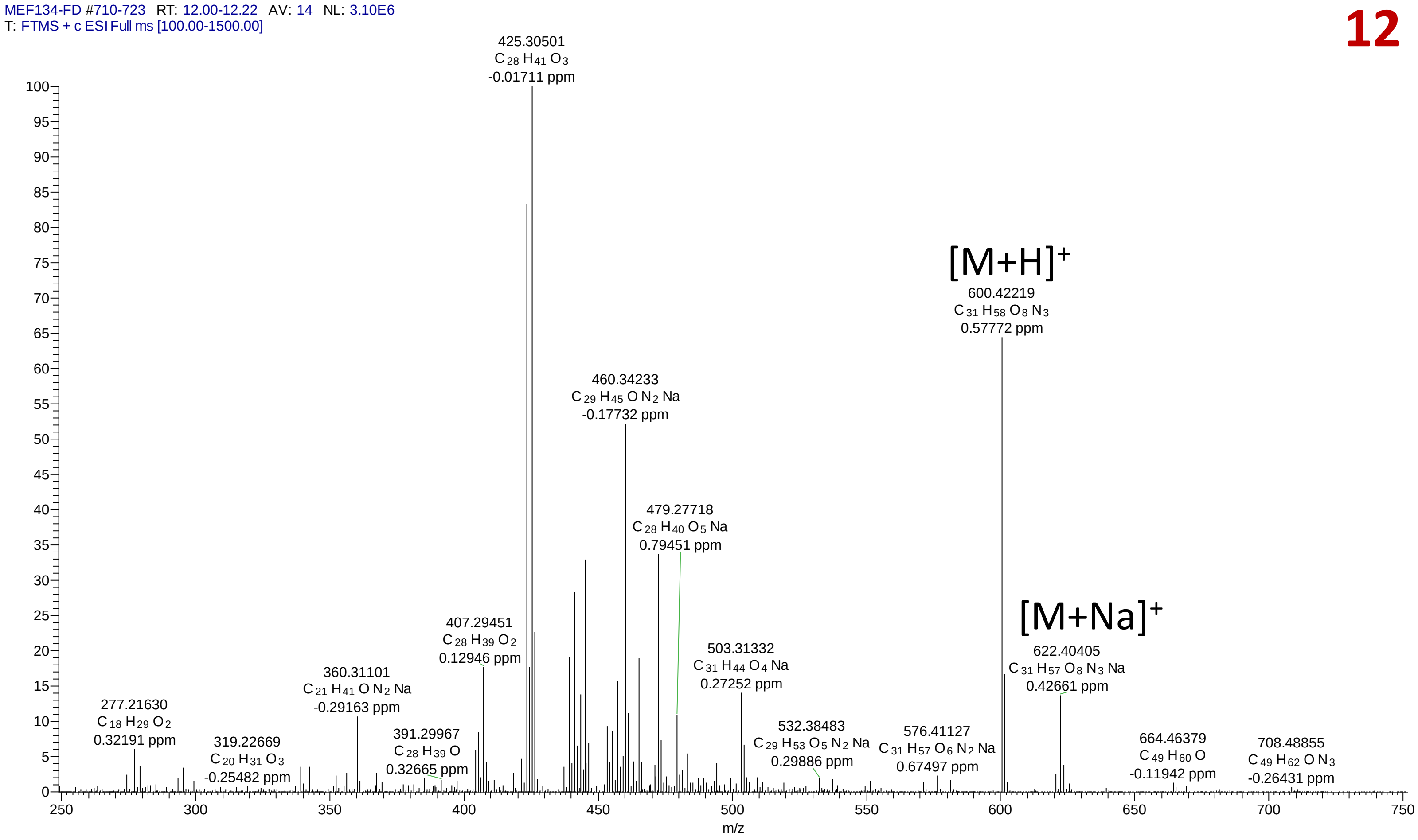

2. MEF134 FD
HPLC-HRMS Spectra

13

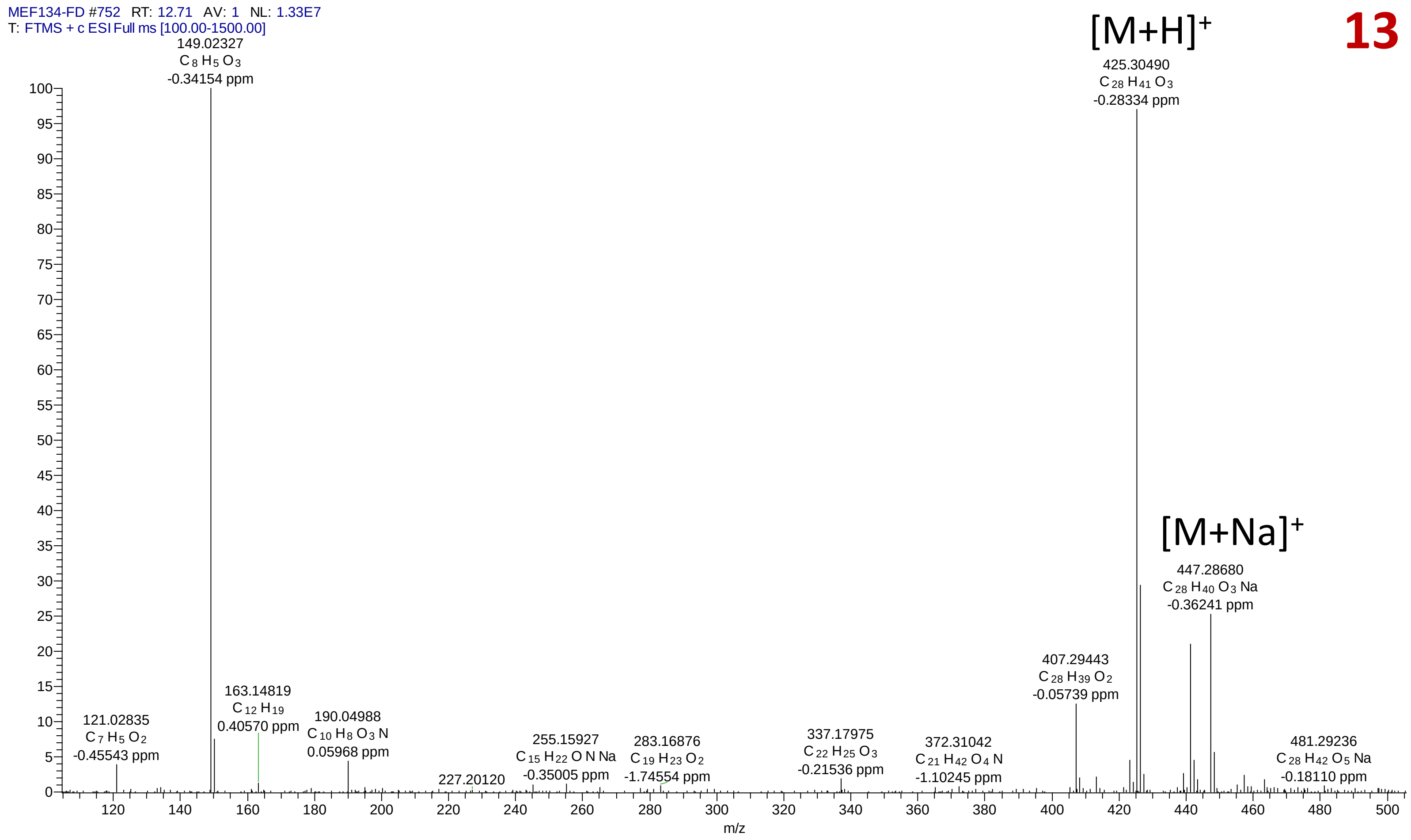

2. MEF134 FD
HPLC-HRMS Spectra

14

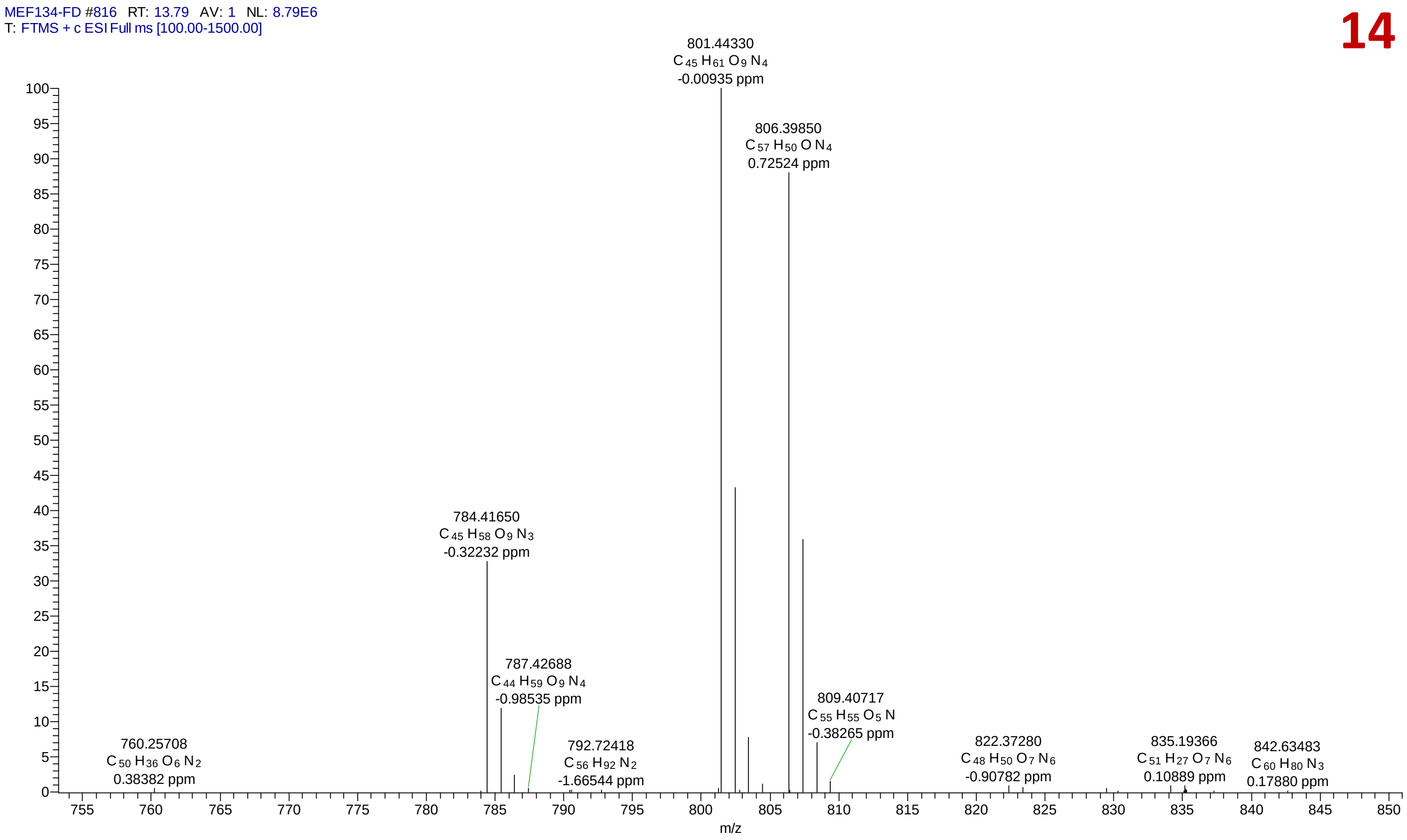

2. MEF134 FD
HPLC-HRMS Spectra

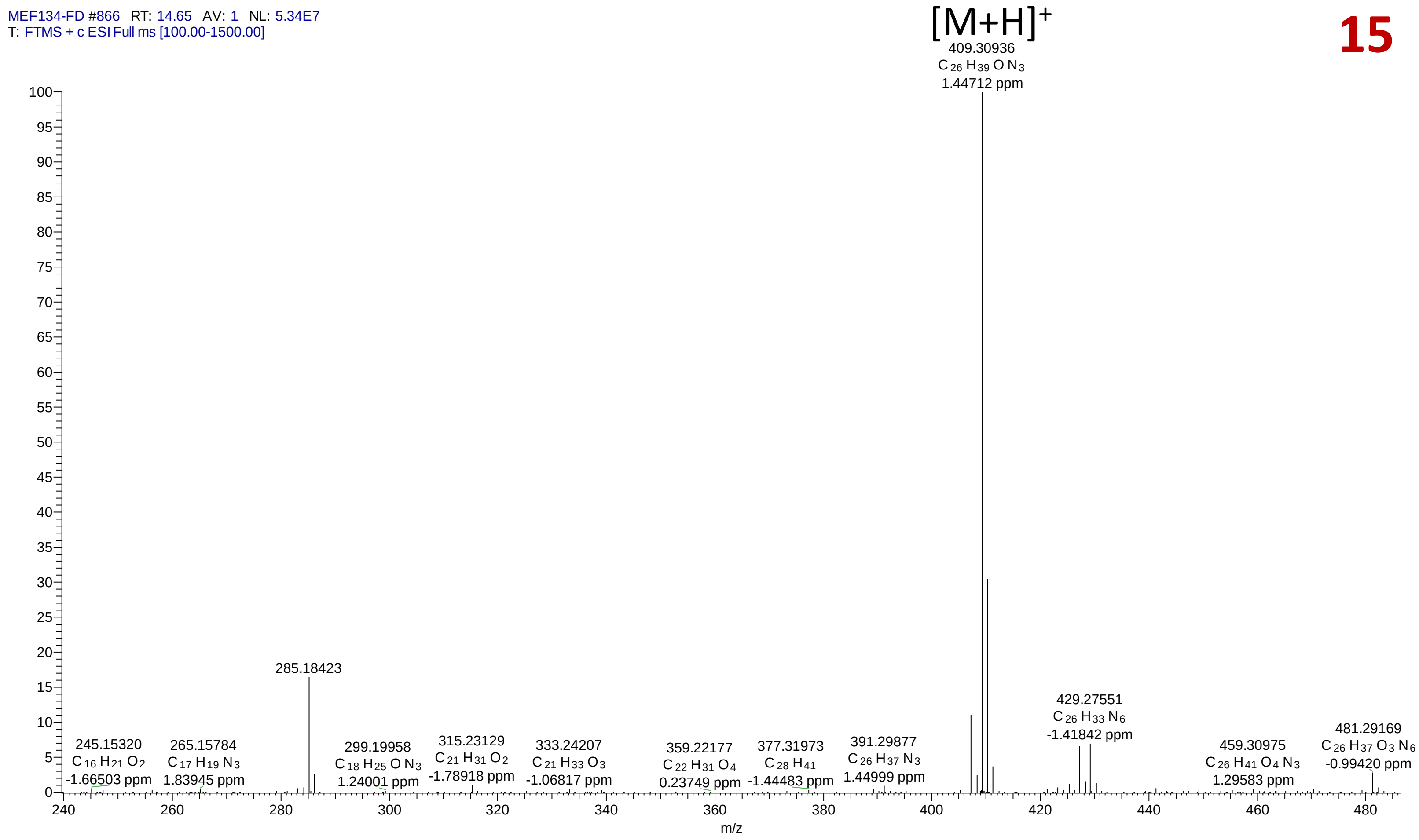

2. MEF134 FD
HPLC-HRMS Spectra

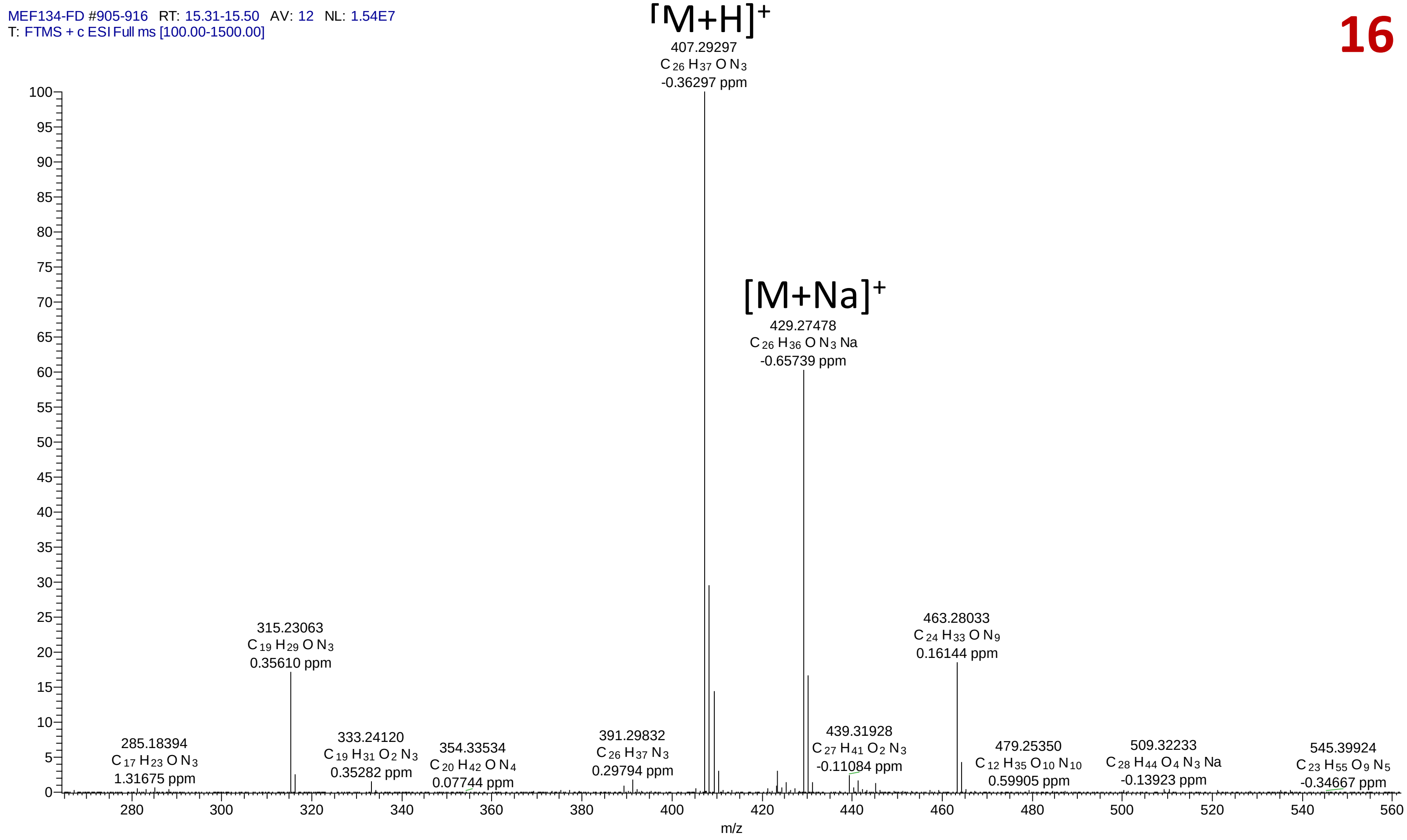

16

2. MEF134 FD
HPLC-HRMS Spectra

17

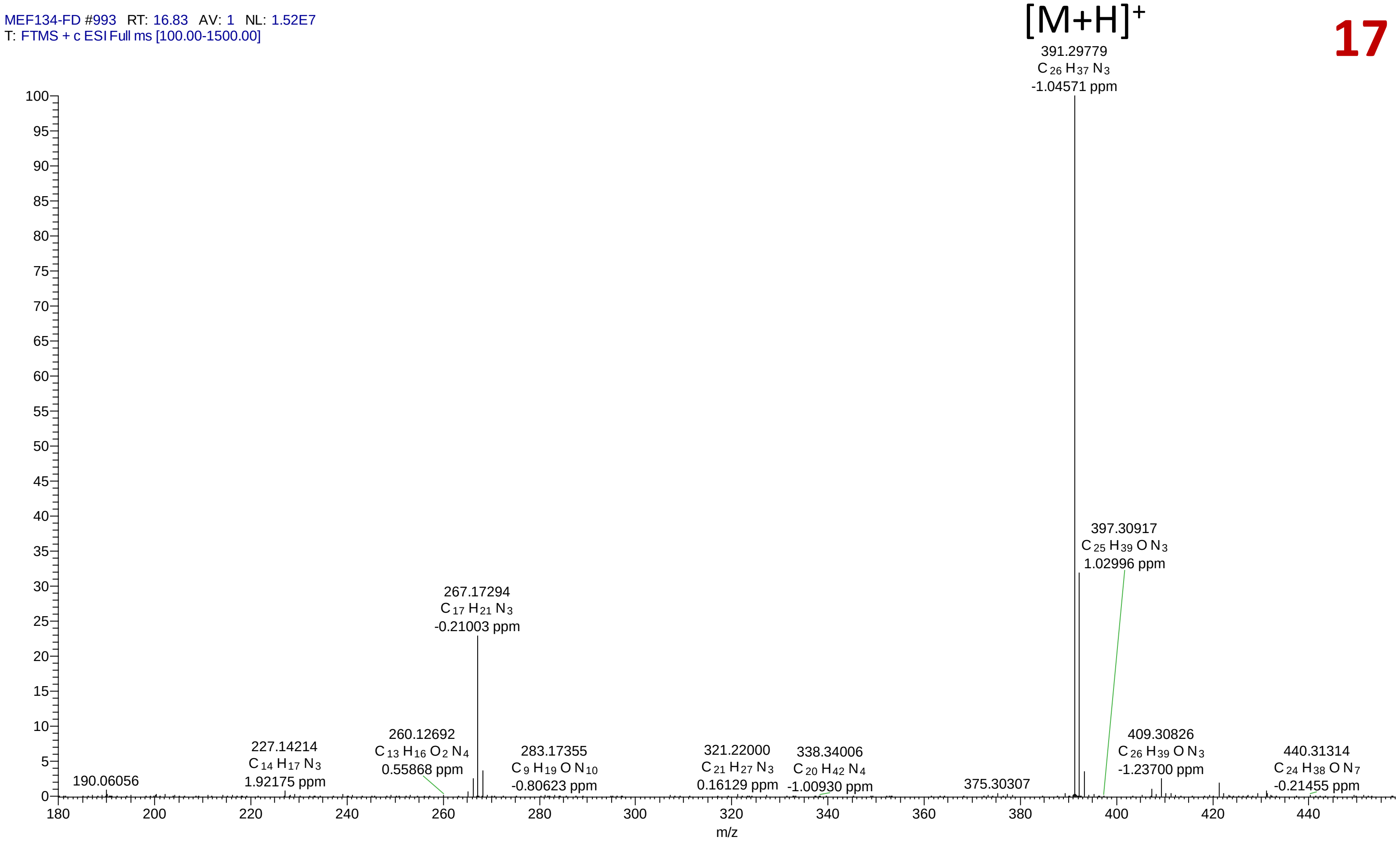

3. MEF134 FD V5
HPLC-HRMS Fullscan

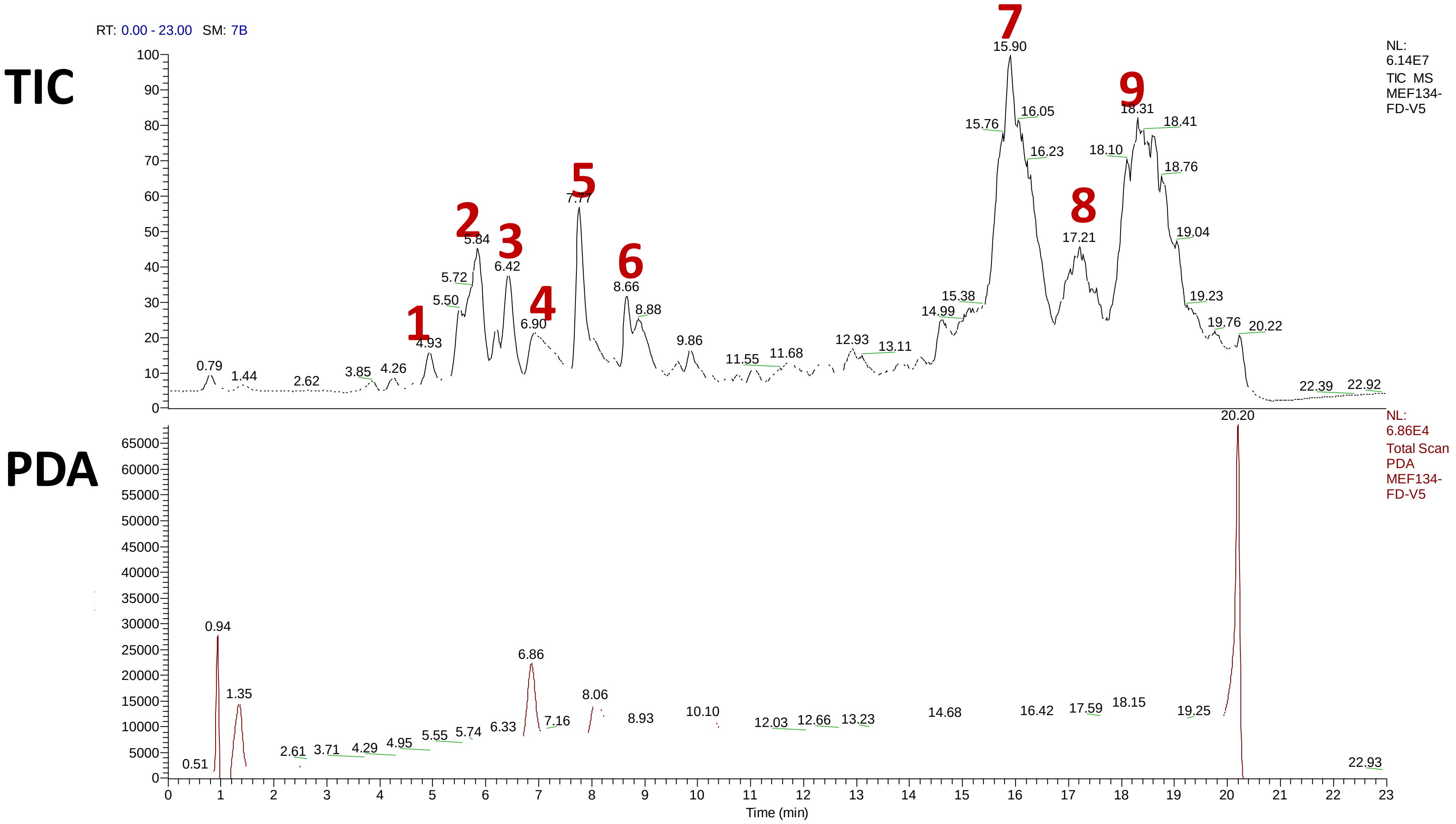

3. MEF134 FD V5
HPLC-HRMS Spectra

1

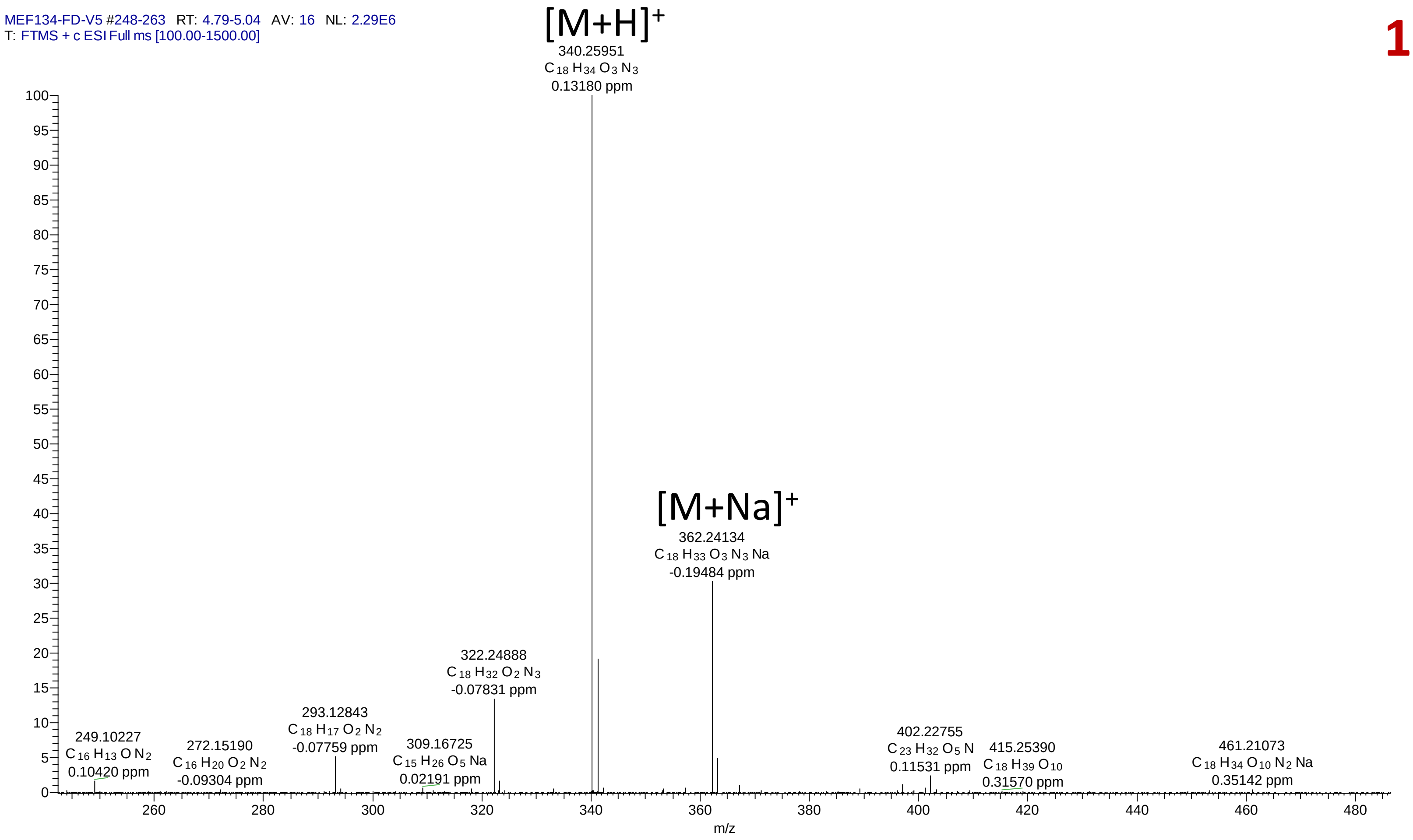

3. MEF134 FD V5
HPLC-HRMS Spectra

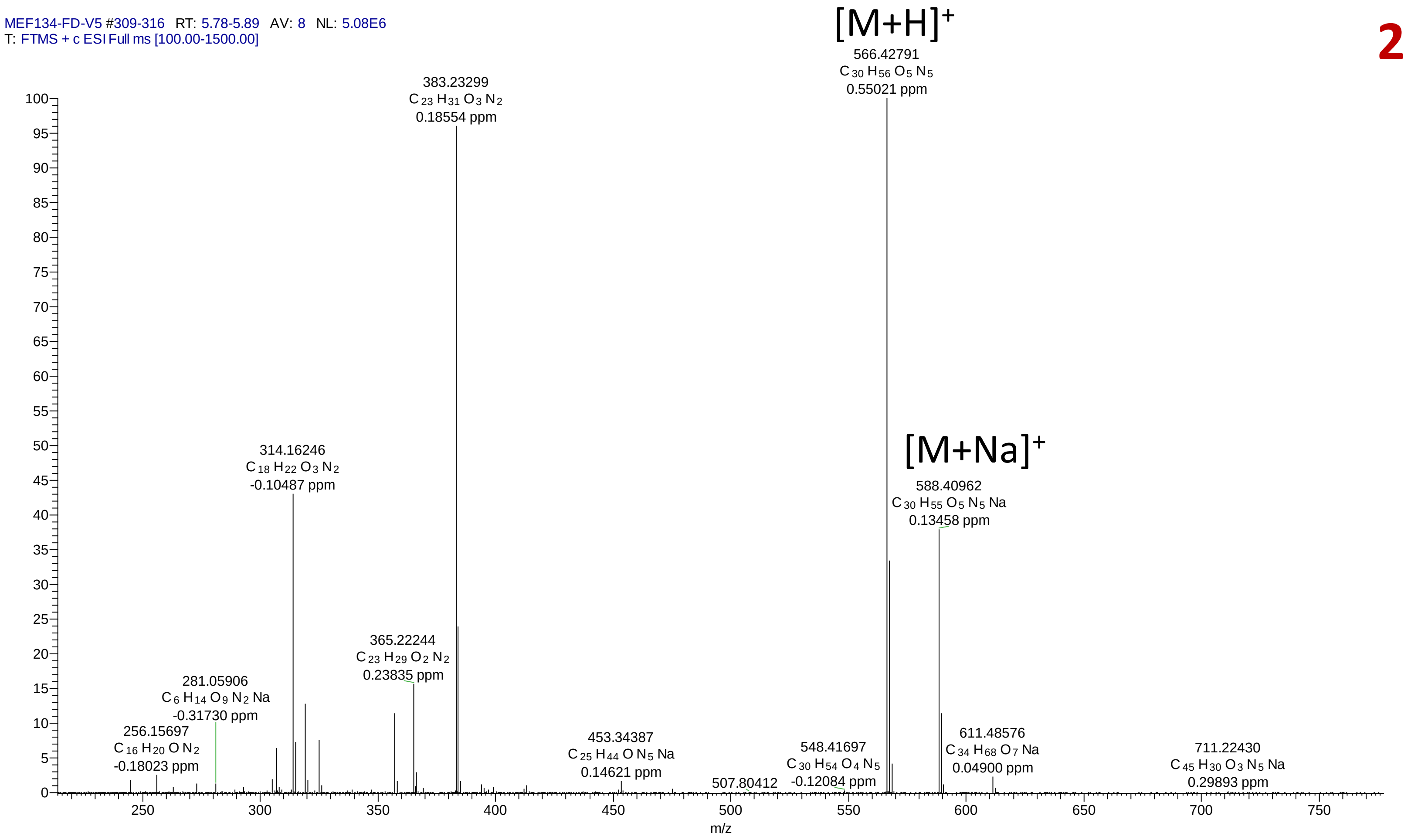

MEF FD V5
HPLC-HRMS Spectra

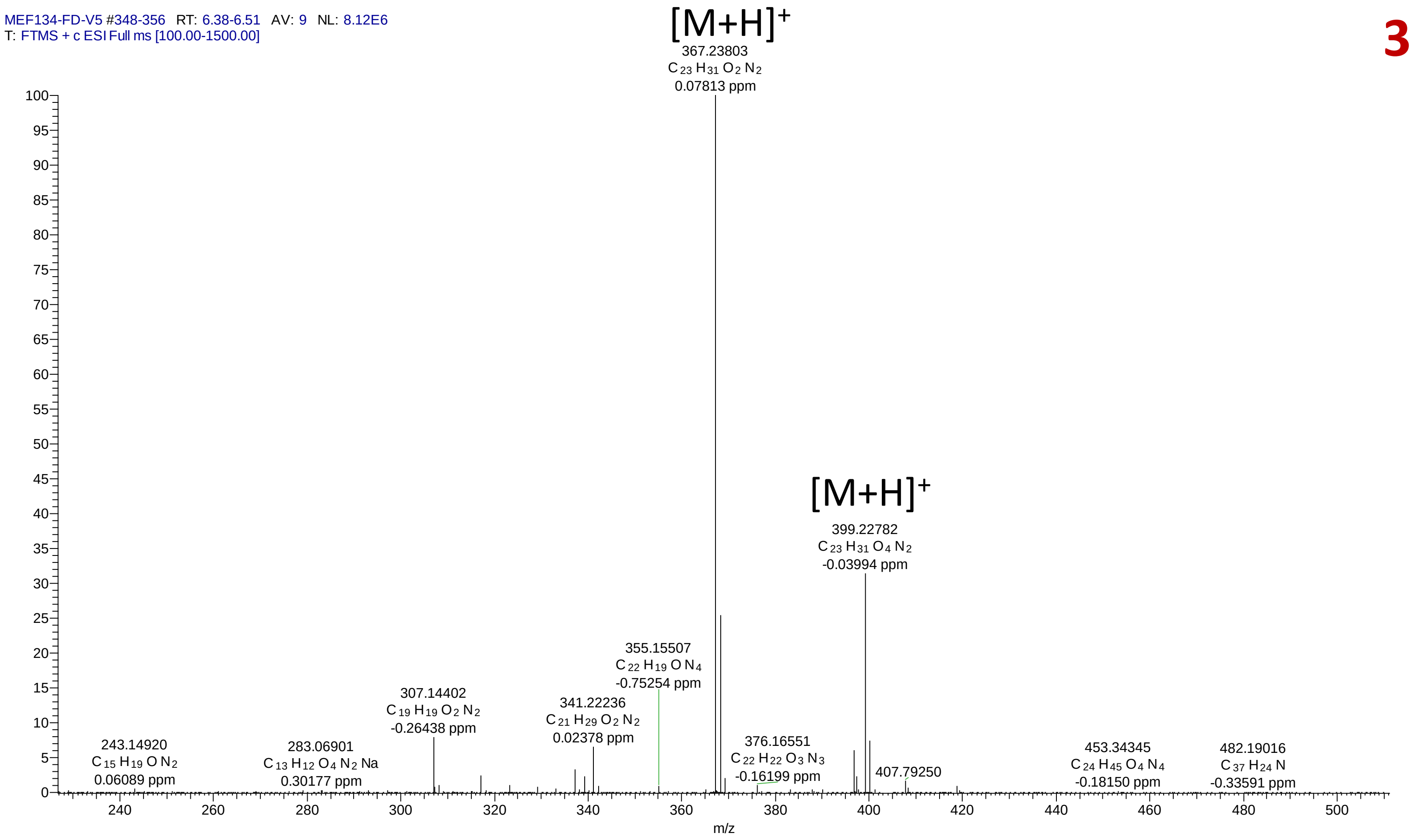

3. MEF134 FD V5
HPLC-HRMS Spectra

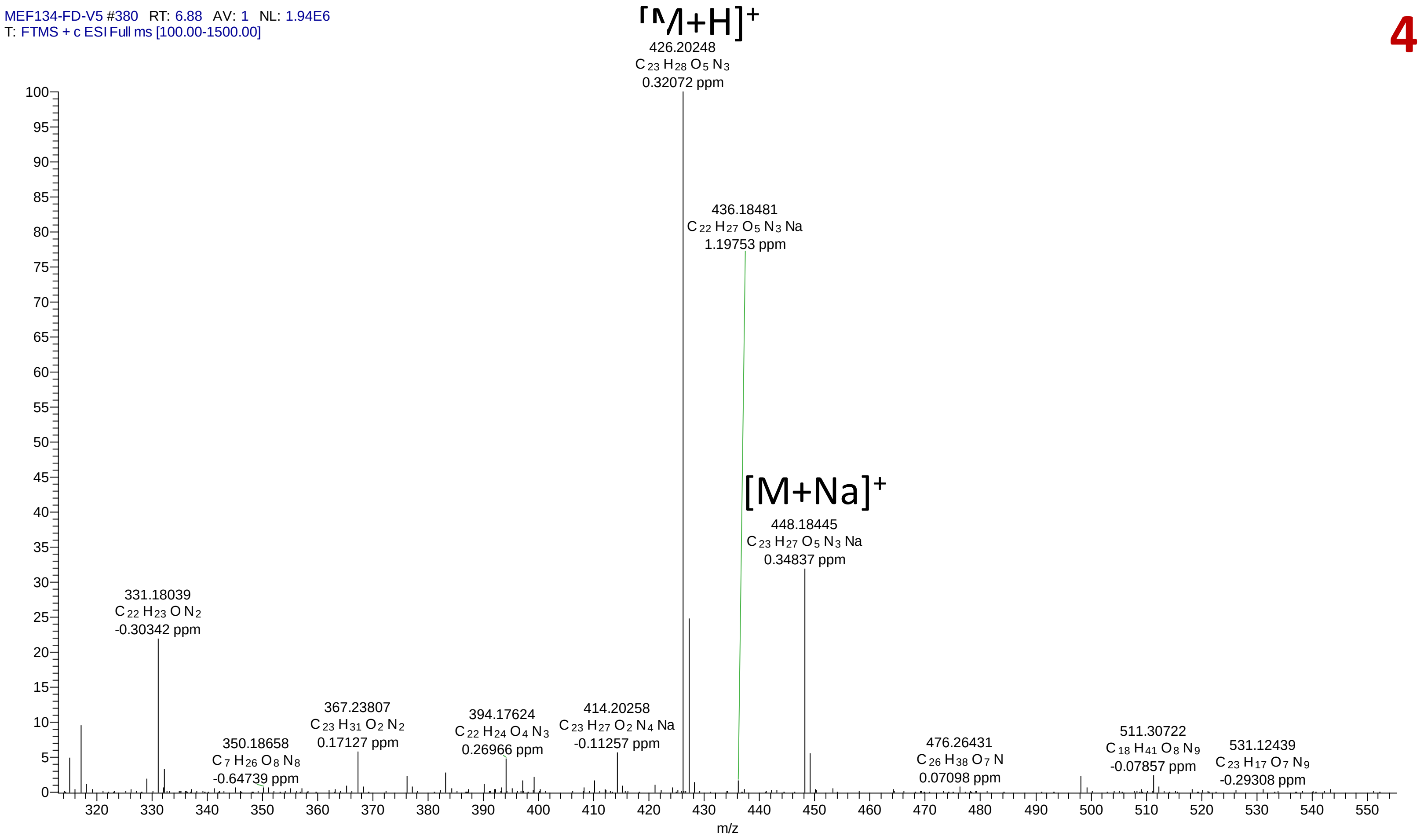

3. MEF134 FD V5
HPLC-HRMS Spectra

5

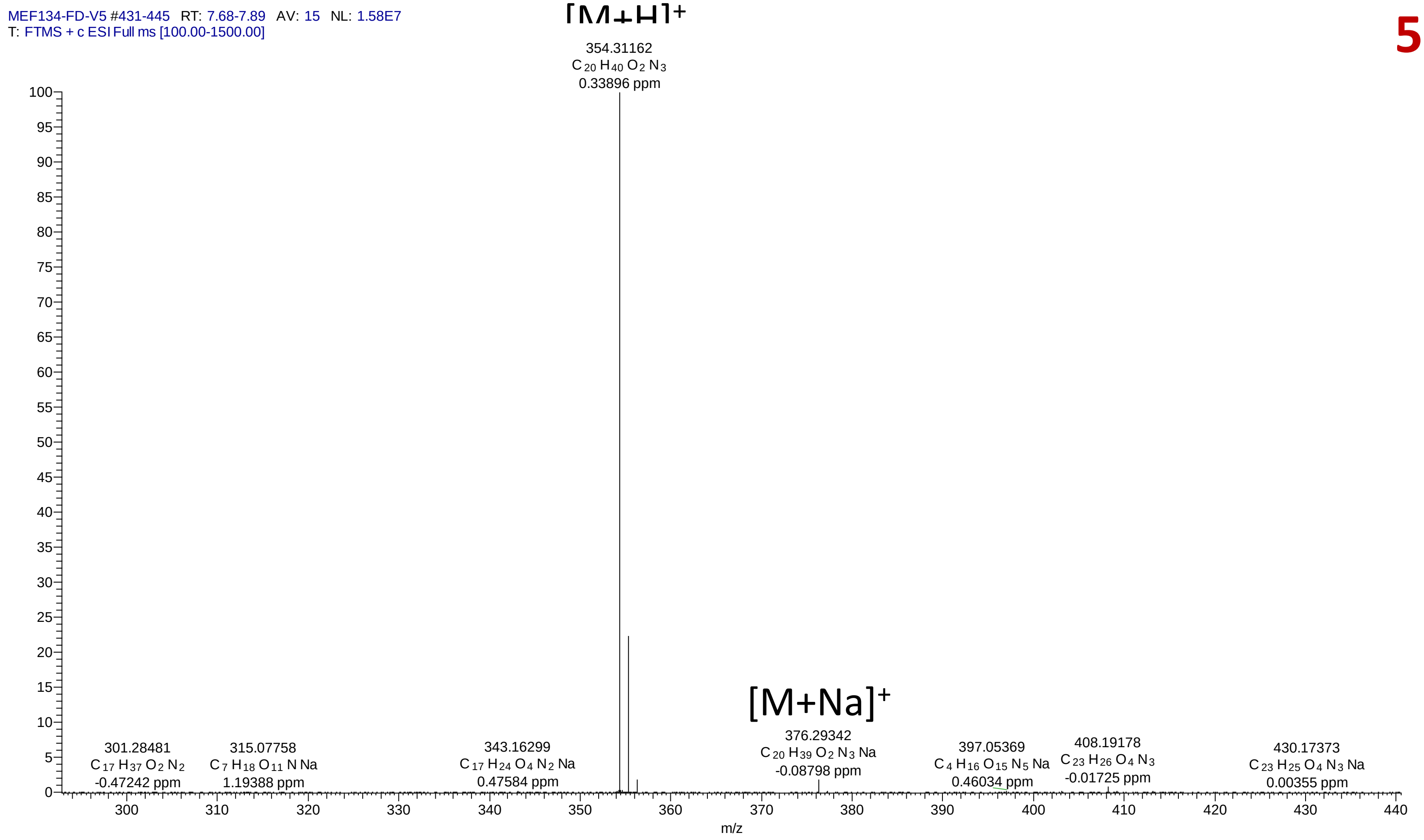

3. MEF134 FD V5
HPLC-HRMS Spectra

6

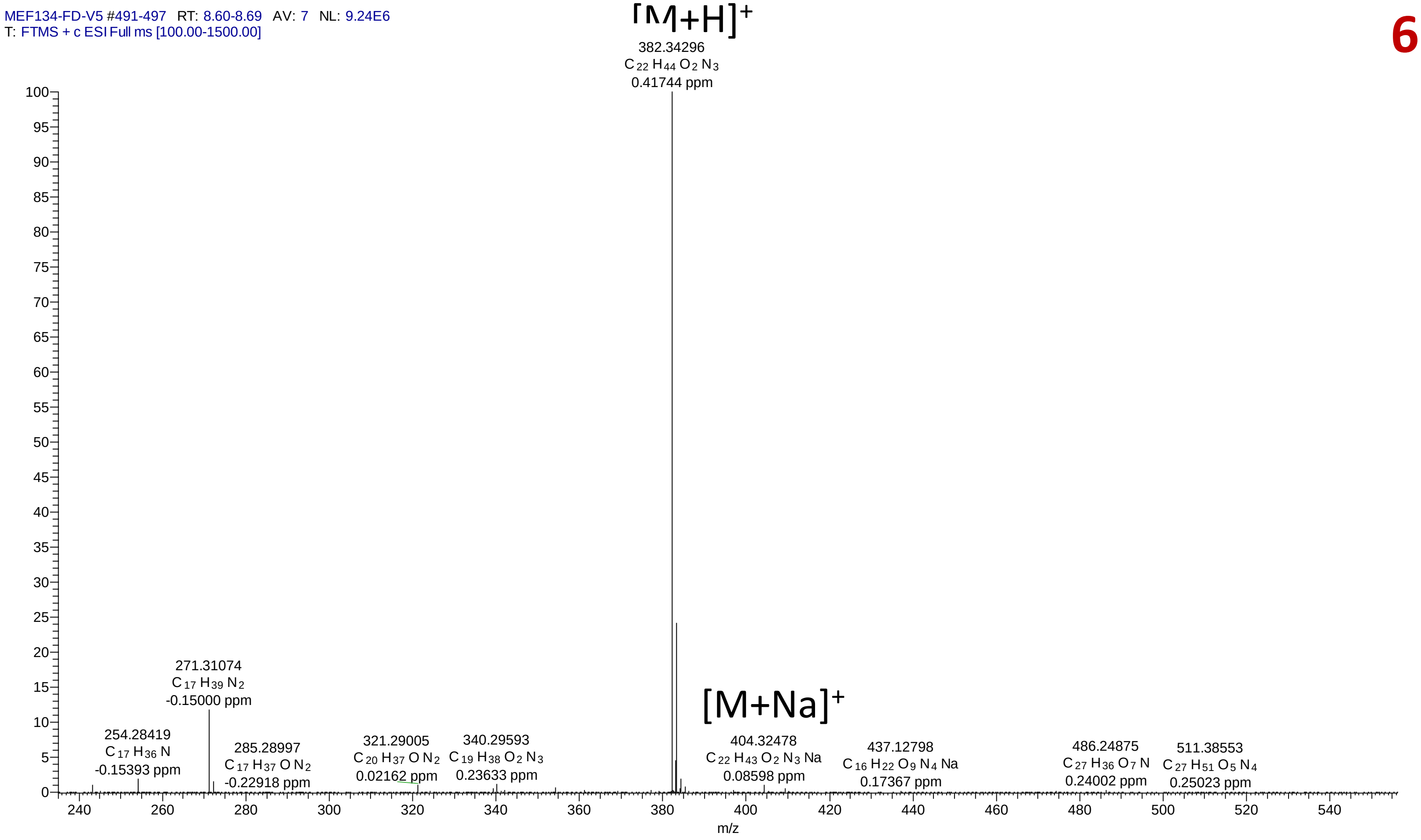

3. MEF134 FD V5
HPLC-HRMS Spectra

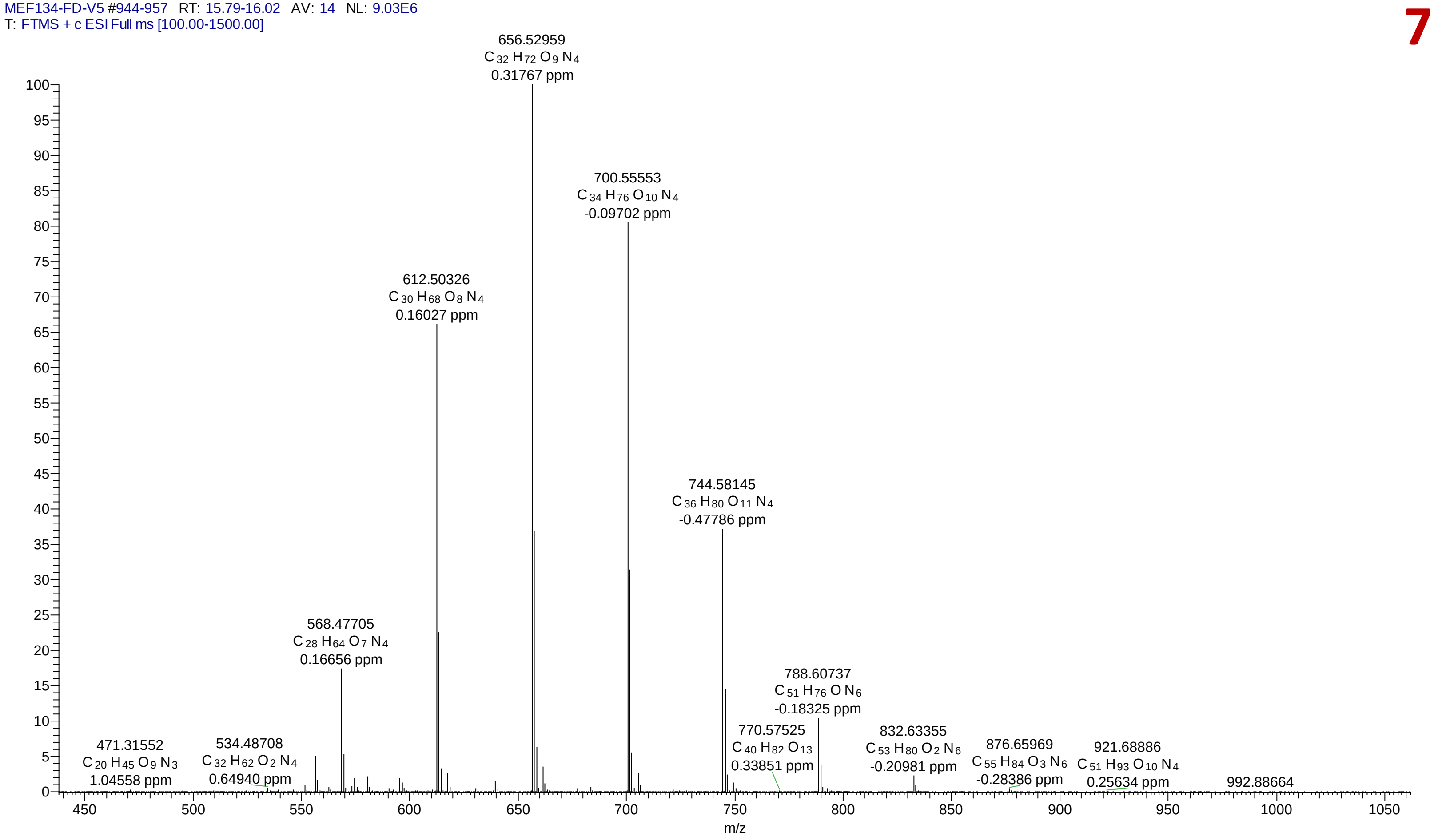

7

3. MEF134 FD V5
HPLC-HRMS Spectra

8

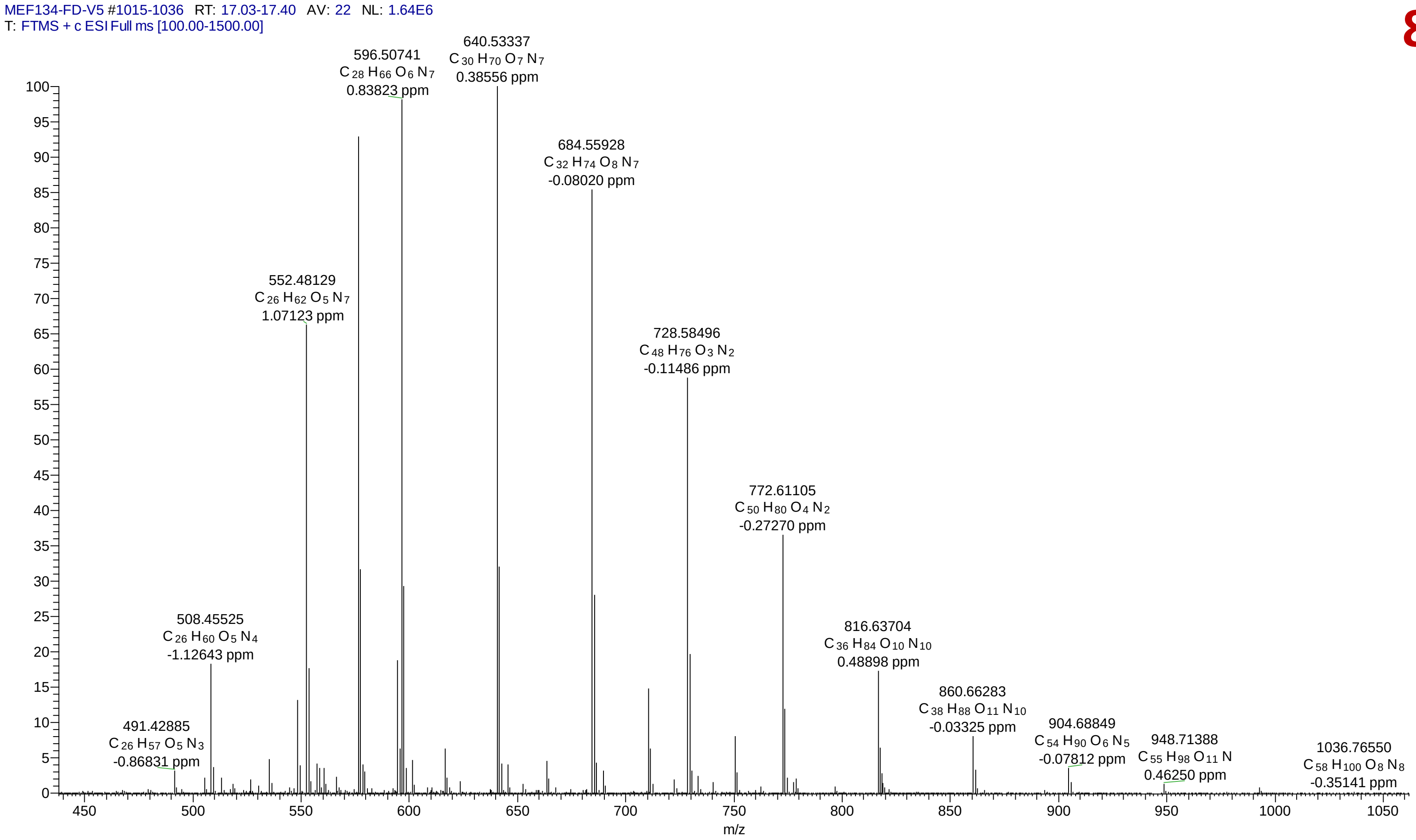

3. MEF134 FD V5
HPLC-HRMS Spectra

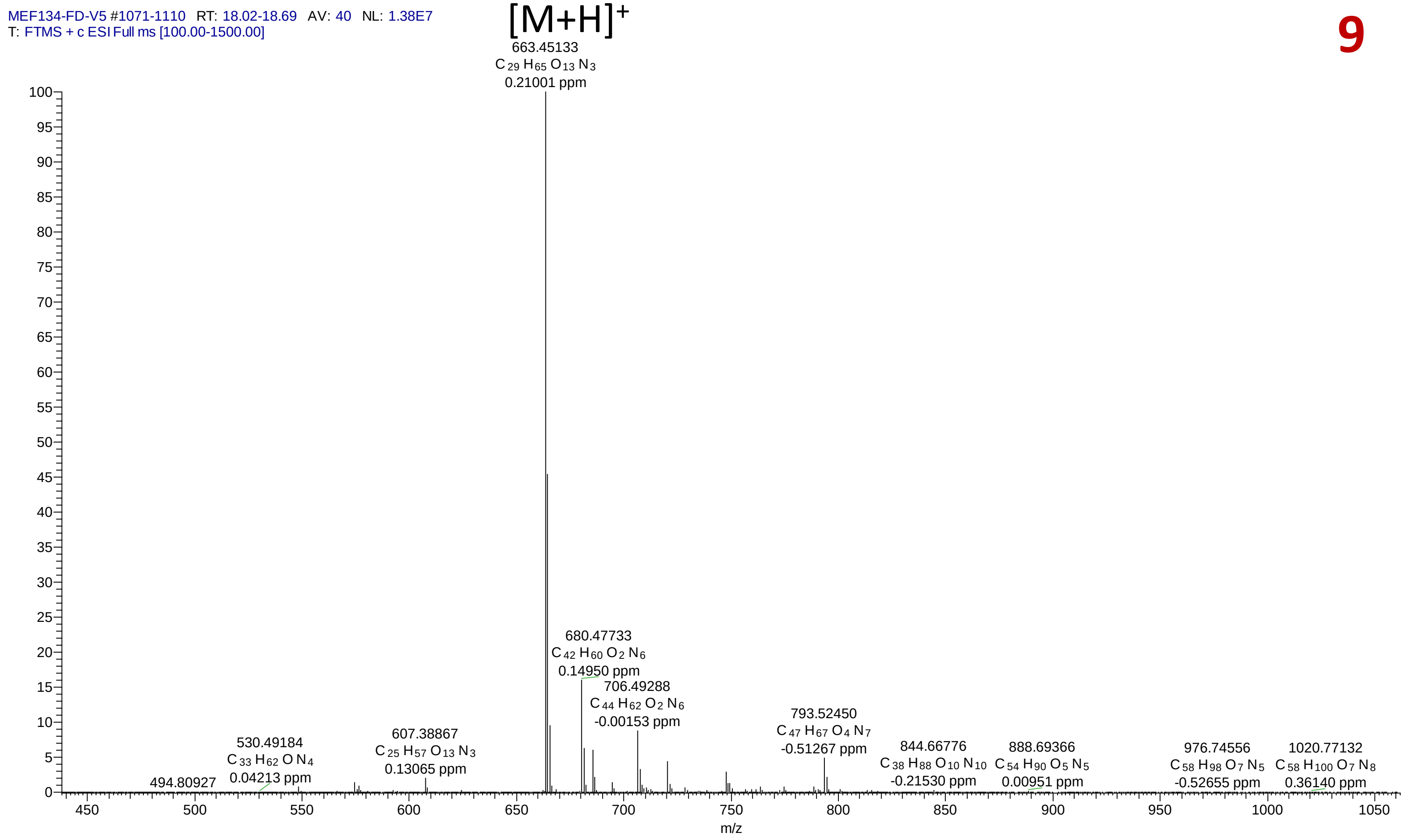

4. MEF134 FD V5 V7 HPLC-HRMS Fullscan

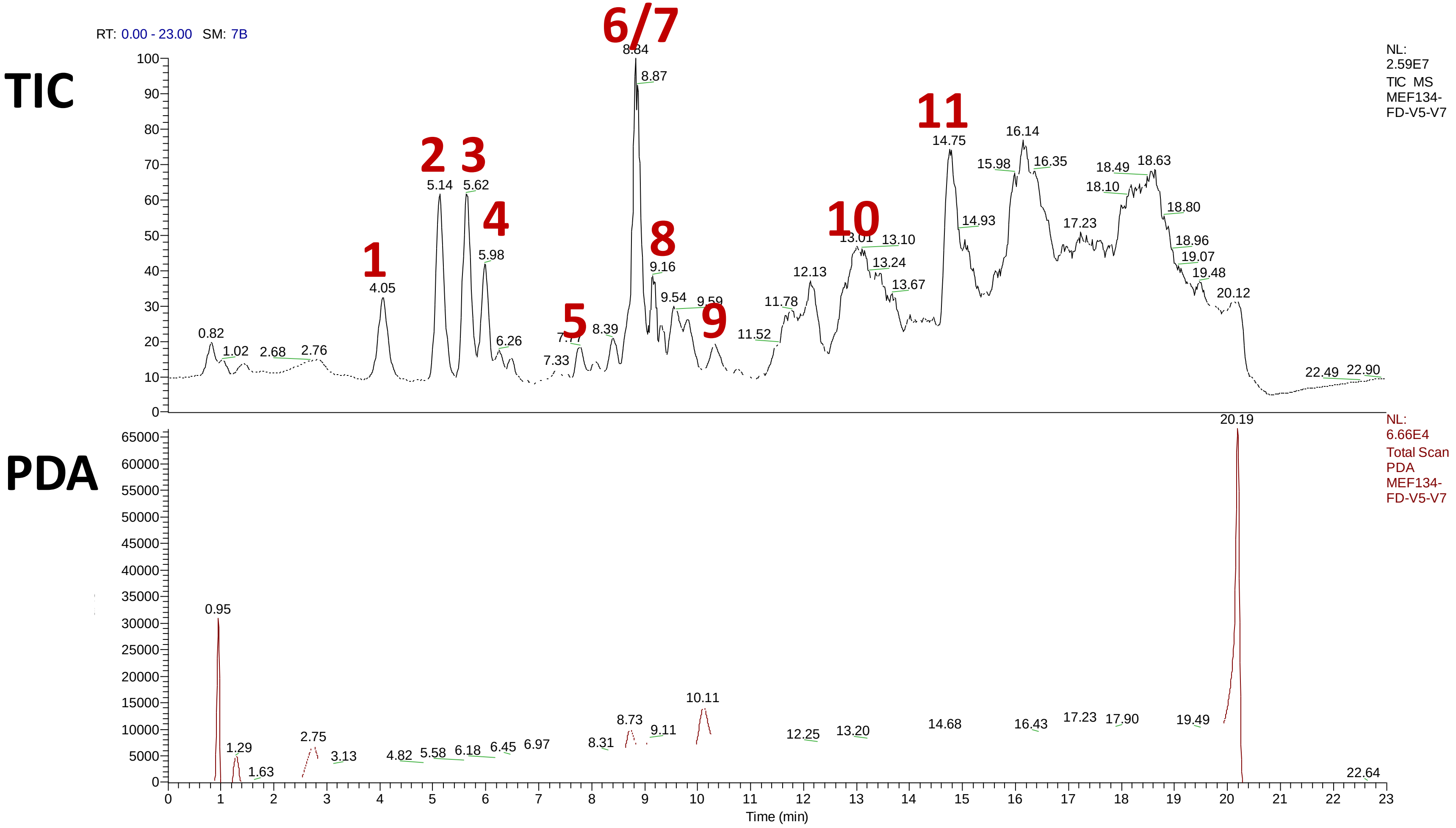

4. MEF134 FD V5 V7 HPLC-HRMS Spectra

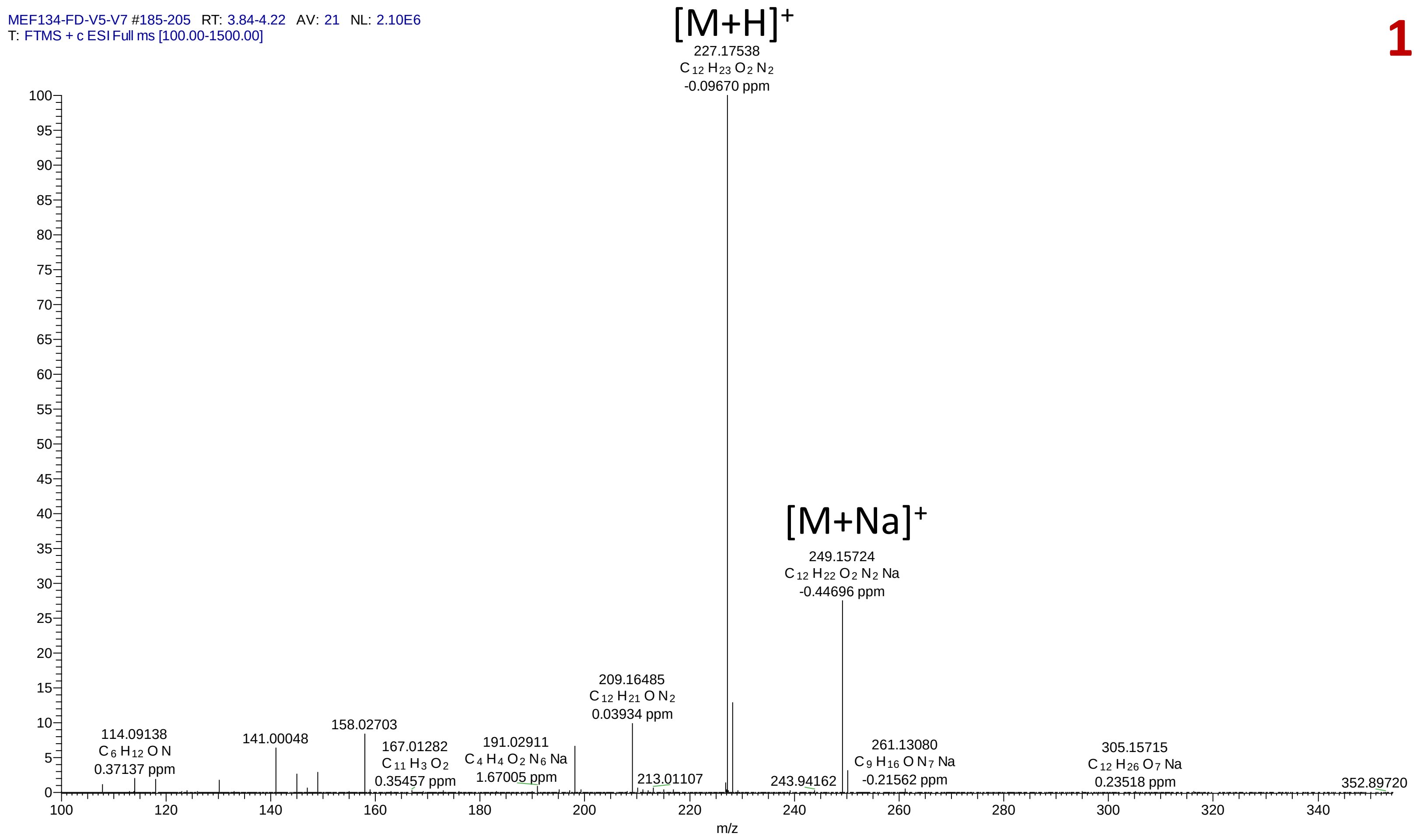

4. MEF134 FD V5 V7 HPLC-HRMS Spectra

2

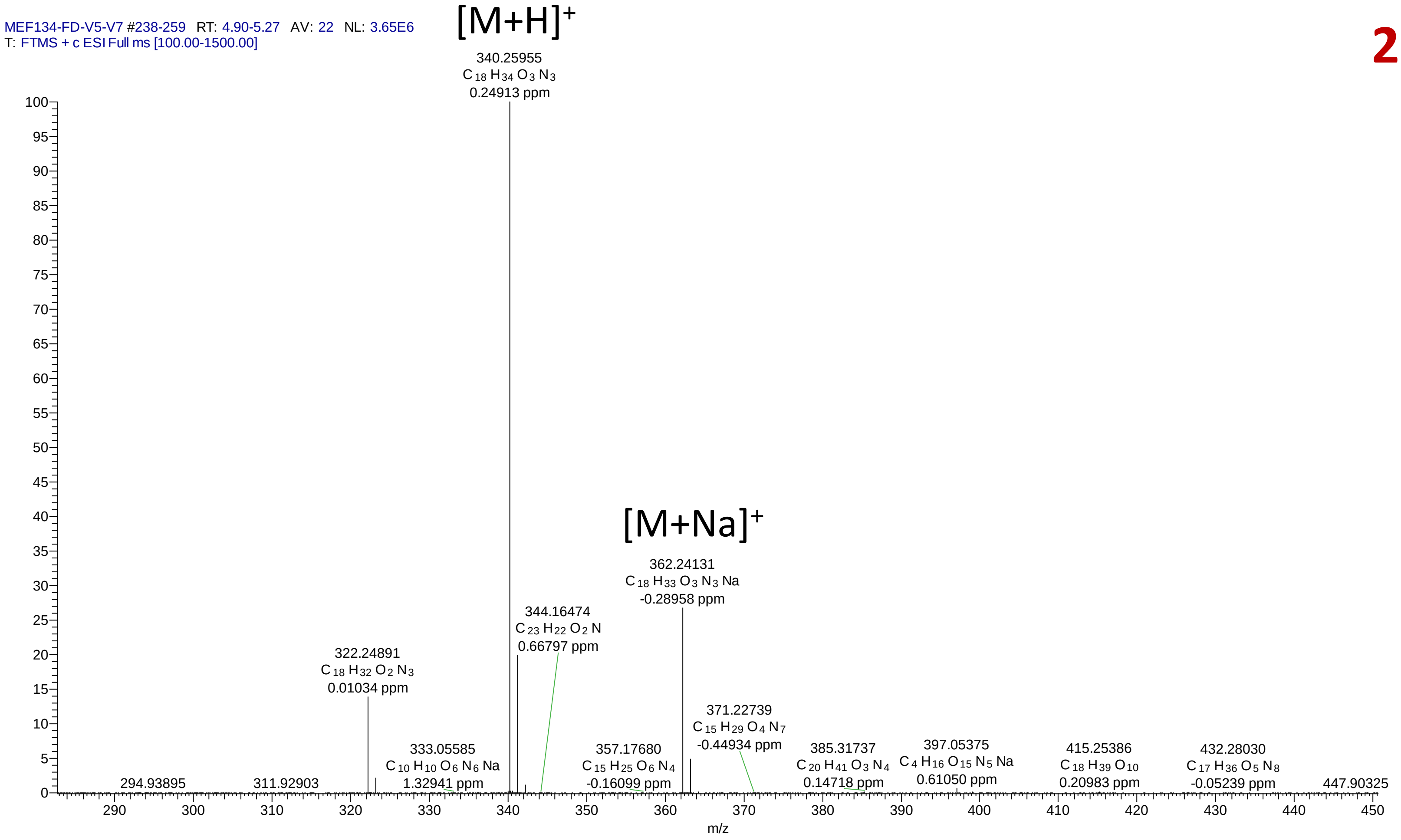

4. MEF134 FD V5 V7 HPLC-HRMS Spectra

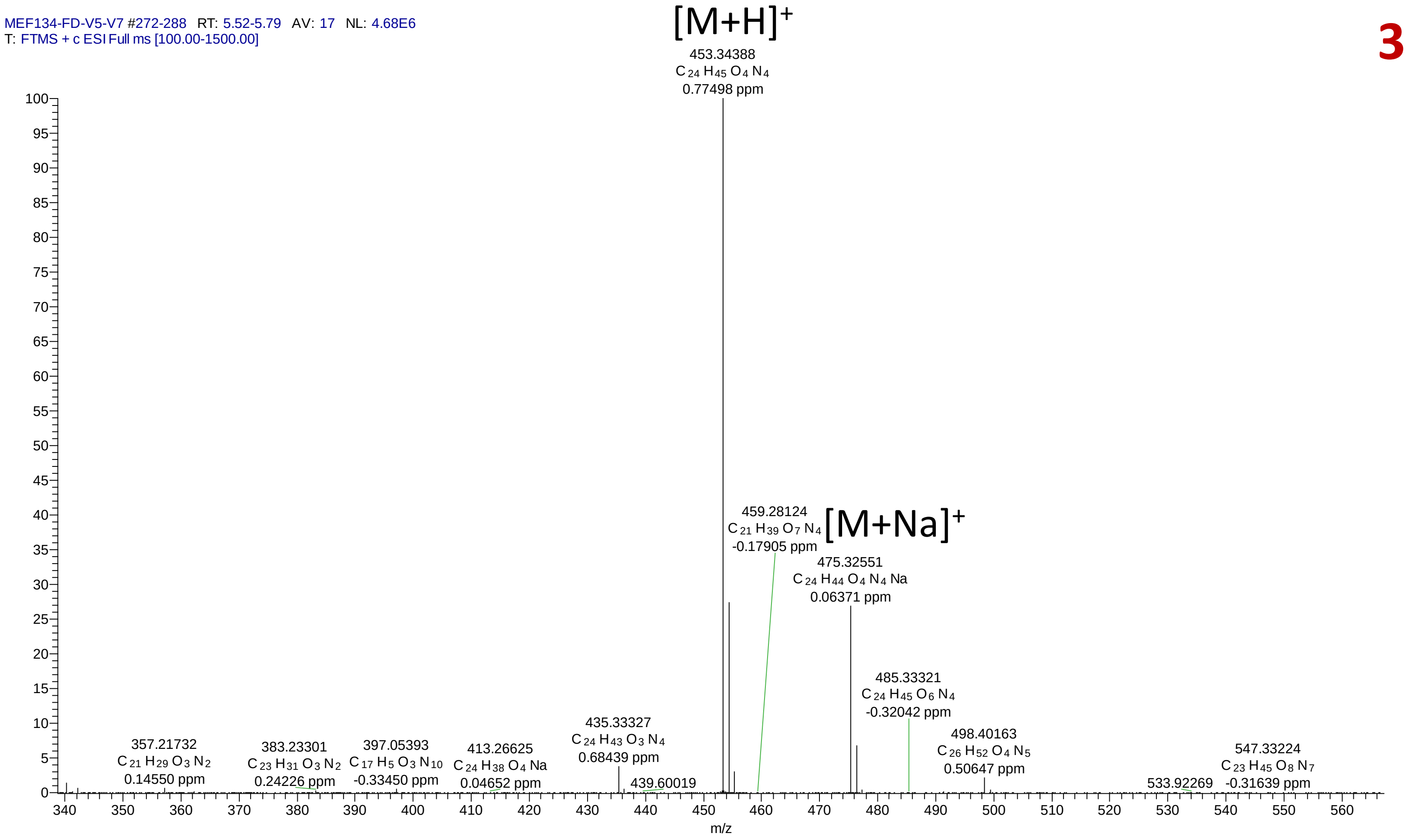

3

4. MEF134 FD V5 V7 HPLC-HRMS Spectra

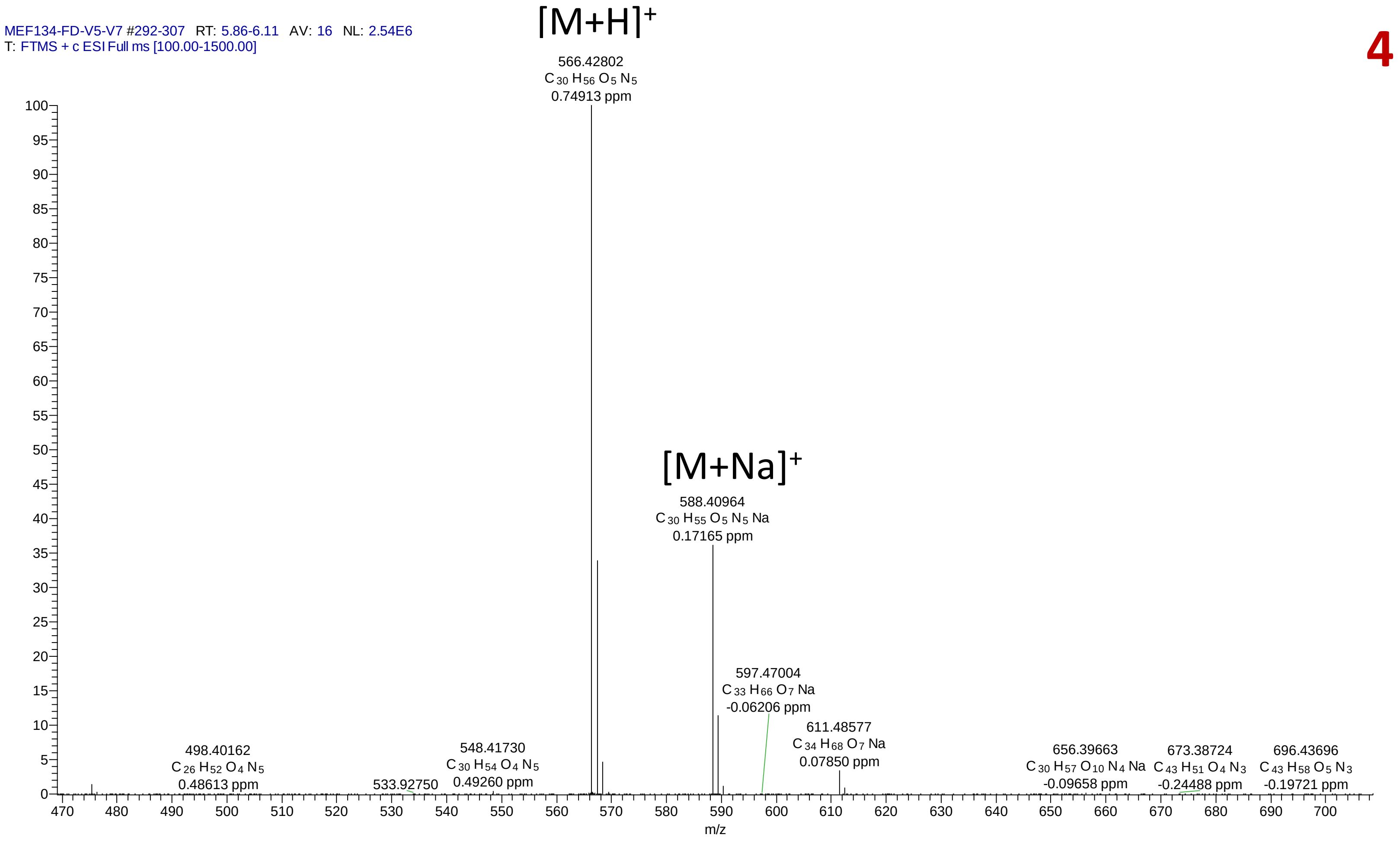

4. MEF134 FD V5 V7
HPLC-HRMS Spectra

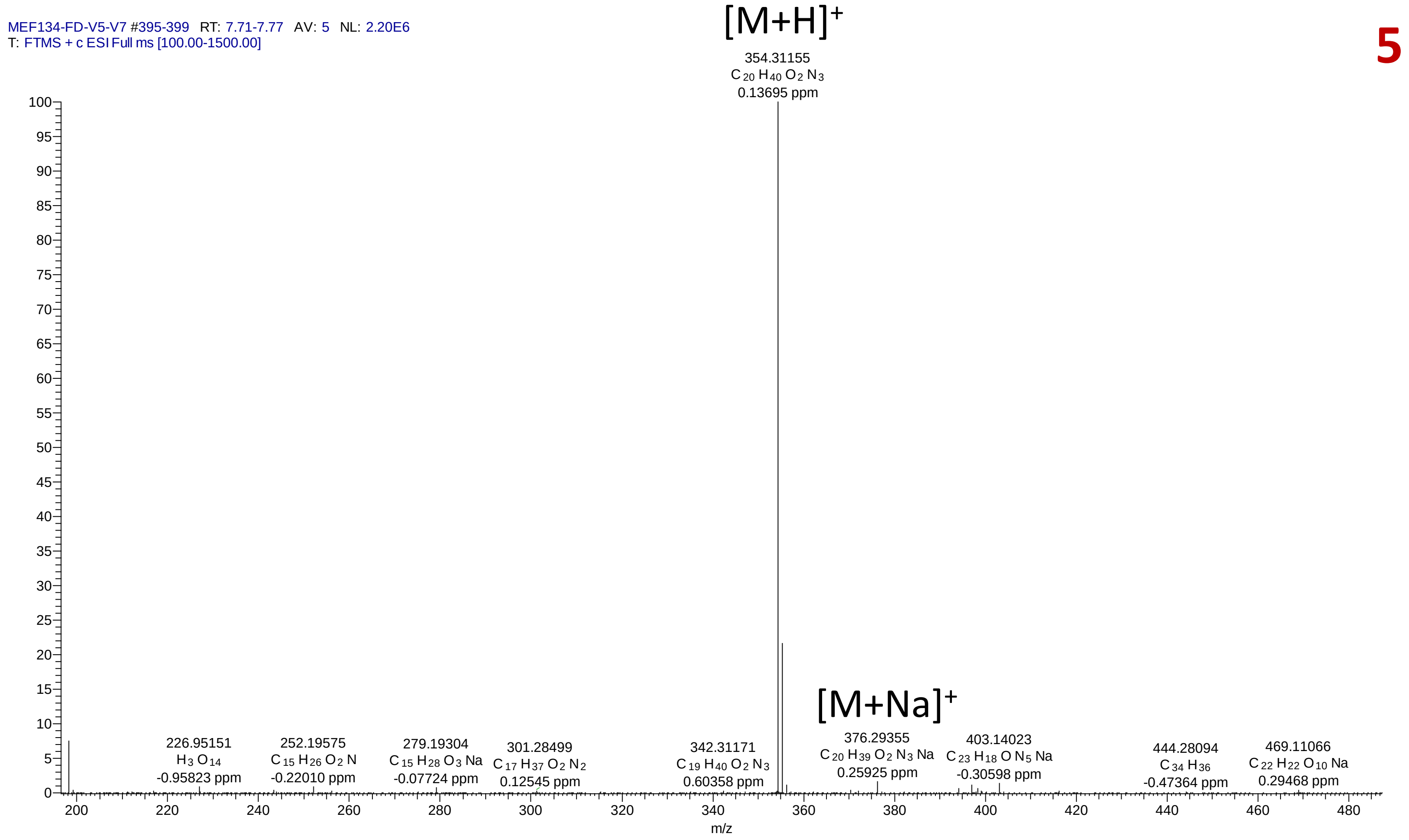

4. MEF134 FD V5 V7 HPLC-HRMS Spectra

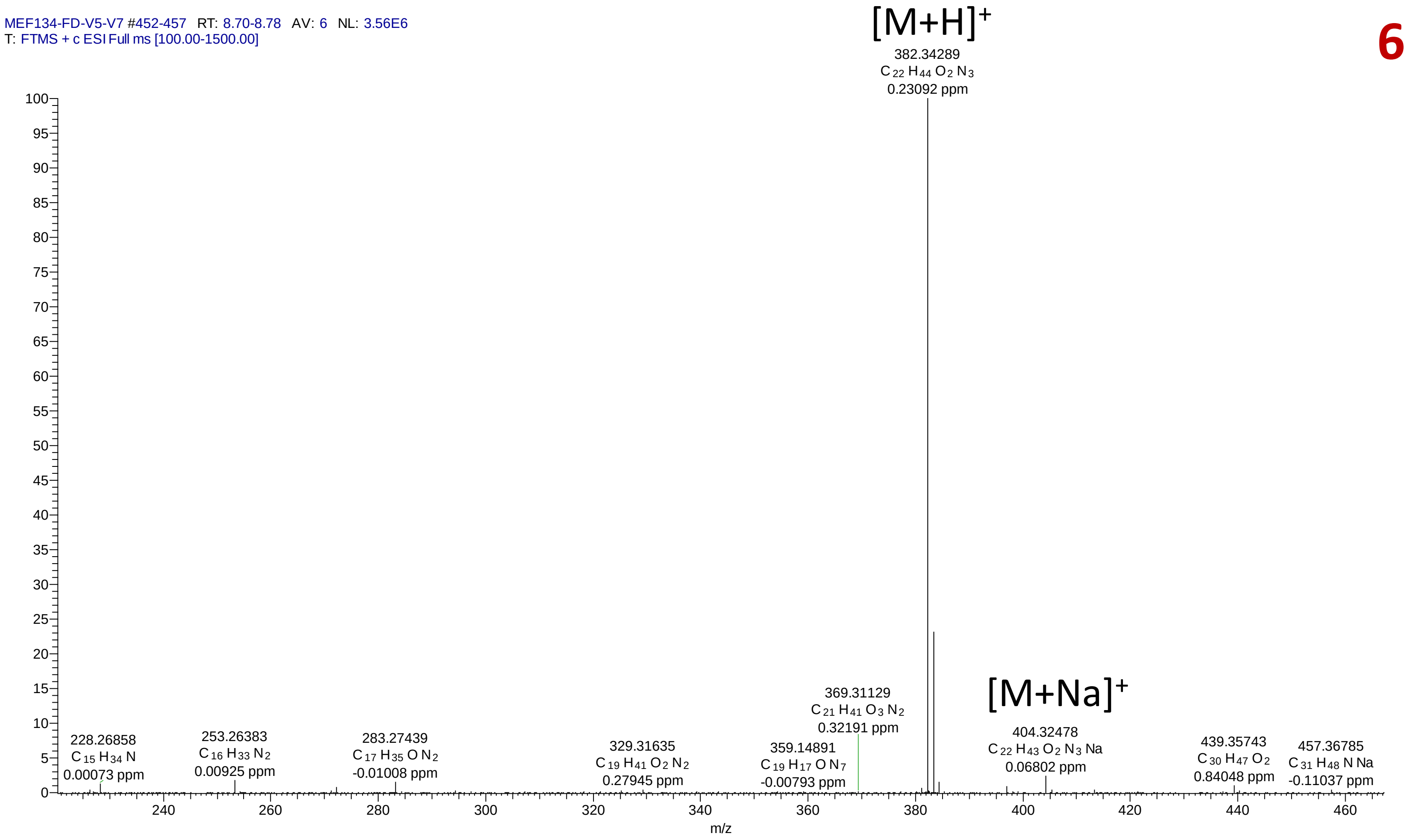

4. MEF134 FD V5 V7 HPLC-HRMS Spectra

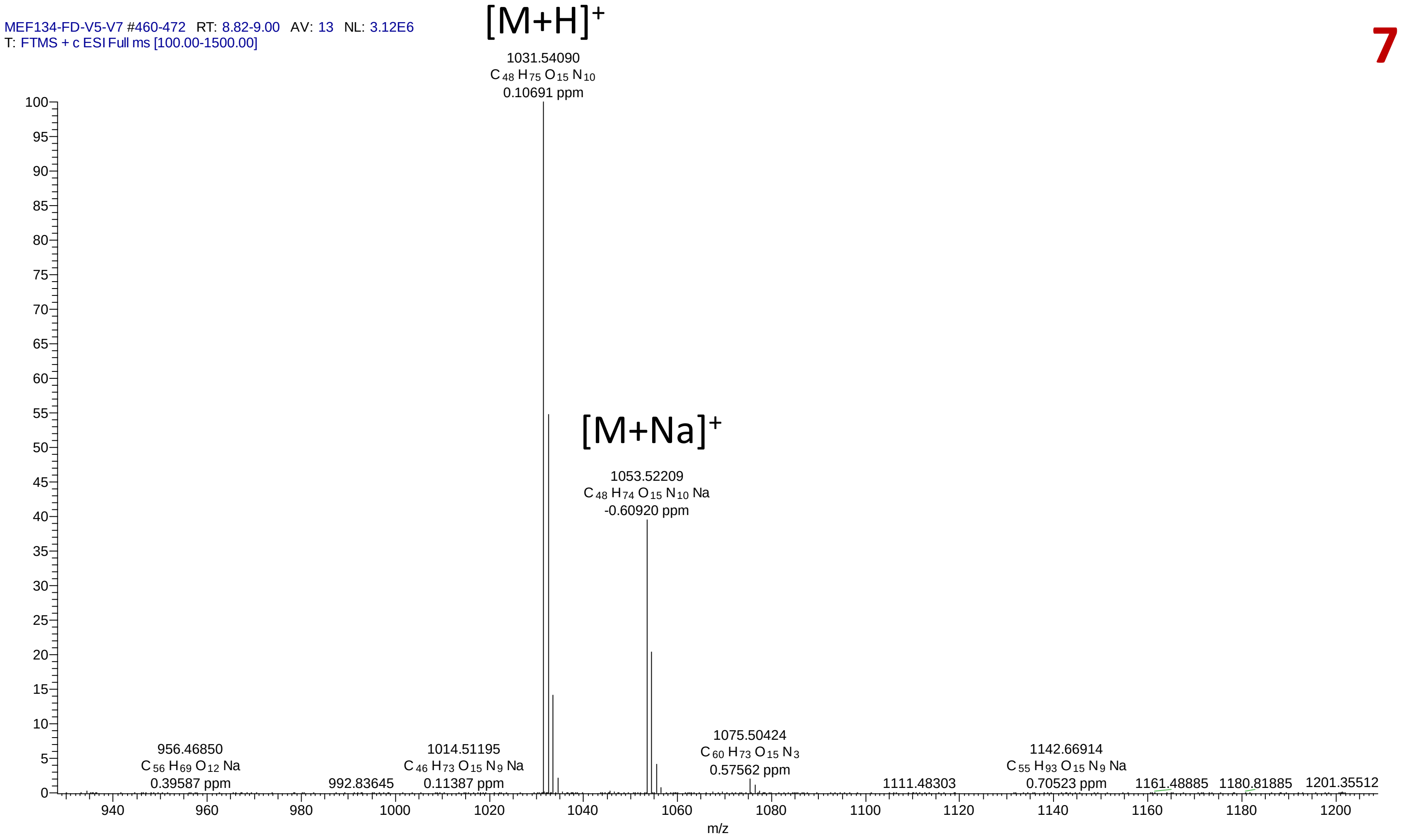

4. MEF134 FD V5 V7
HPLC-HRMS Spectra

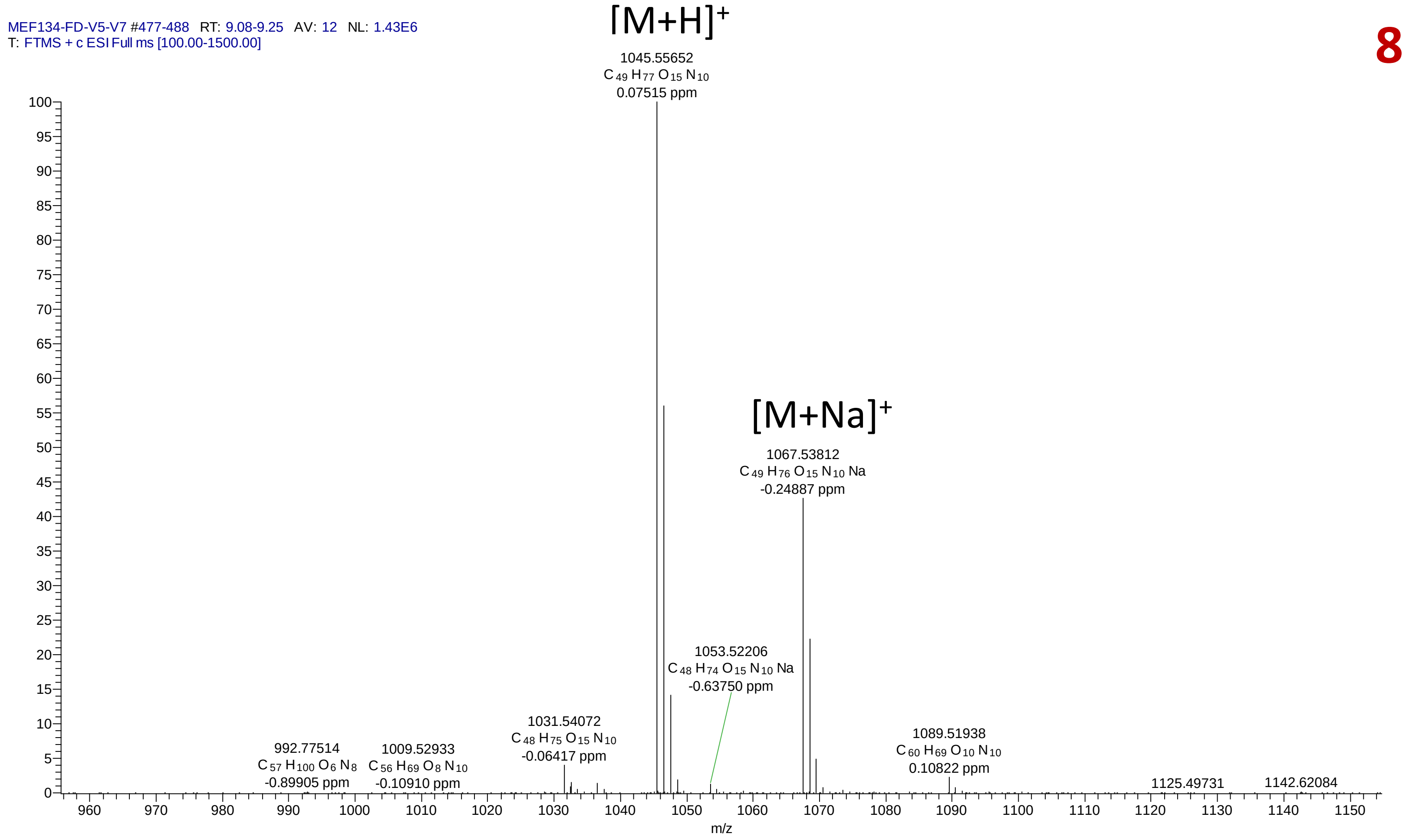

4. MEF134 FD V5 V7 HPLC-HRMS Spectra

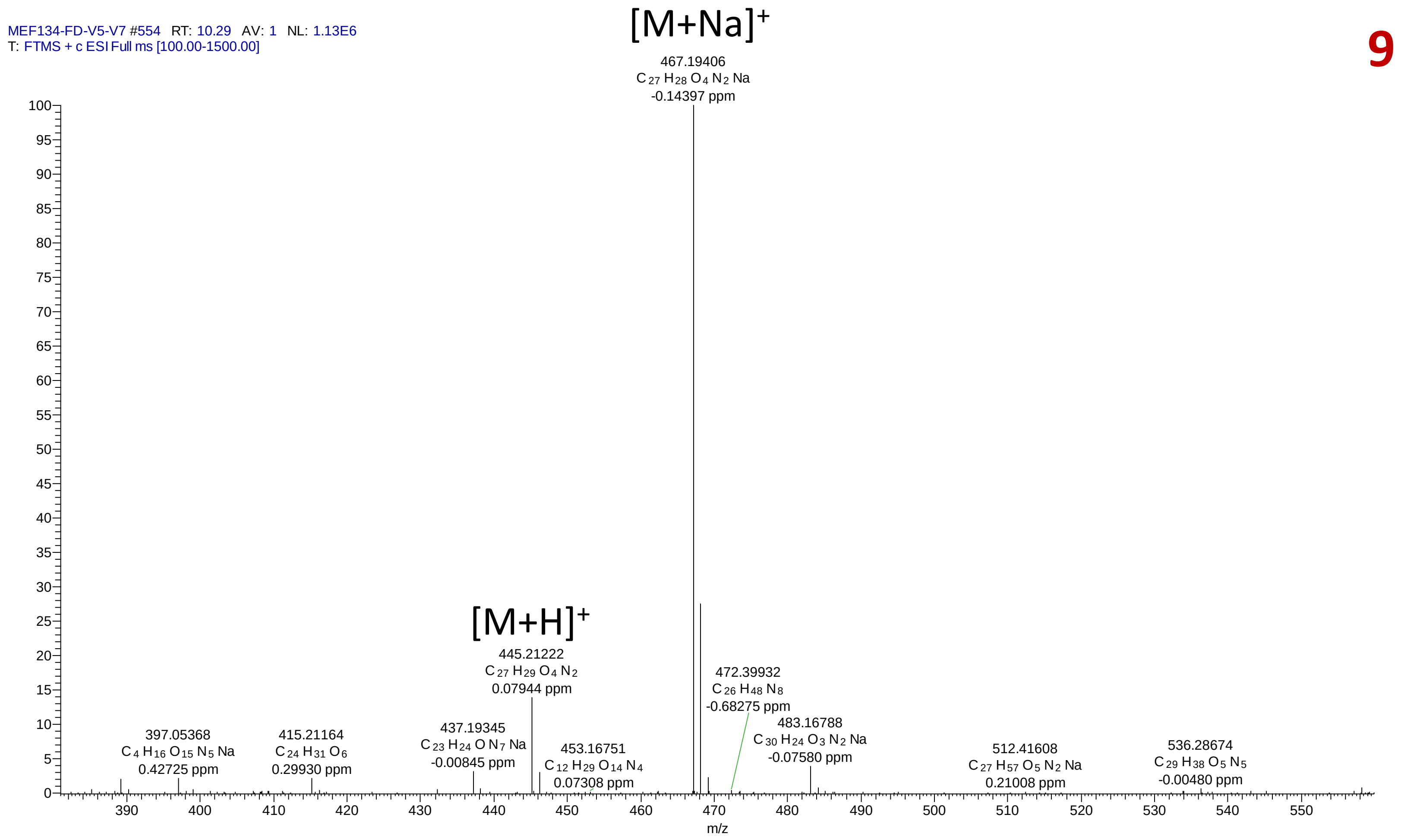

4. MEF134 FD V5 V7 HPLC-HRMS Spectra

10

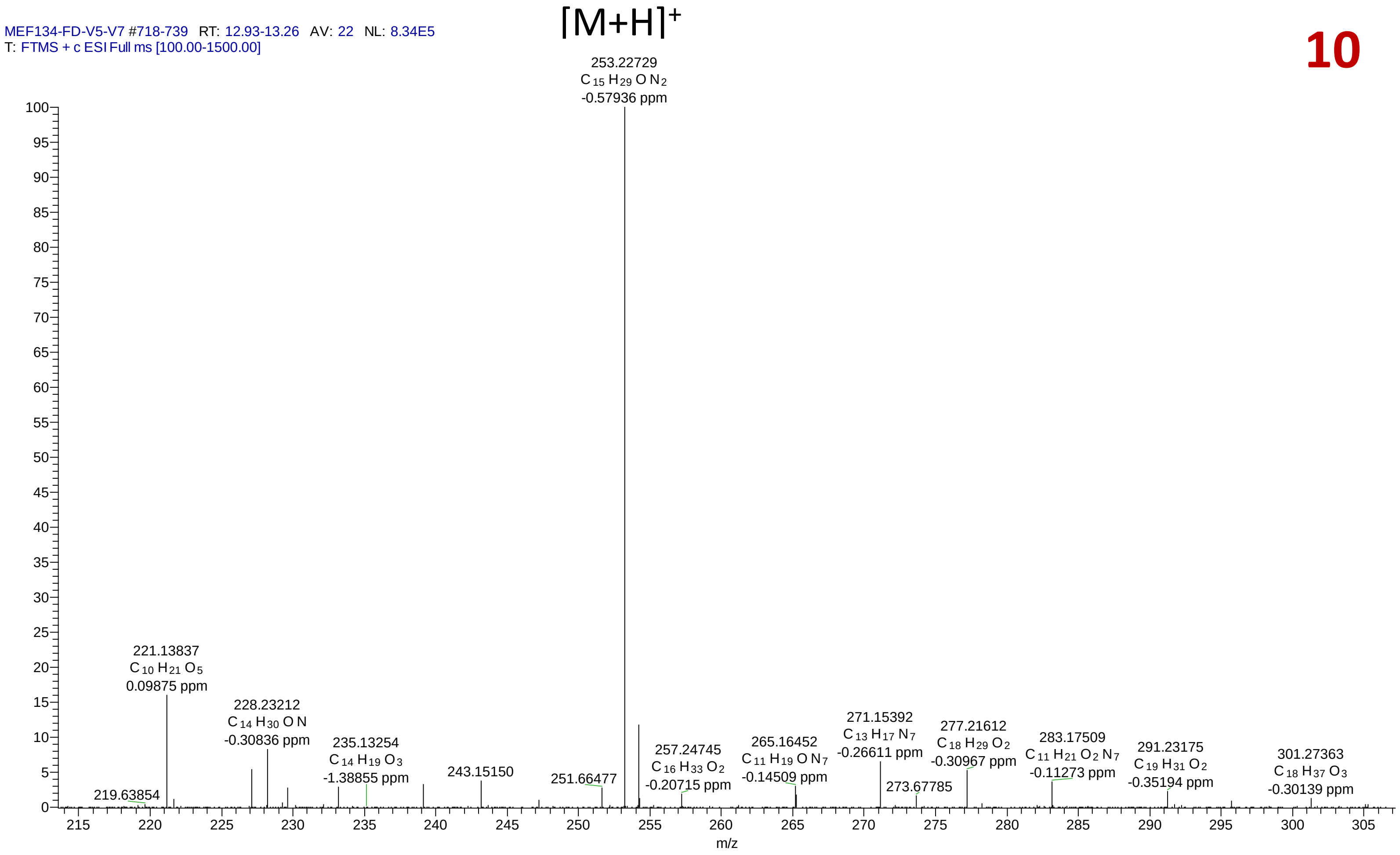

4. MEF134 FD V5 V7 HPLC-HRMS Spectra

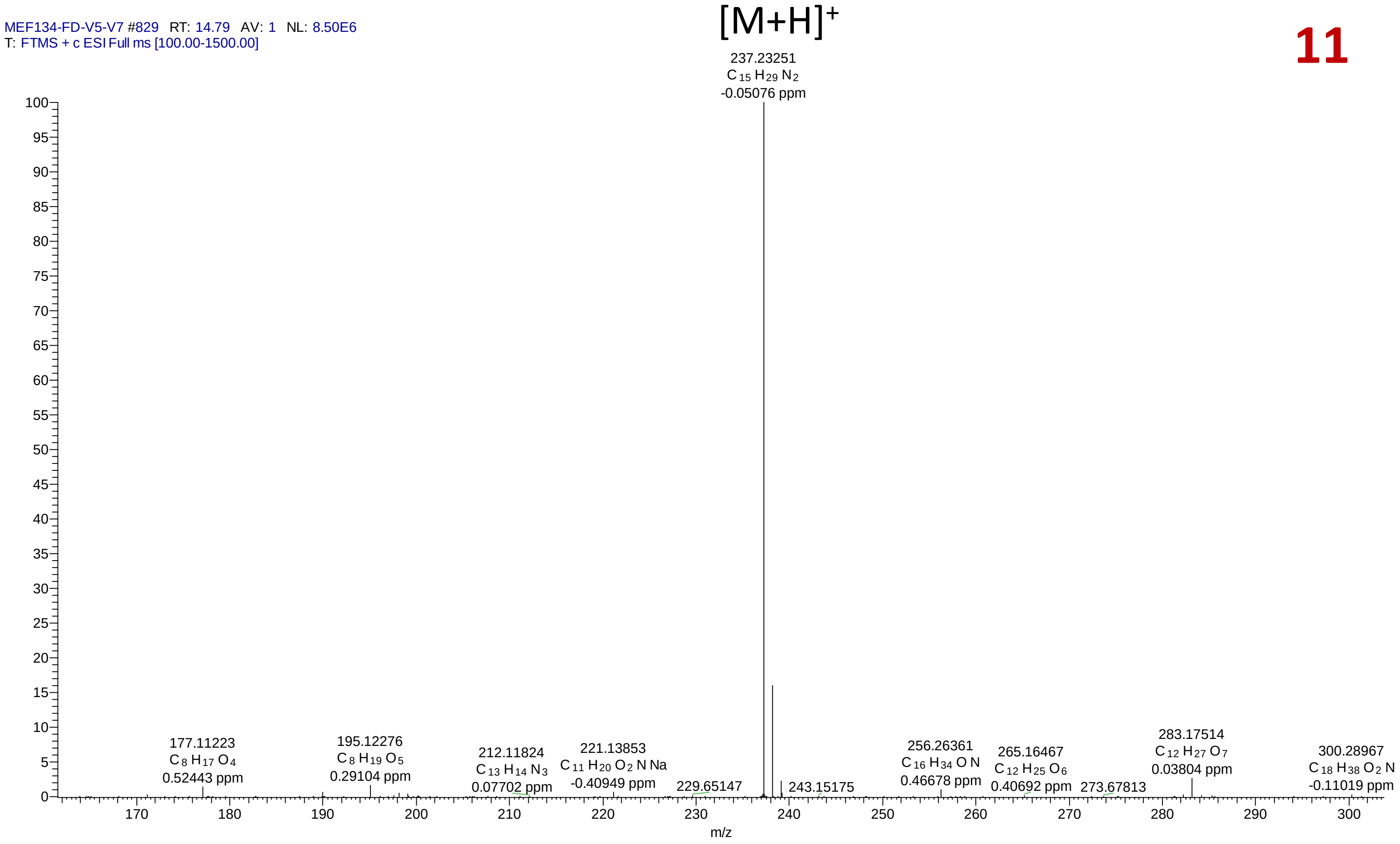

11

5. MEF134 FD V5 V9 HPLC-HRMS Fullscan

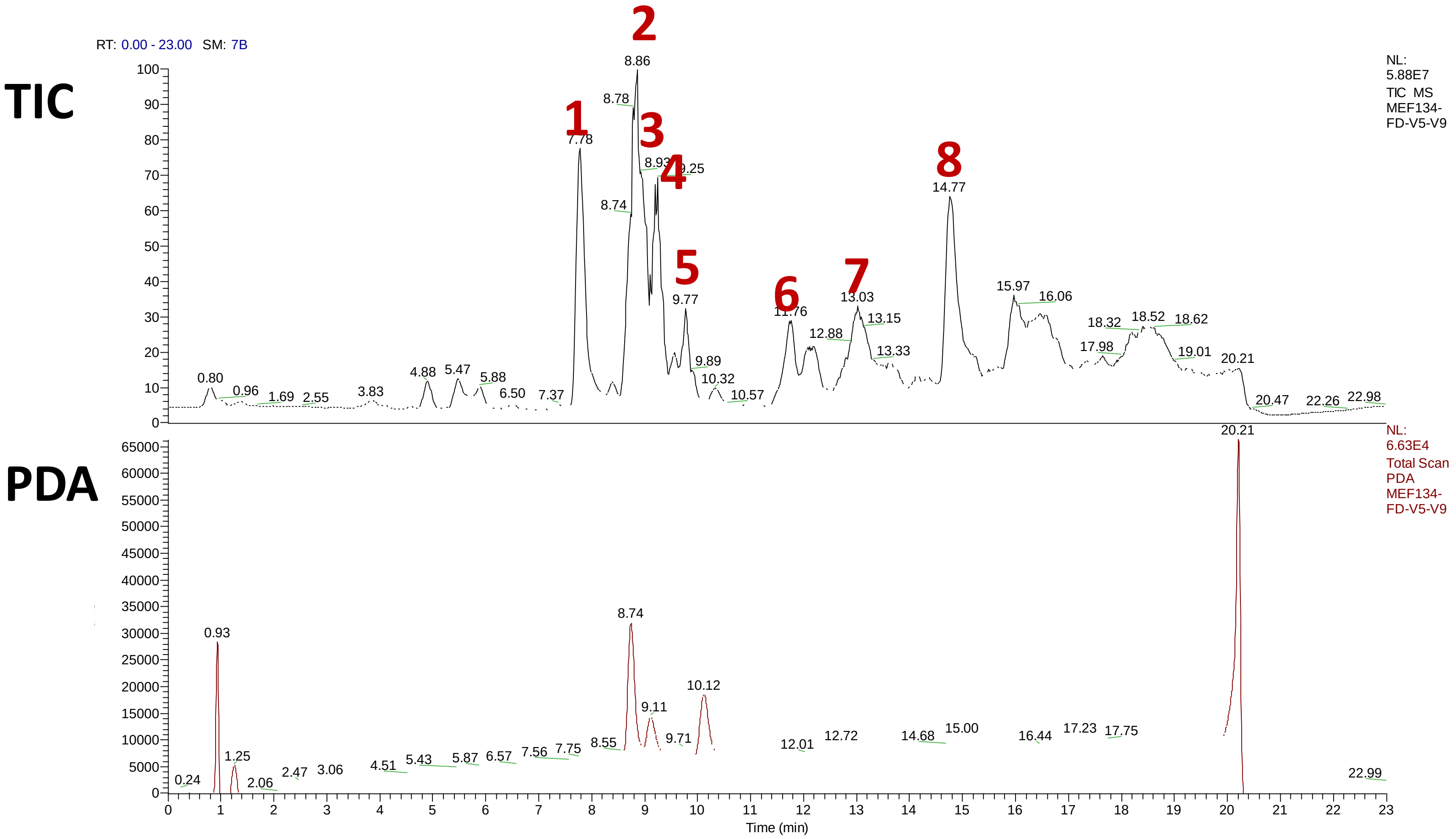

5. MEF134 FD V5 V9 HPLC-HRMS Spectra

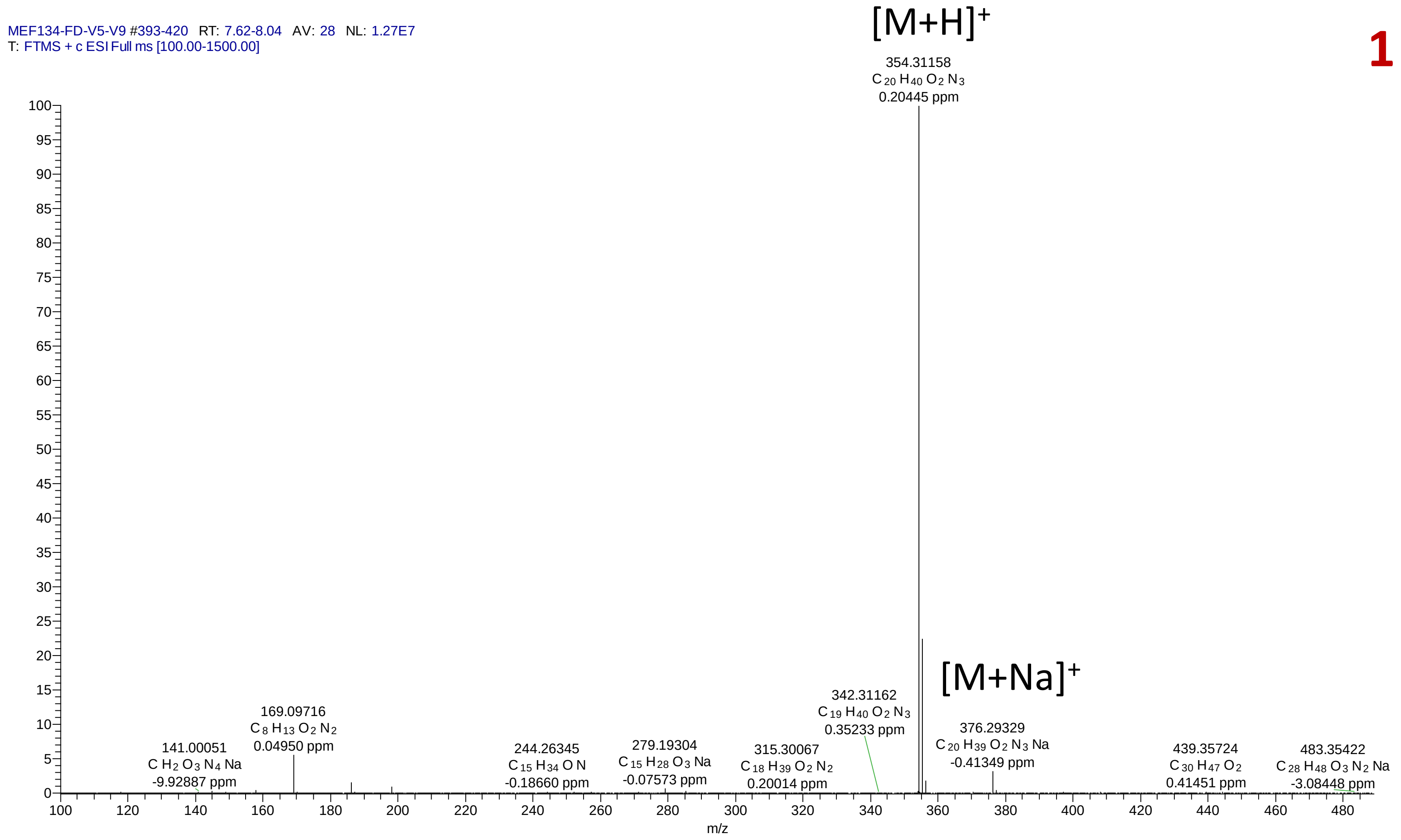

5. MEF134 FD V5 V9 HPLC-HRMS Spectra

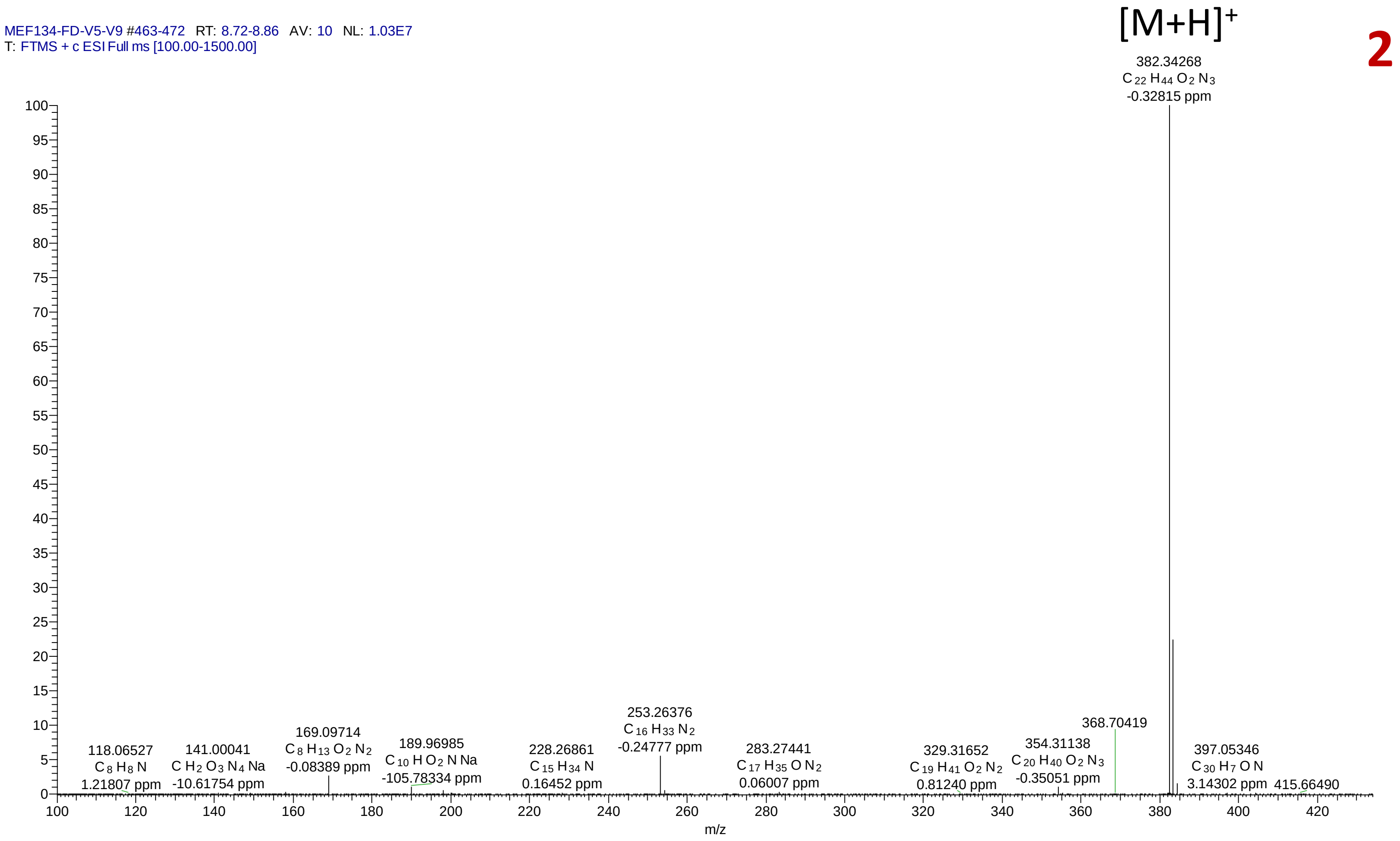

5. MEF134 FD V5 V9 HPLC-HRMS Spectra

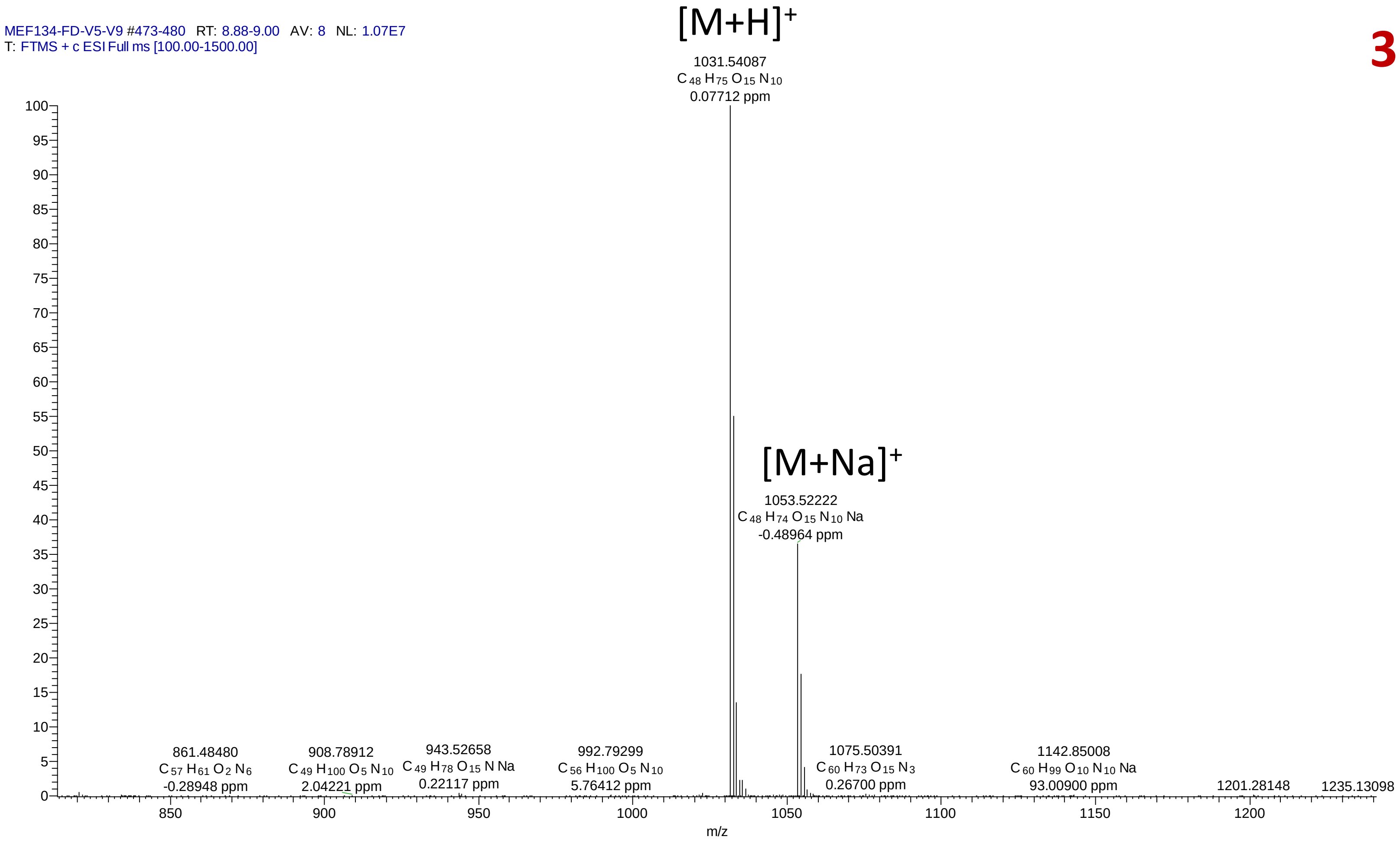

3

5. MEF134 FD V5 V9 HPLC-HRMS Spectra

4

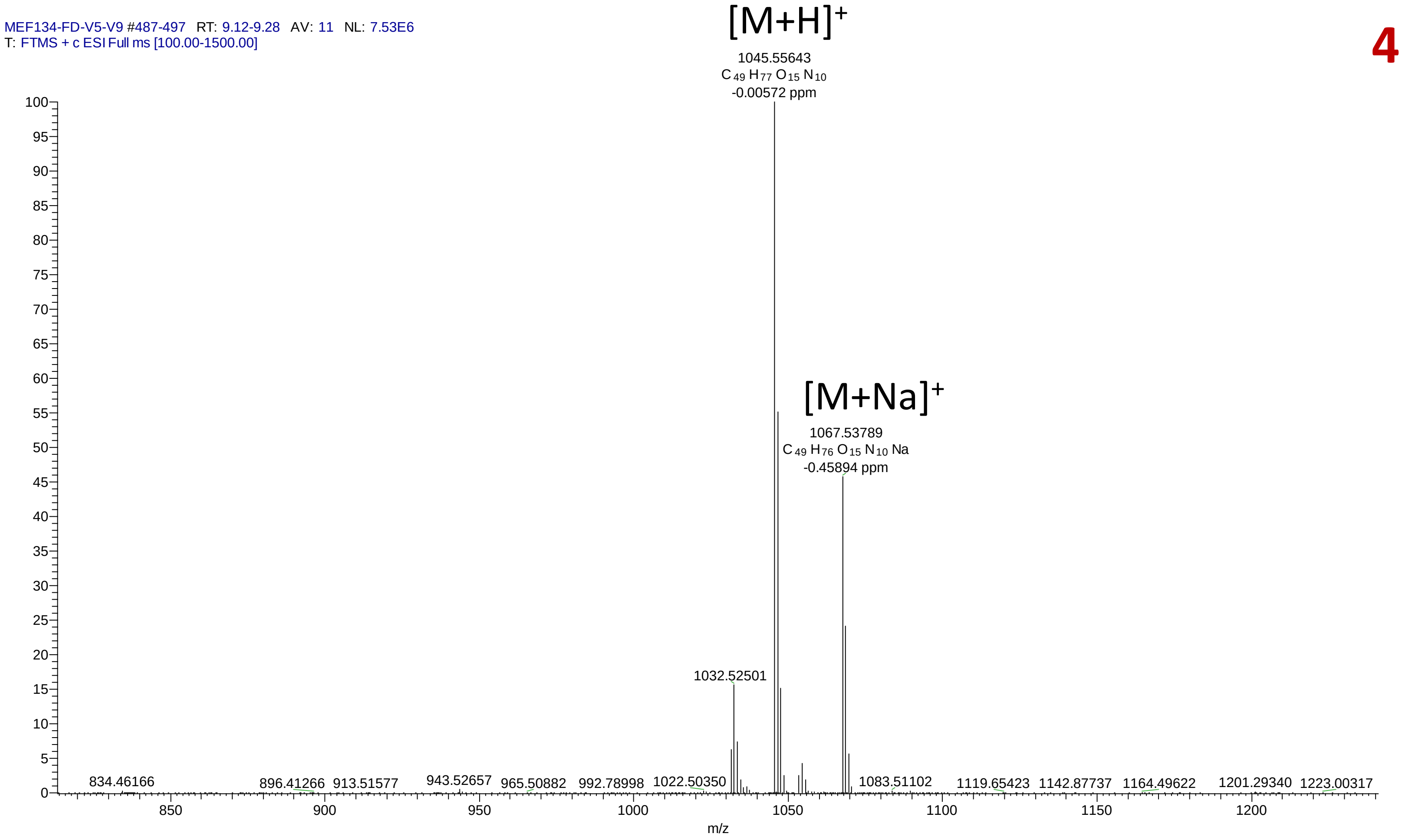

5. MEF134 FD V5 V9 HPLC-HRMS Spectra

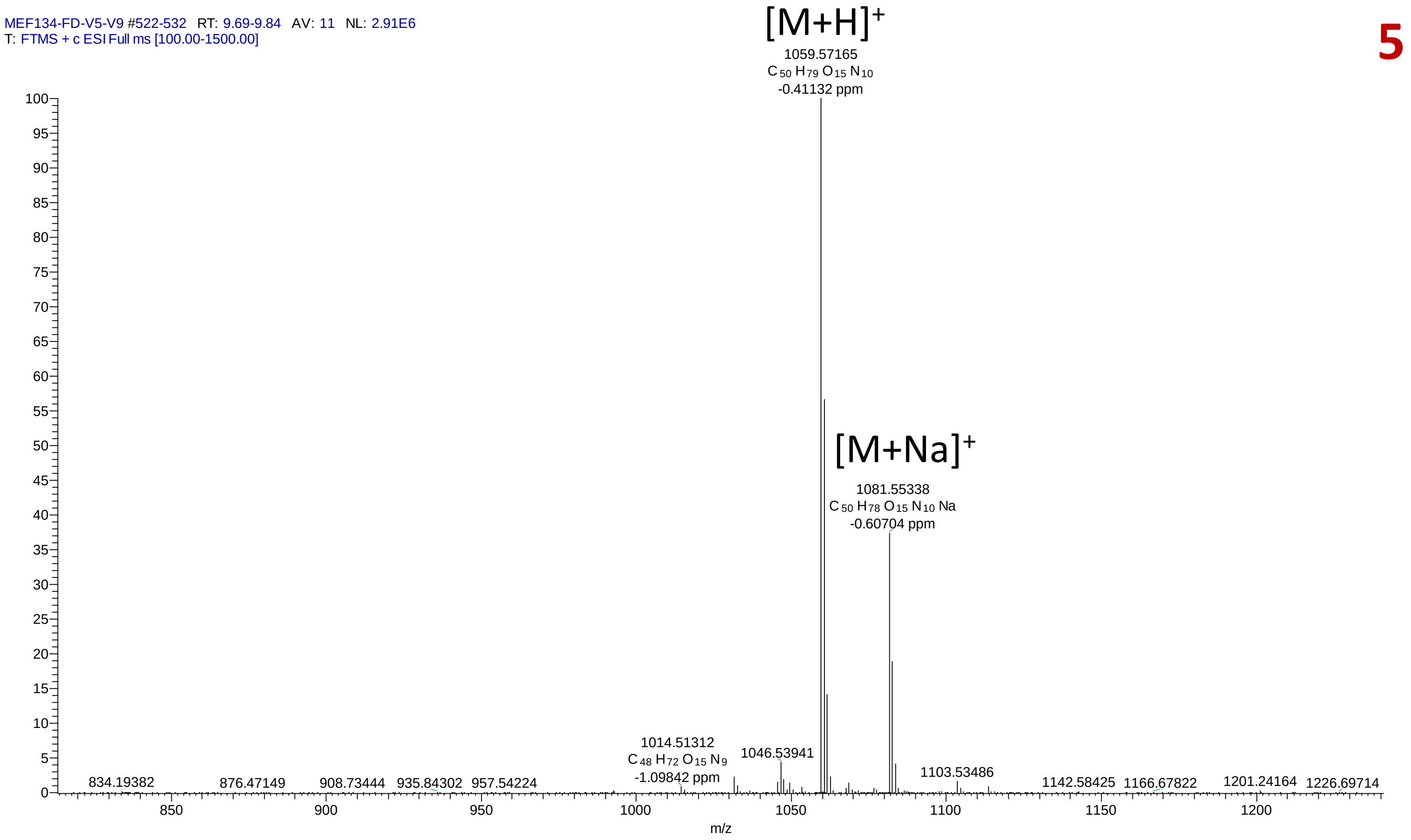

Impurity

5. MEF134 FD V5 V9 HPLC-HRMS Spectra

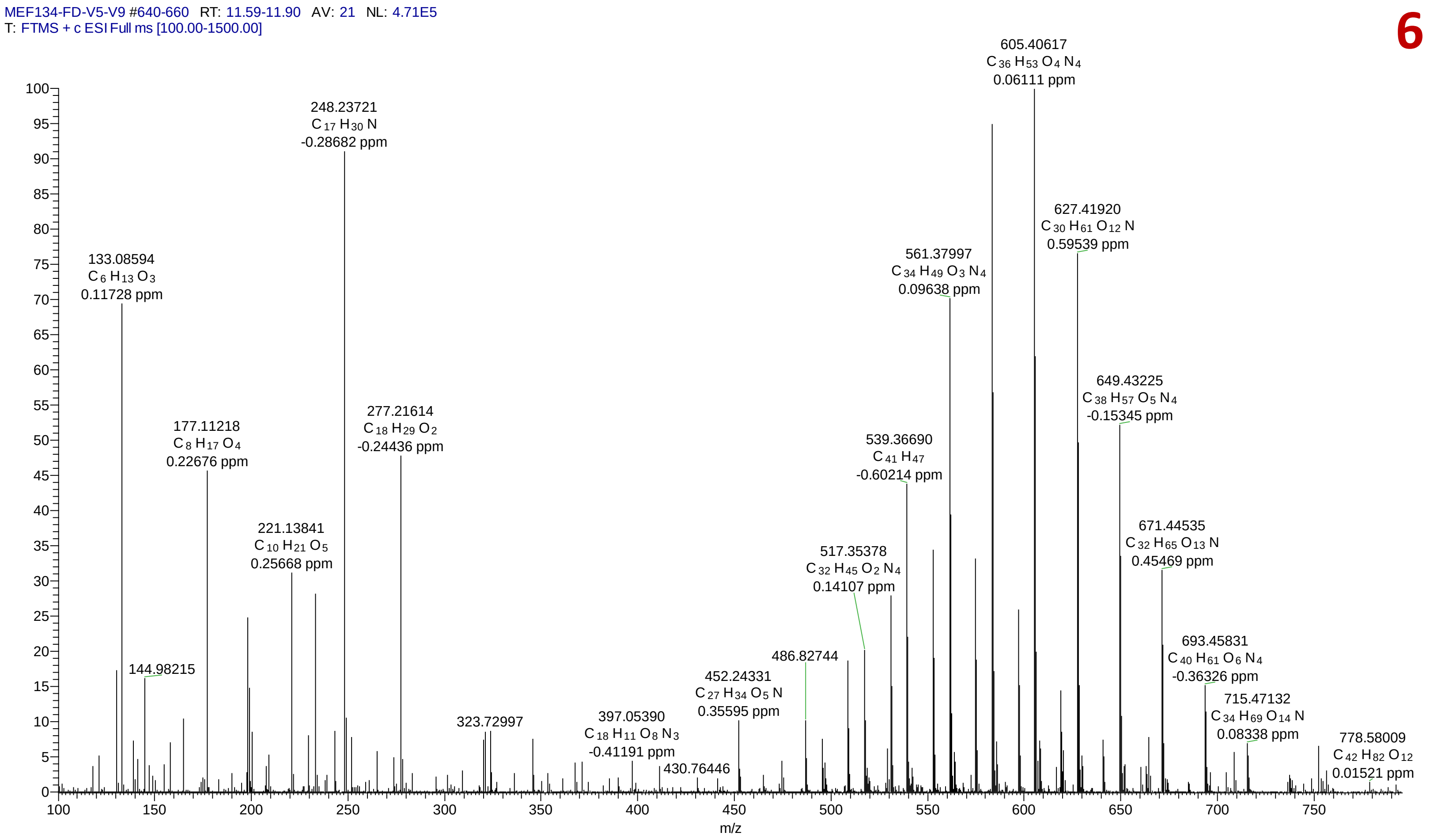

5. MEF134 FD V5 V9 HPLC-HRMS Spectra

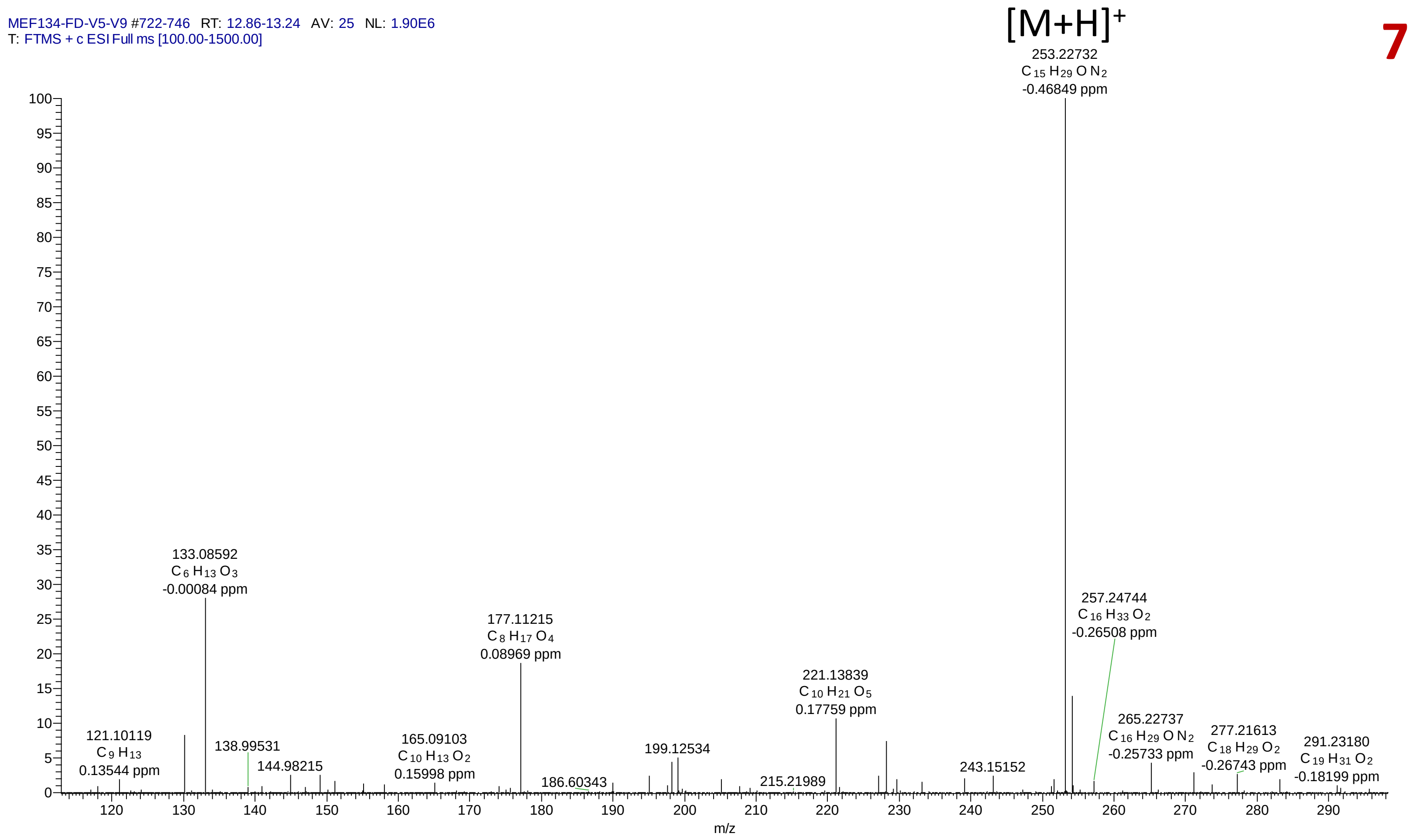

5. MEF134 FD V5 V9 HPLC-HRMS Spectra

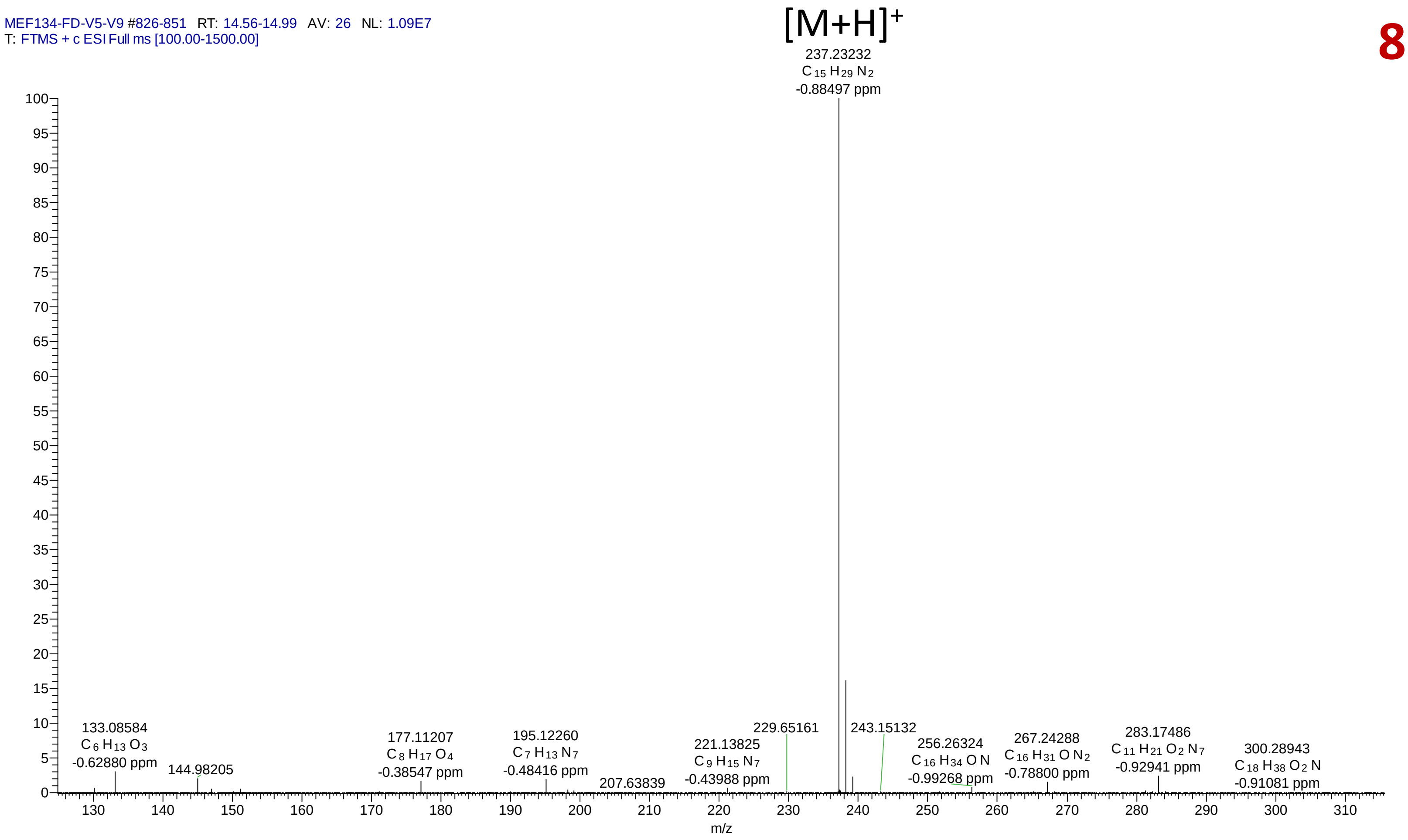

8

6. MEF134 FD K1V1S1-V1 HPLC-HRMS Fullscan

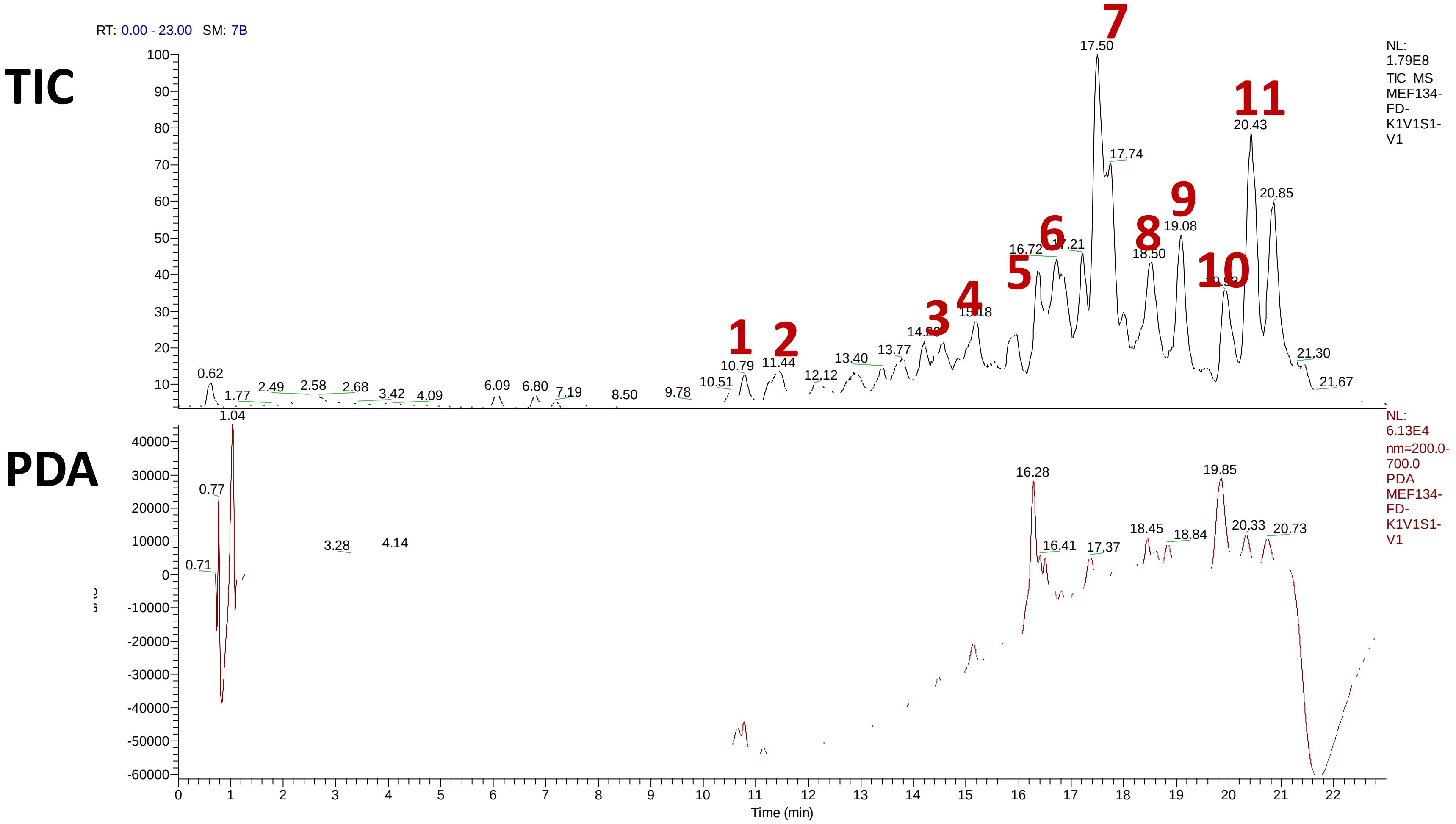

6. MEF134 FD K1V1S1-V1 HPLC-HRMS Spectra

1

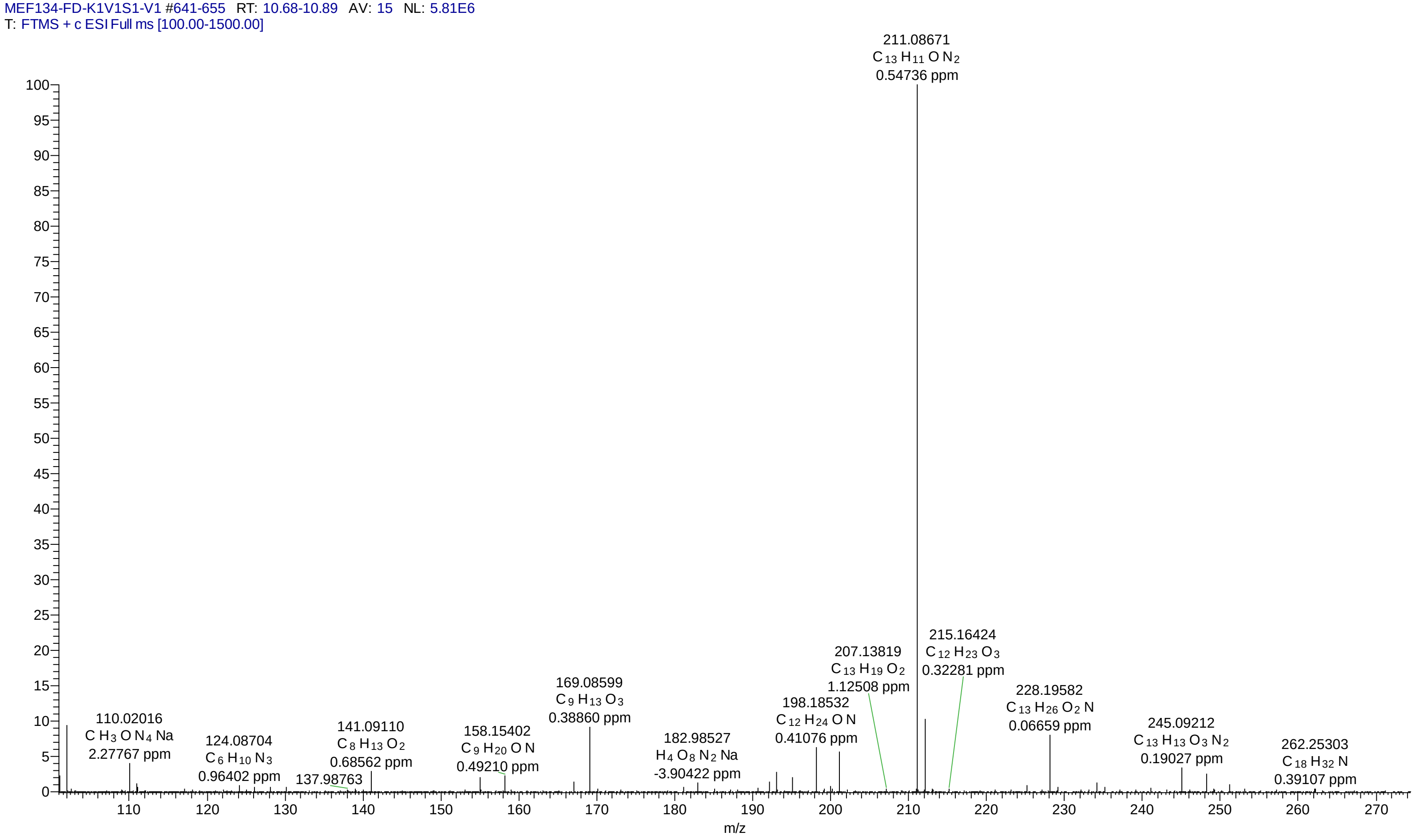

6. MEF134 FD K1V1S1-V1 HPLC-HRMS Spectra

2

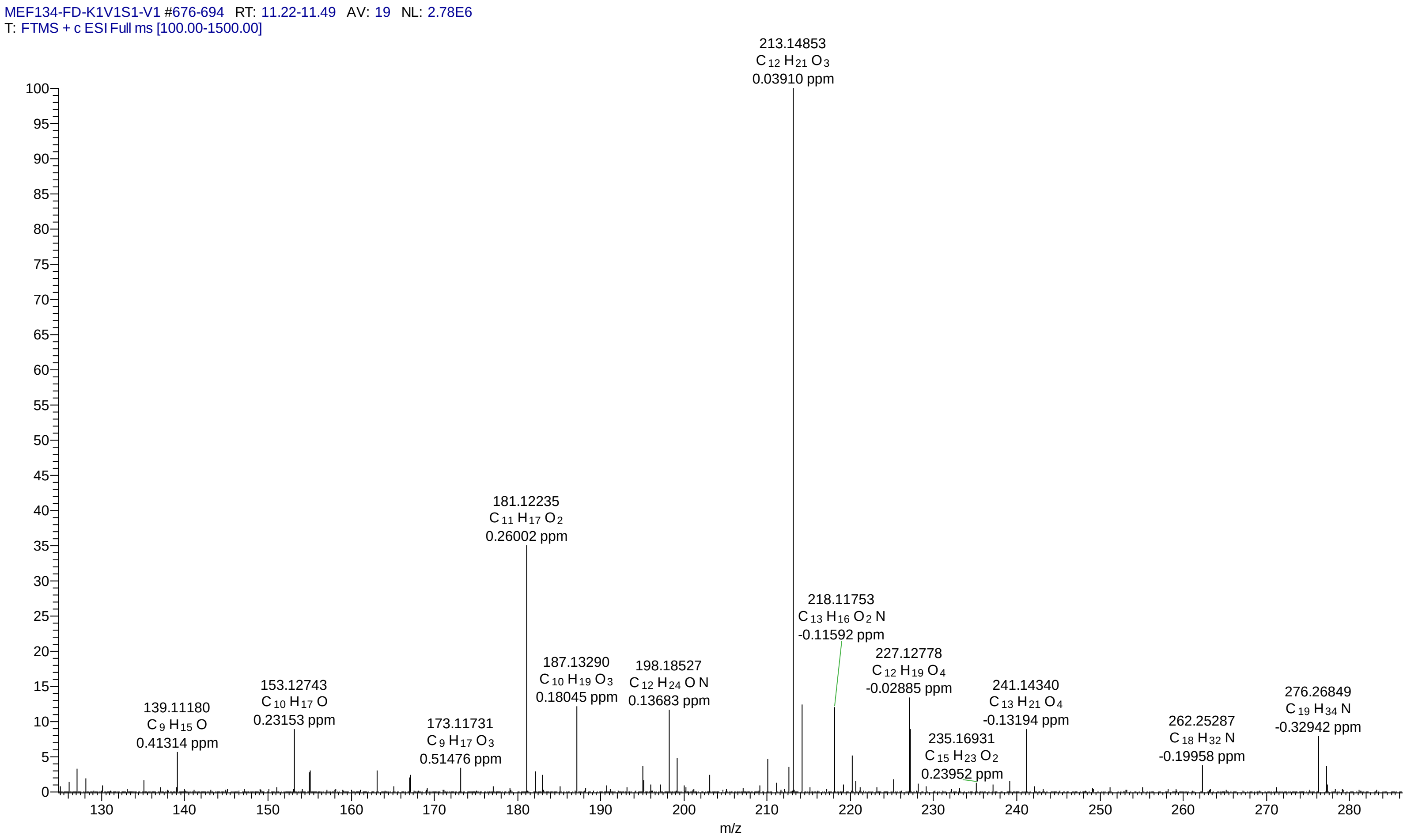

6. MEF134 FD K1V1S1-V1 HPLC-HRMS Spectra

3

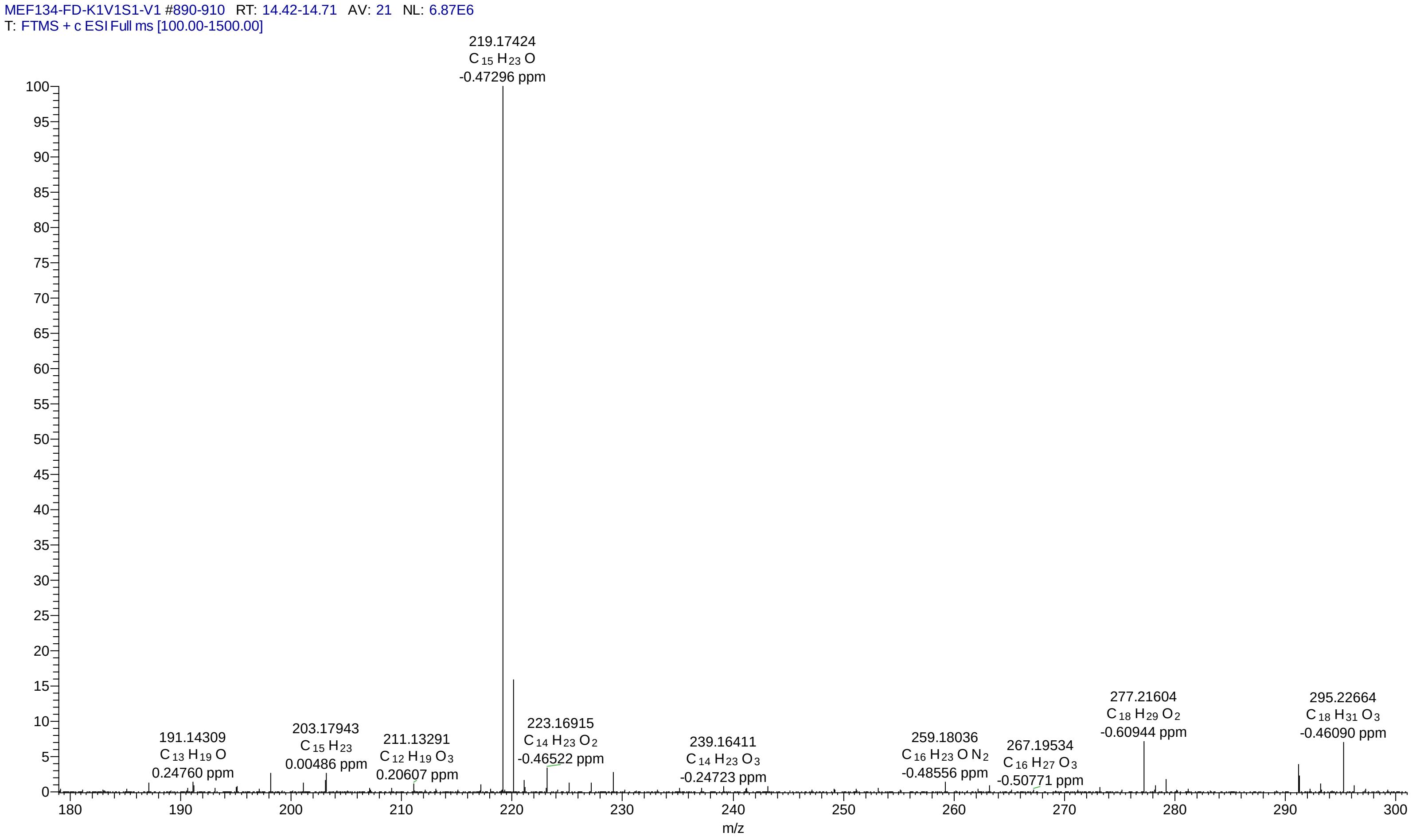

6. MEF134 FD K1V1S1-V1 HPLC-HRMS Spectra

4

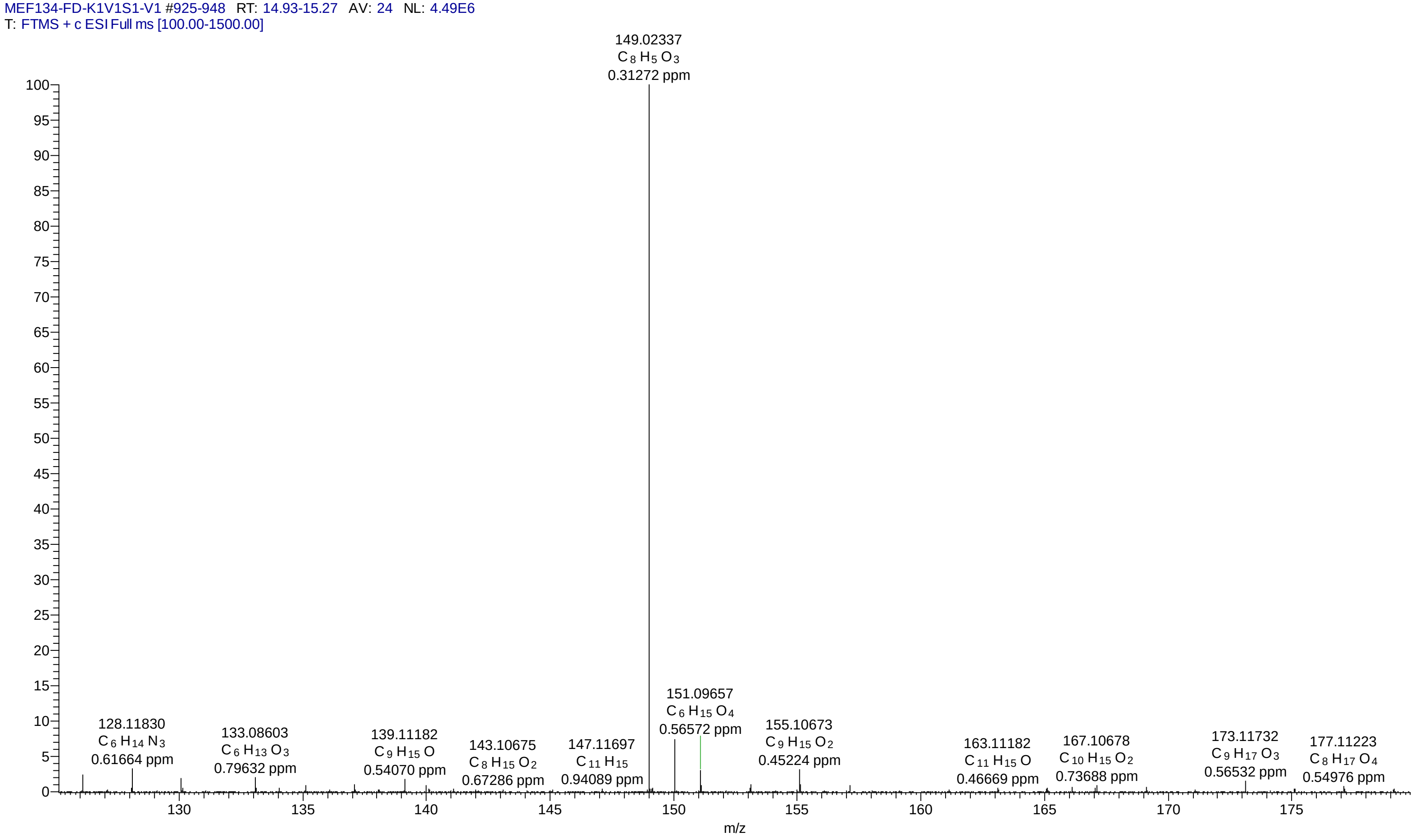

6. MEF134 FD K1V1S1-V1 HPLC-HRMS Spectra

5

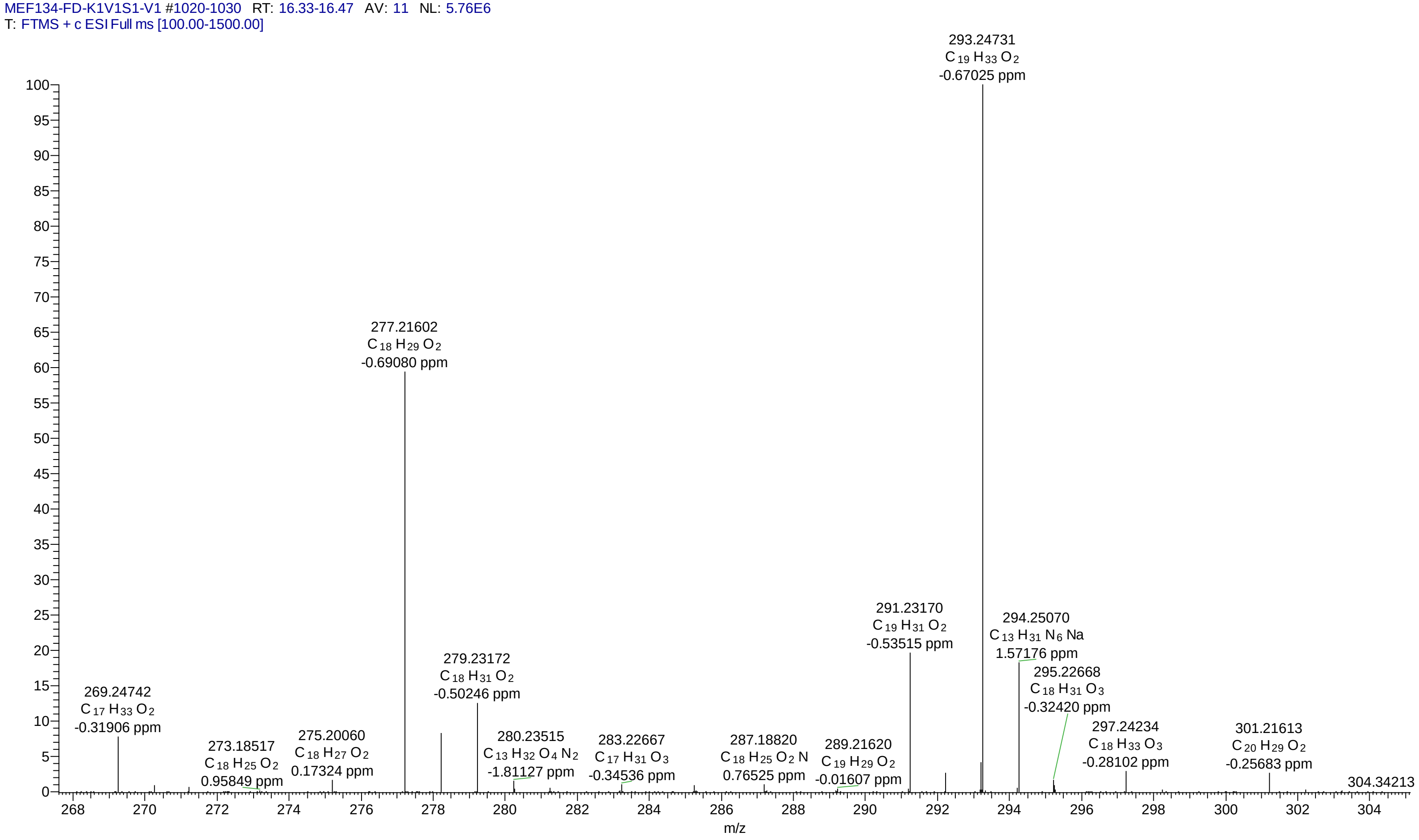

6. MEF134 FD K1V1S1-V1 HPLC-HRMS Spectra

6

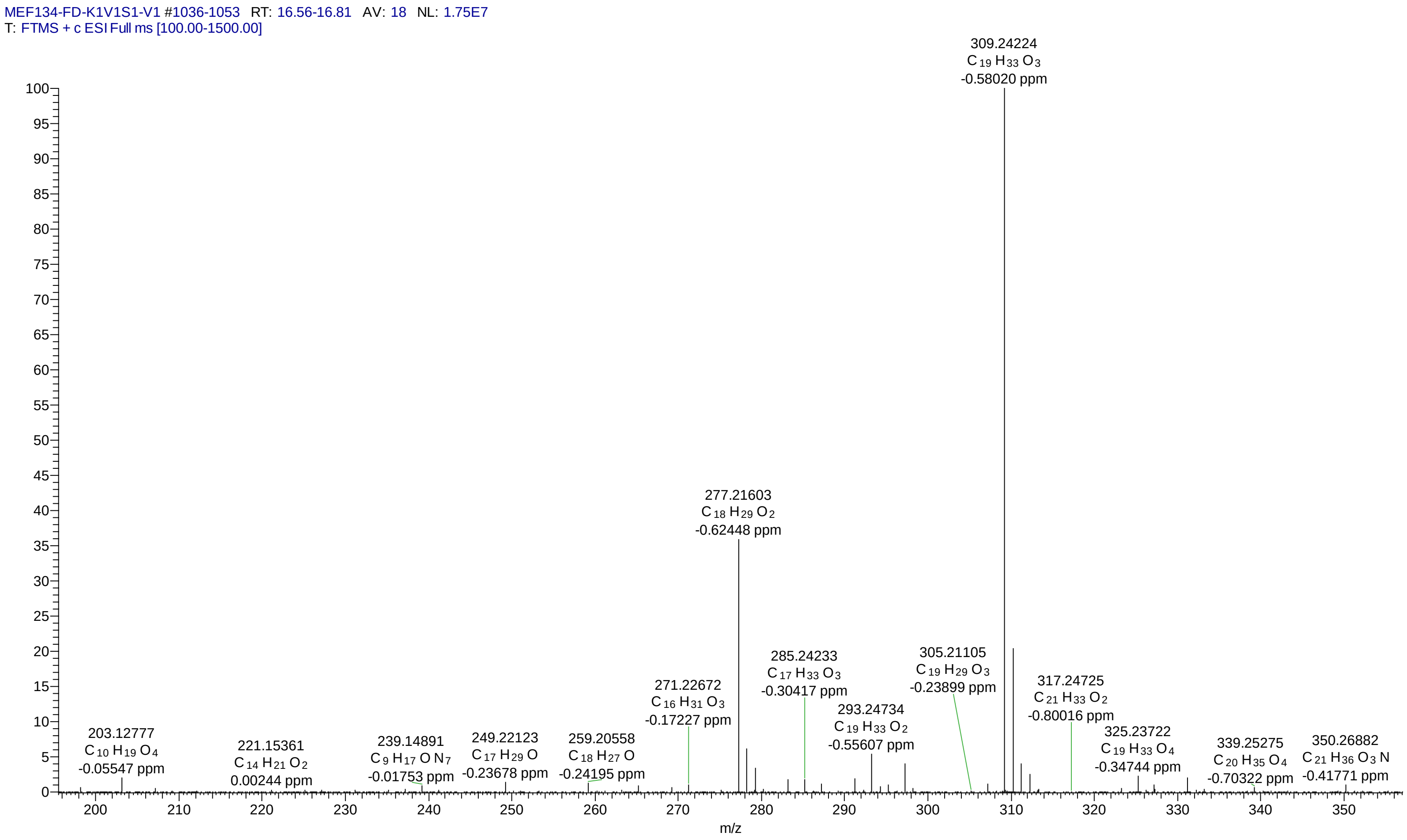

6. MEF134 FD K1V1S1-V1 HPLC-HRMS Spectra

7

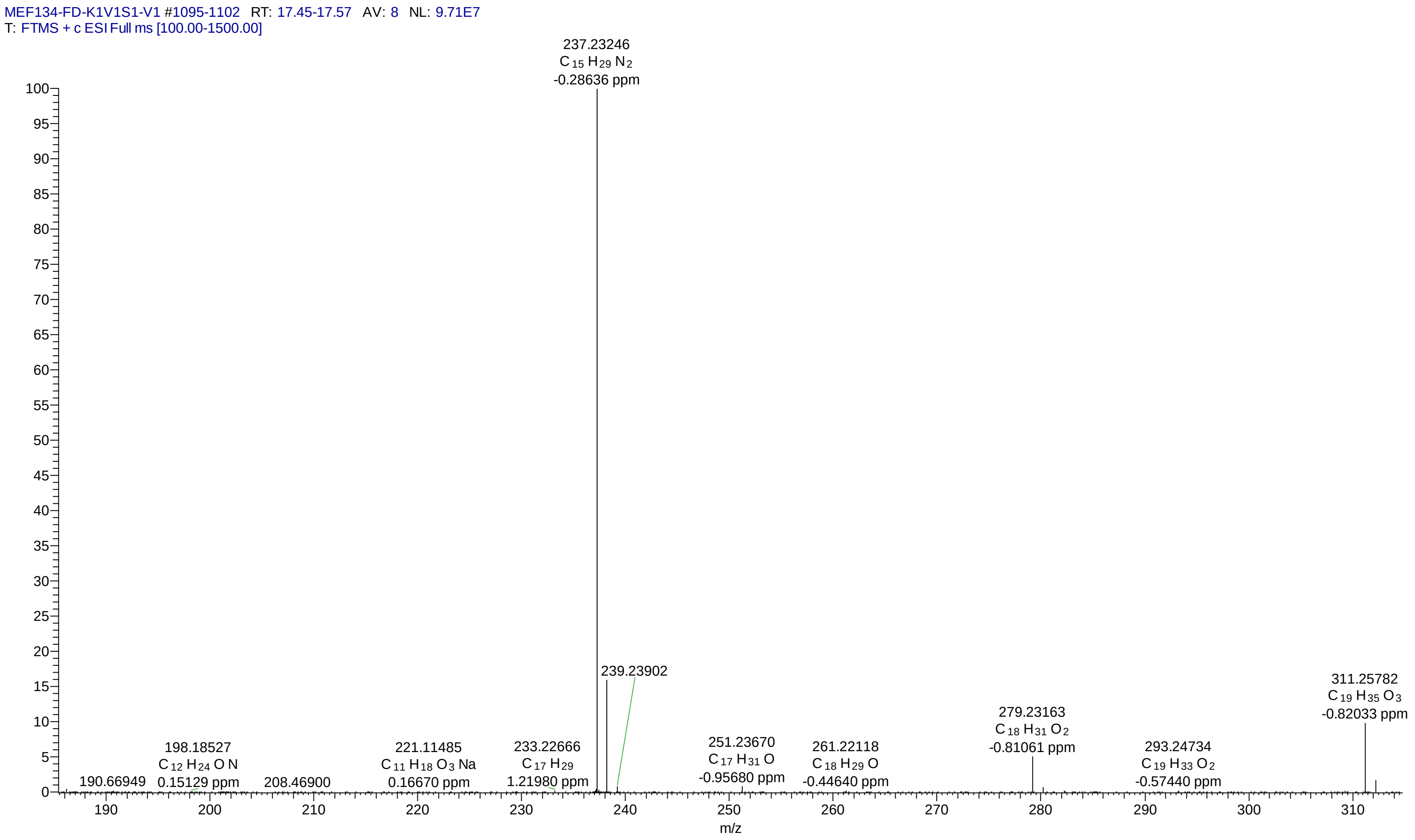

6. MEF134 FD K1V1S1-V1 HPLC-HRMS Spectra

8

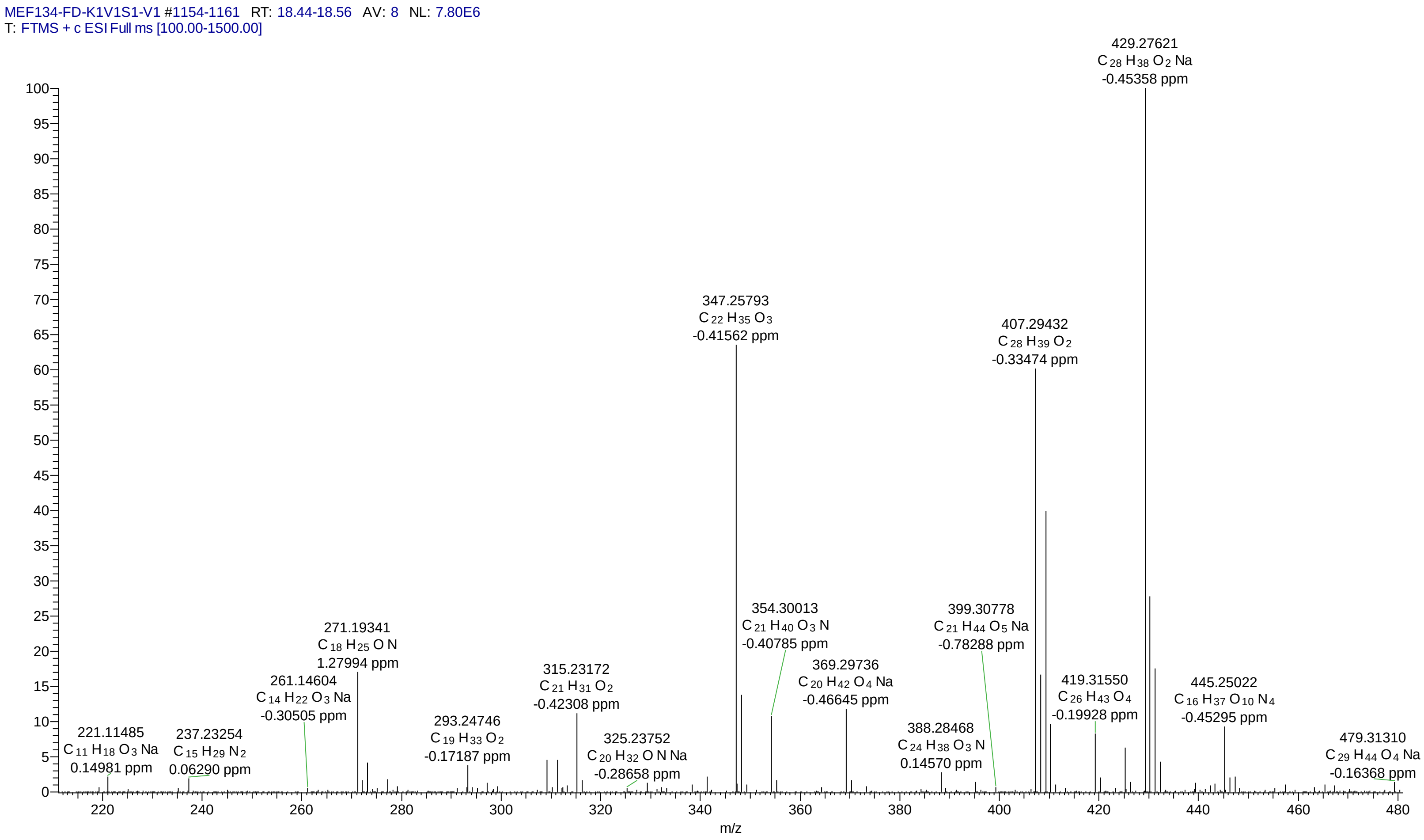

6. MEF134 FD K1V1S1-V1 HPLC-HRMS Spectra

10

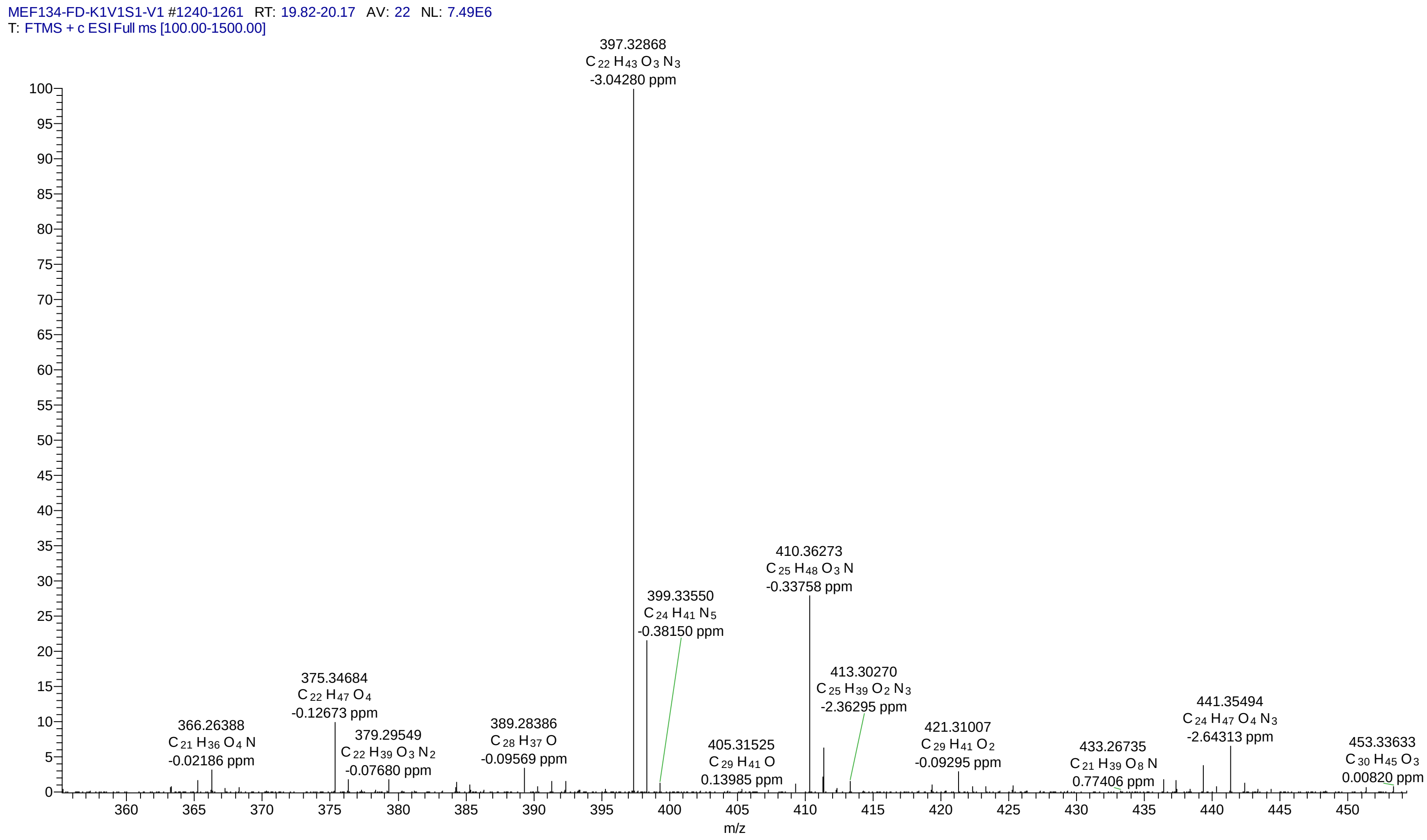

6. MEF134 FD K1V1S1-V1 HPLC-HRMS Spectra

11

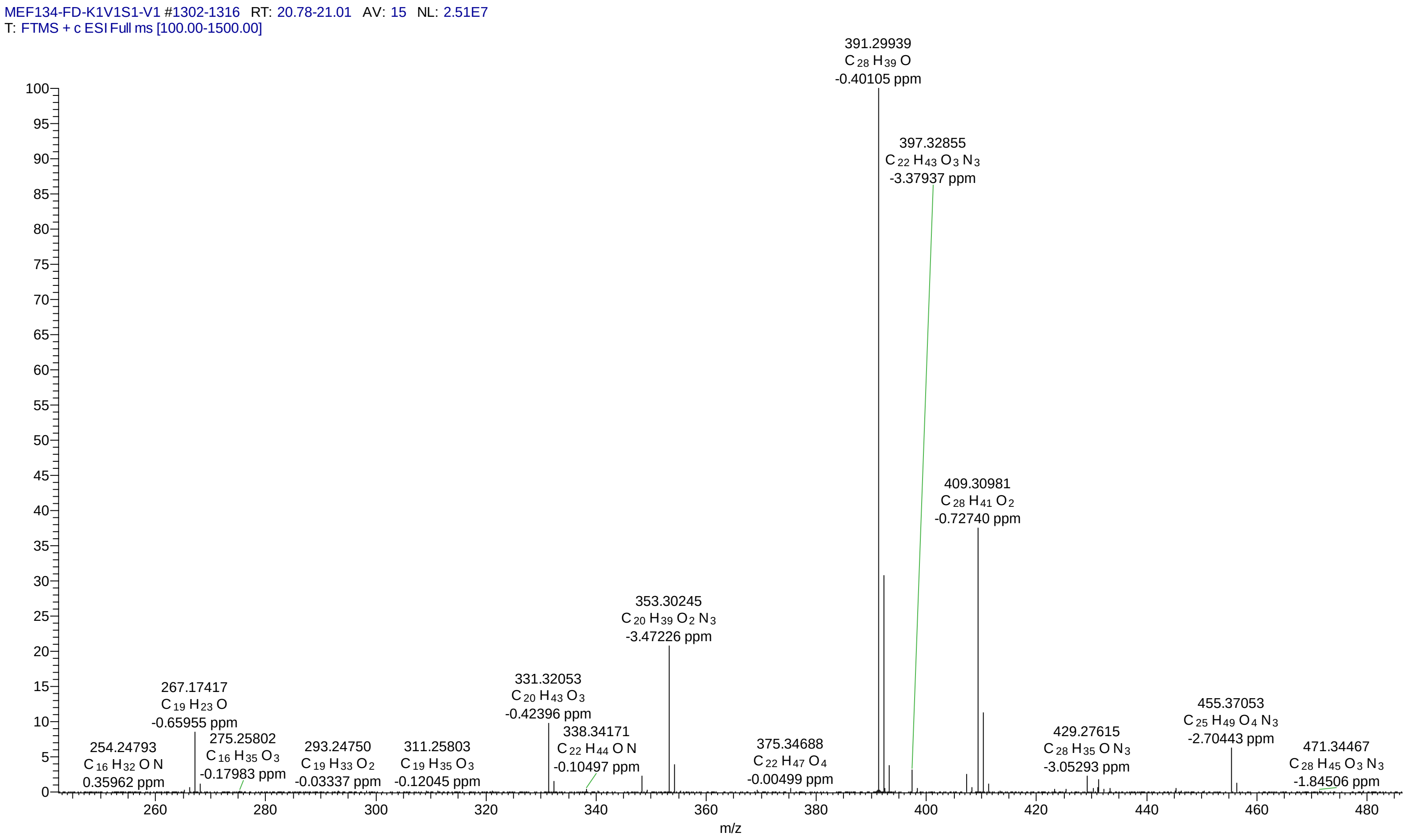

7. MEF134 FD K1V5S9+K1V5S10-V3 HPLC-HRMS Fullscan

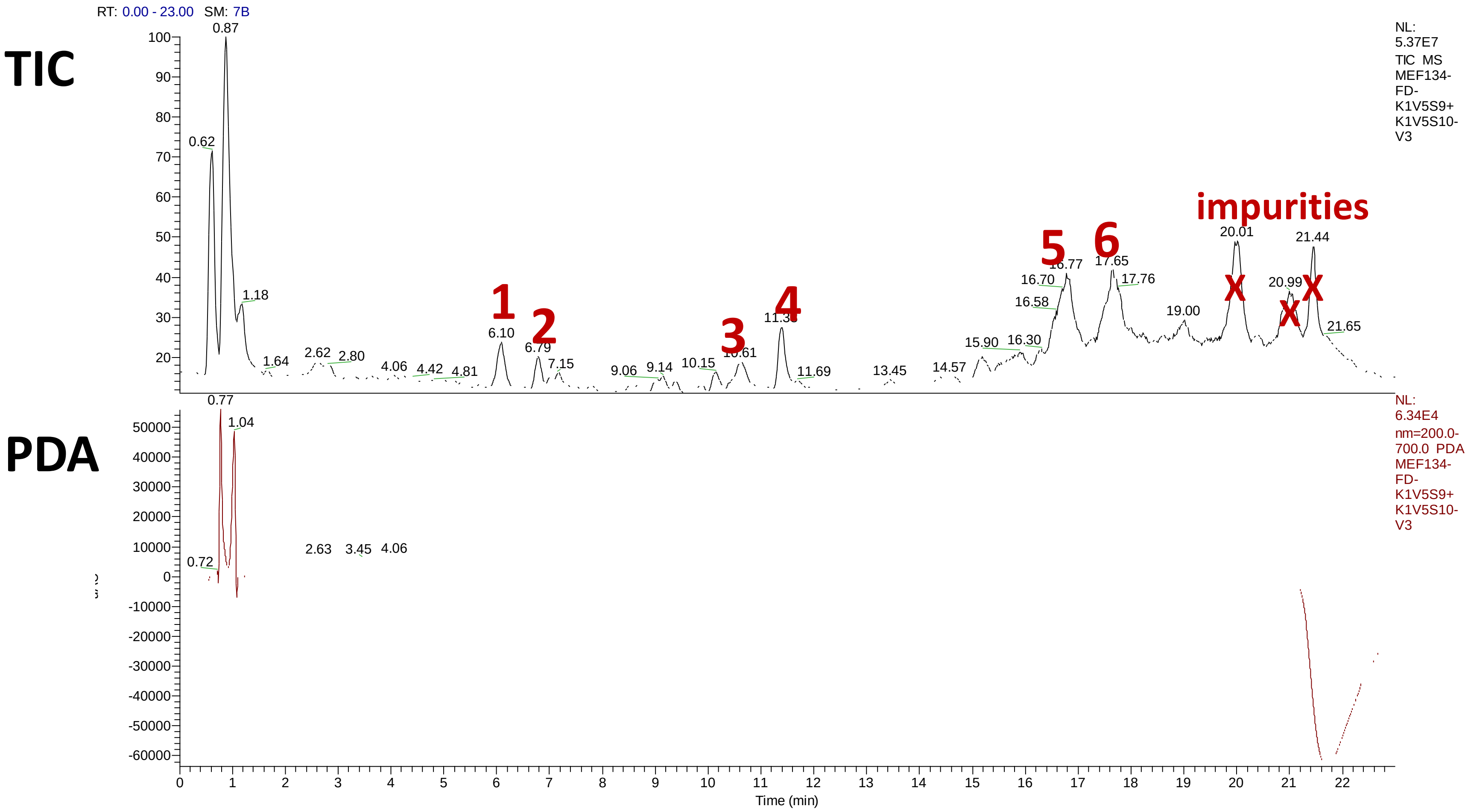

7. MEF134 FD K1V5S9+K1V5S10-V3 HPLC-HRMS Spectra

1

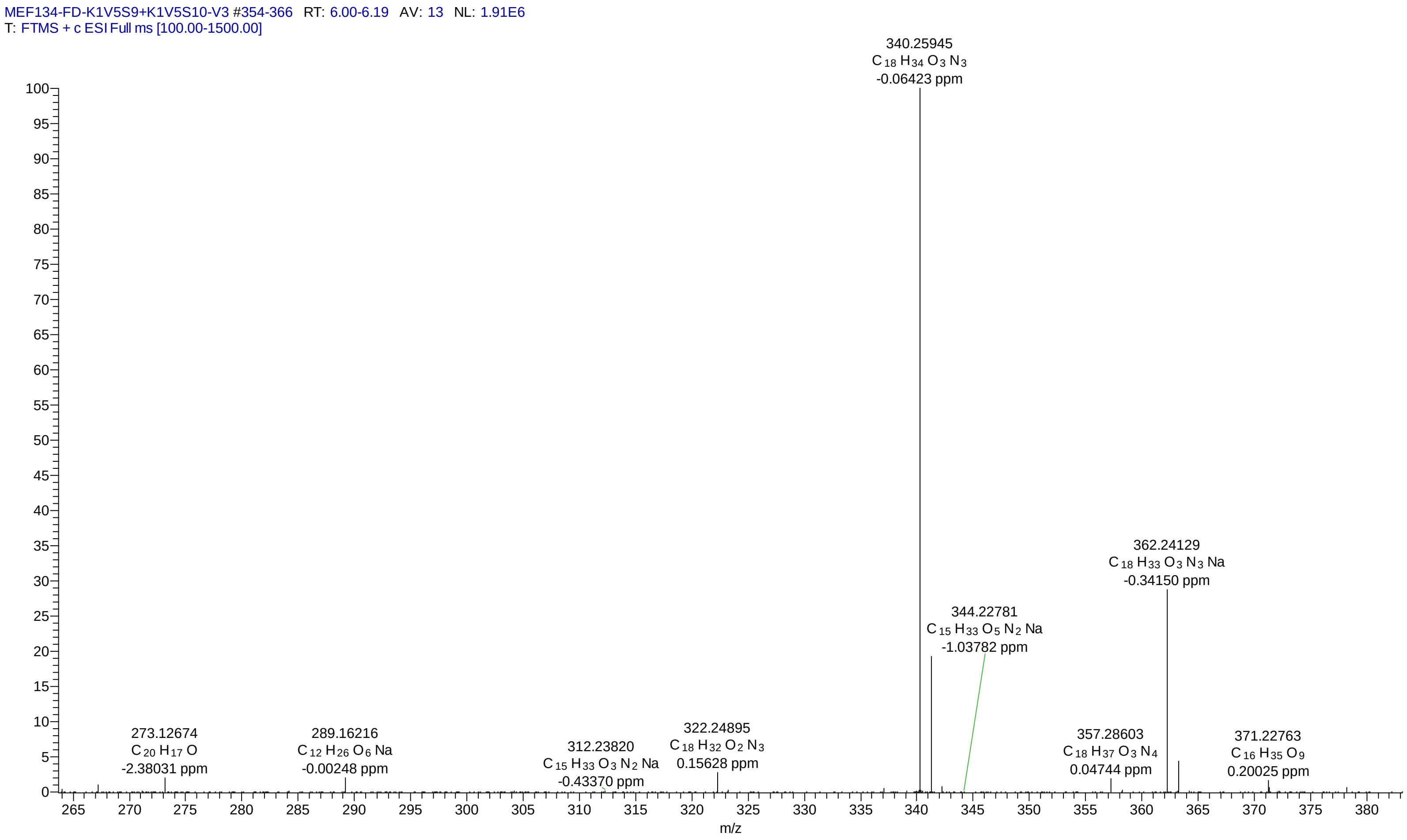

7. MEF134 FD K1V5S9+K1V5S10-V3 HPLC-HRMS Spectra

2

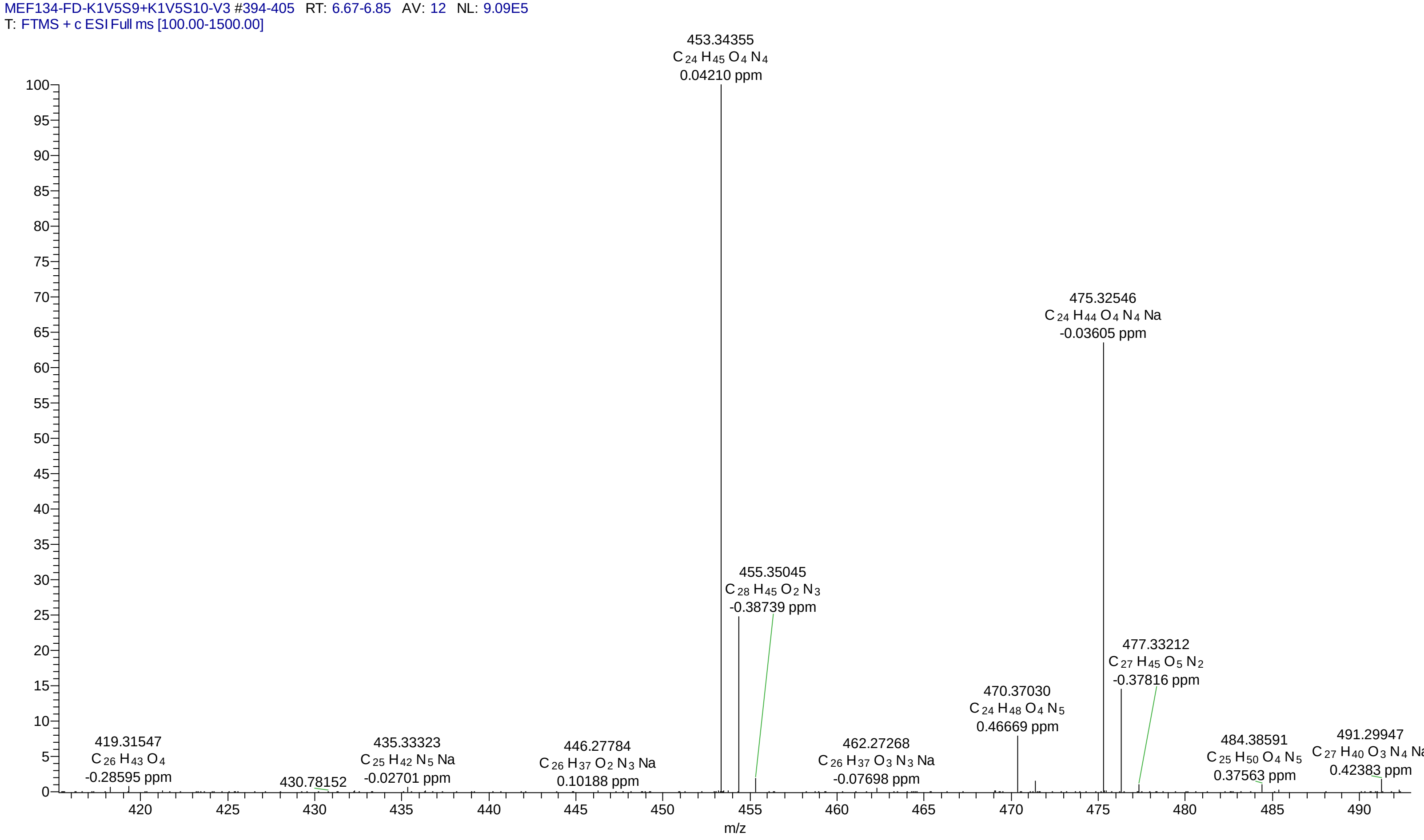

7. MEF134 FD K1V5S9+K1V5S10-V3 HPLC-HRMS Spectra

3

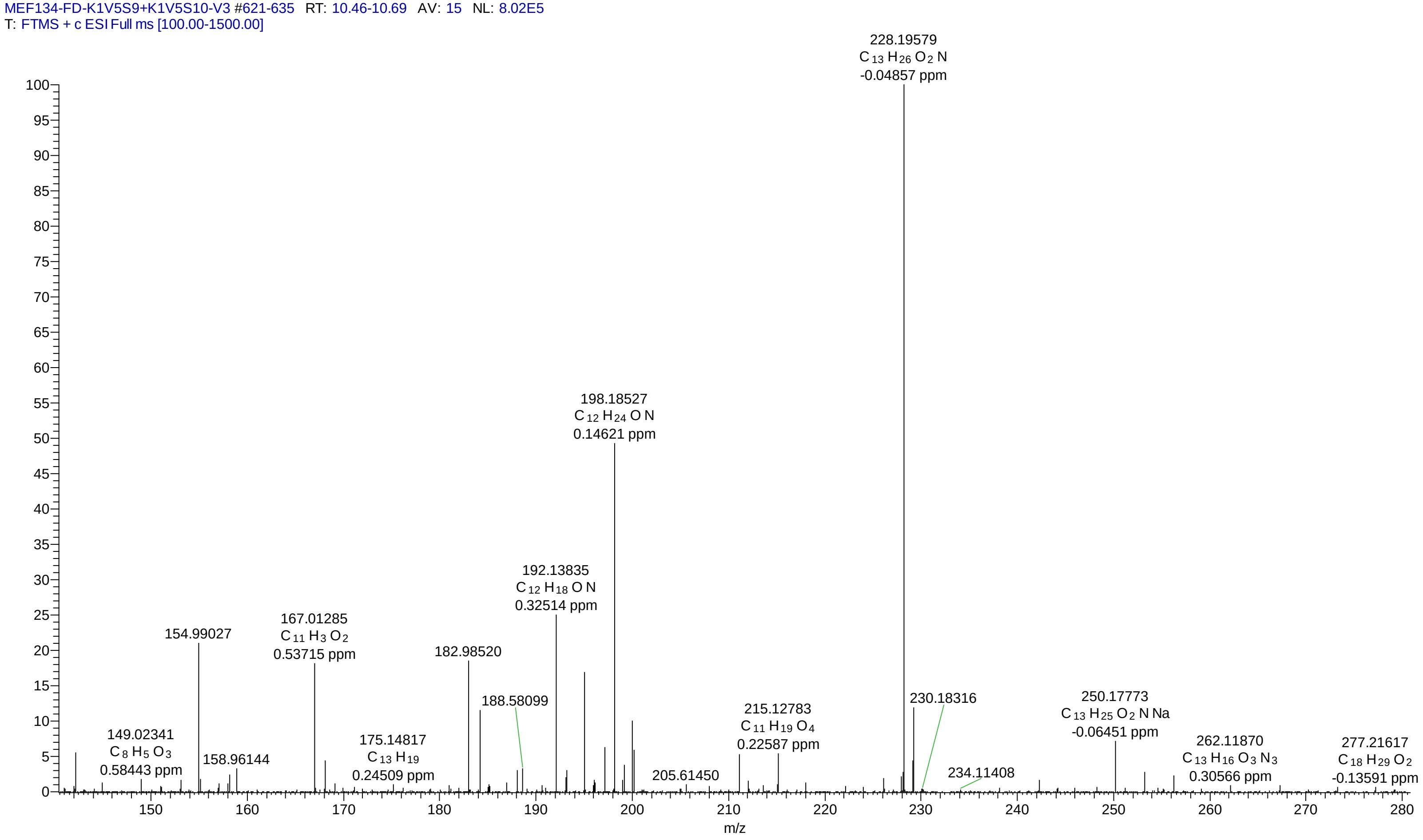

7. MEF134 FD K1V5S9+K1V5S10-V3 HPLC-HRMS Spectra

4

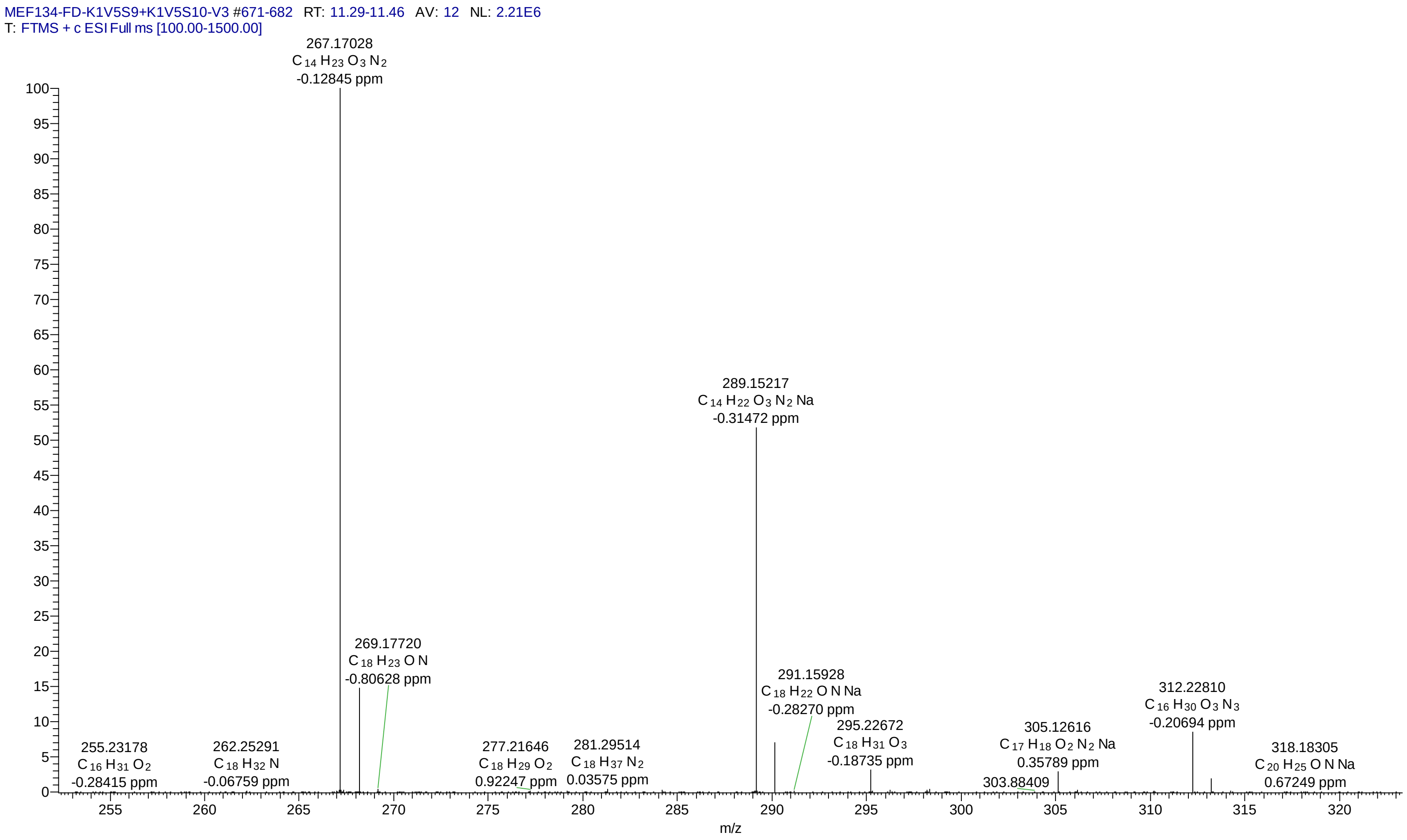

7. MEF134 FD K1V5S9+K1V5S10-V3

Impurity HPLC-HRMS Spectra

5

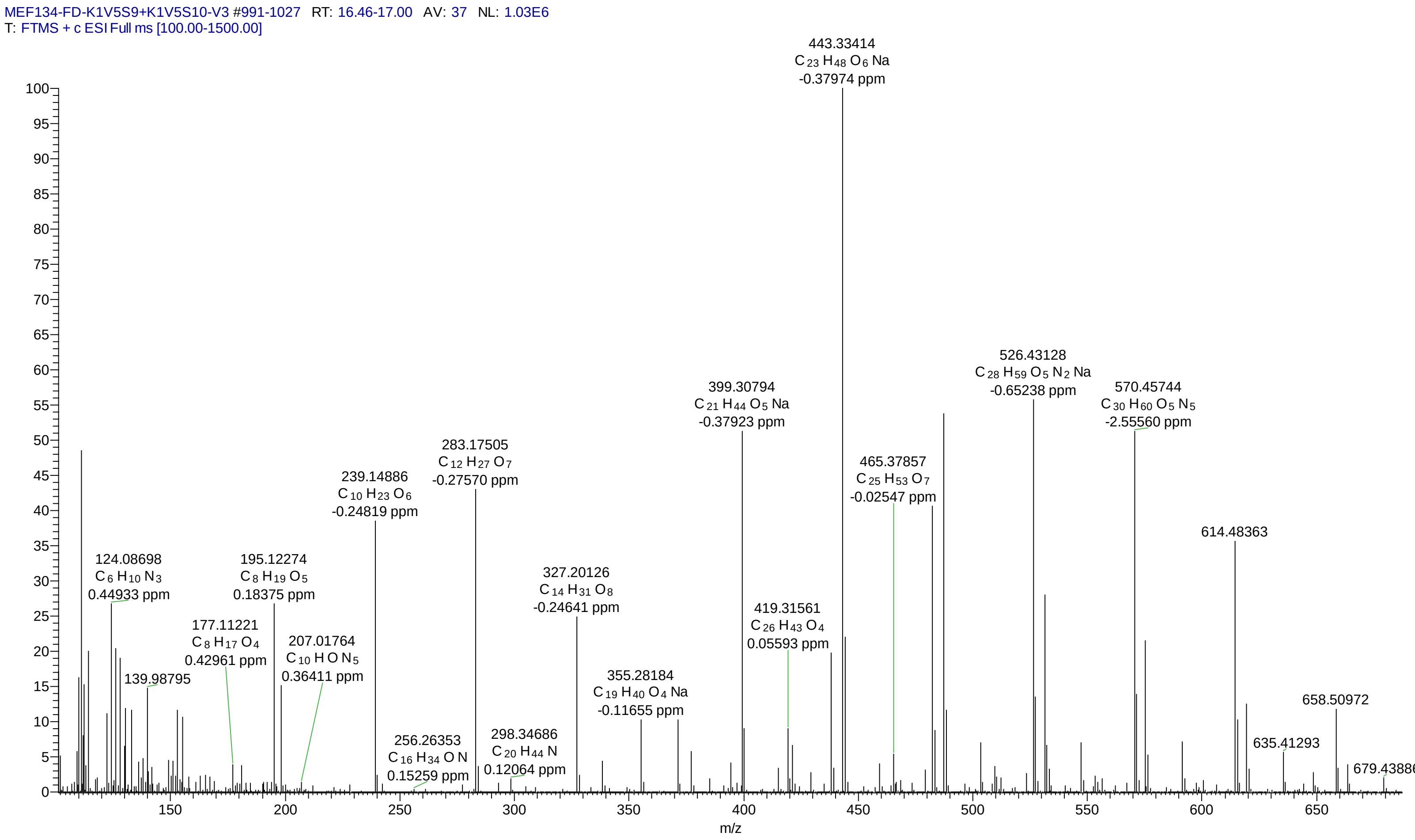

8. MEF134 FD K2V3S1-V5 HPLC-HRMS Fullscan

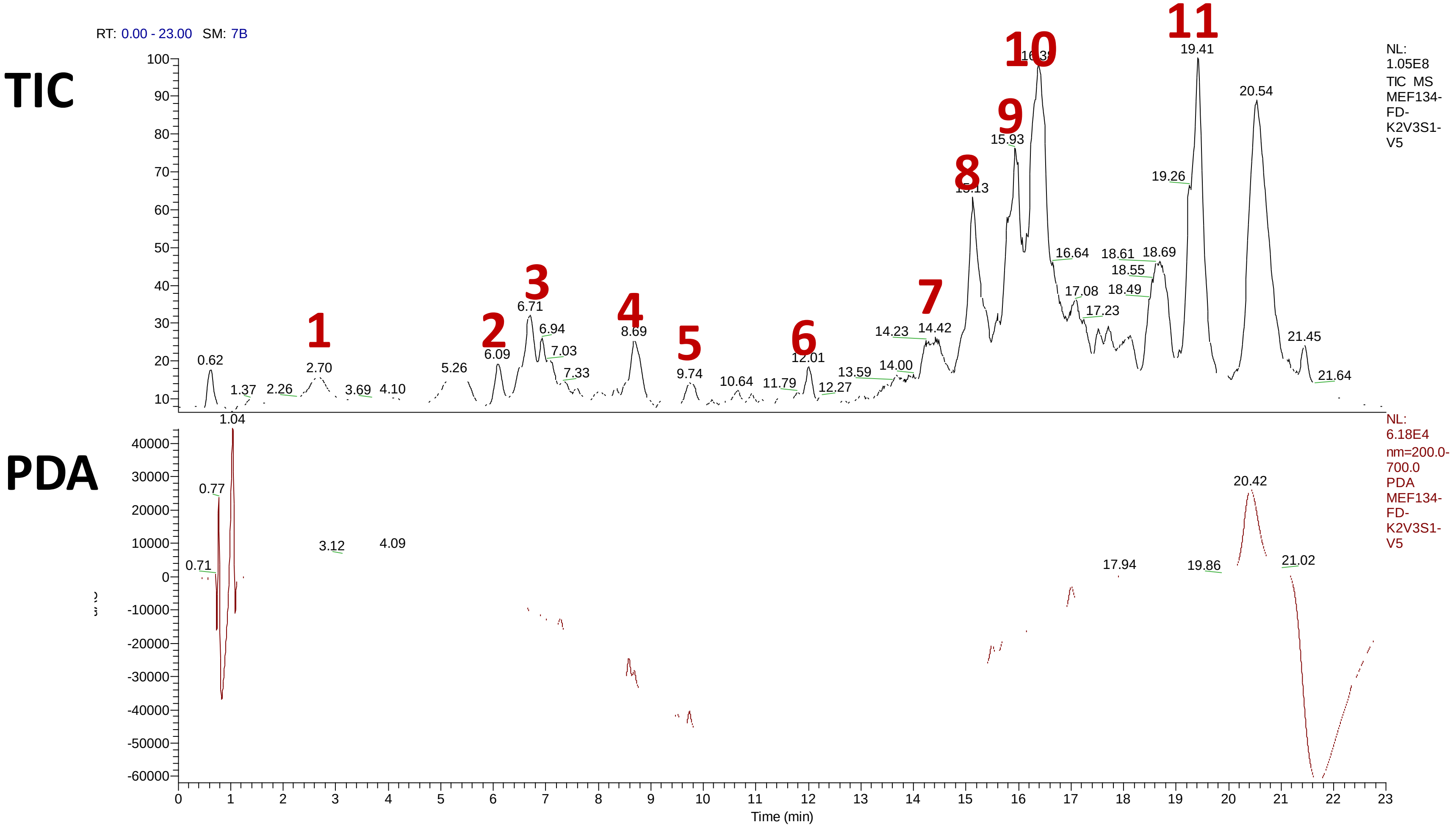

8. MEF134 FD K2V3S1-V5 HPLC-HRMS Spectra

1

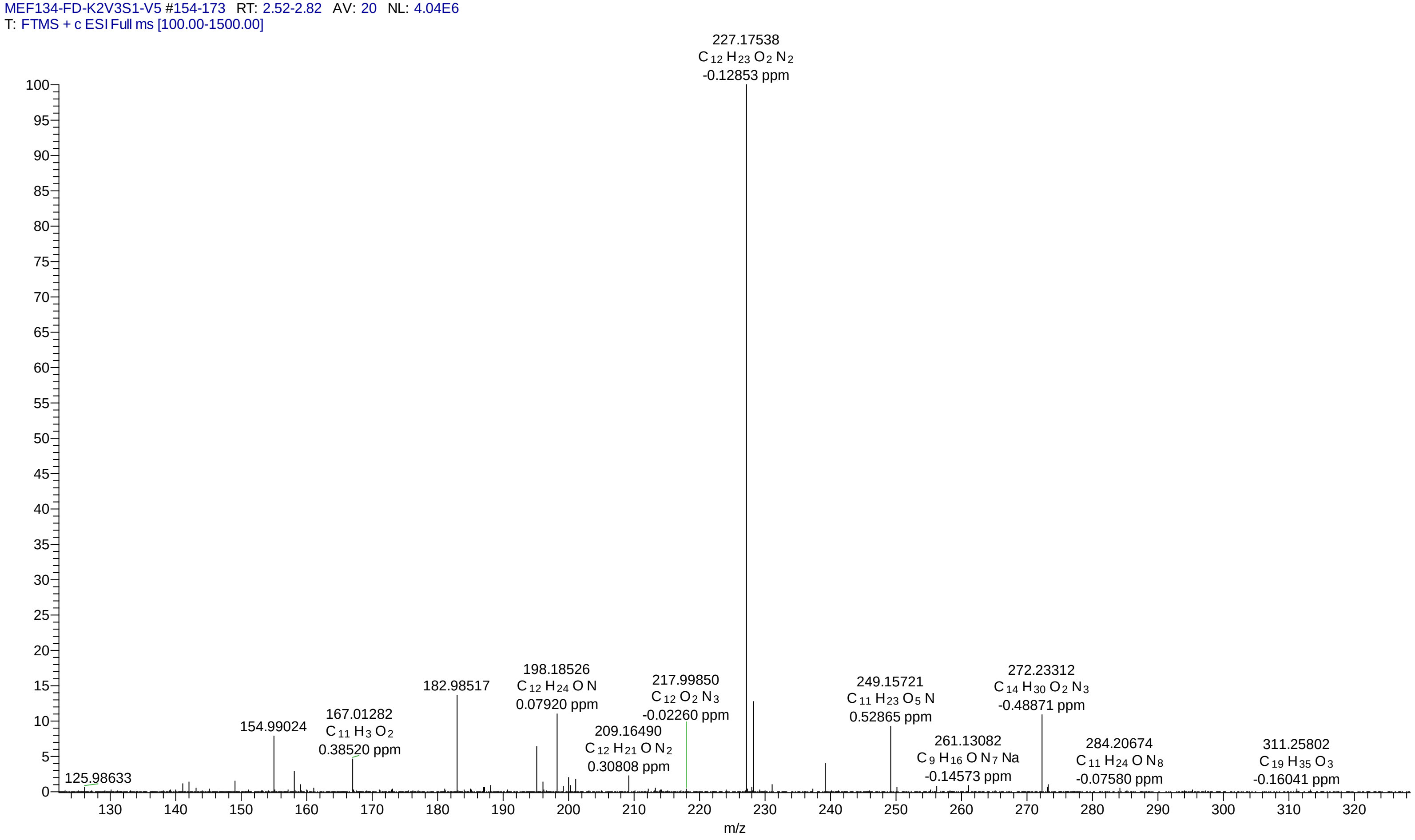

8. MEF134 FD K2V3S1-V5 HPLC-HRMS Spectra

2

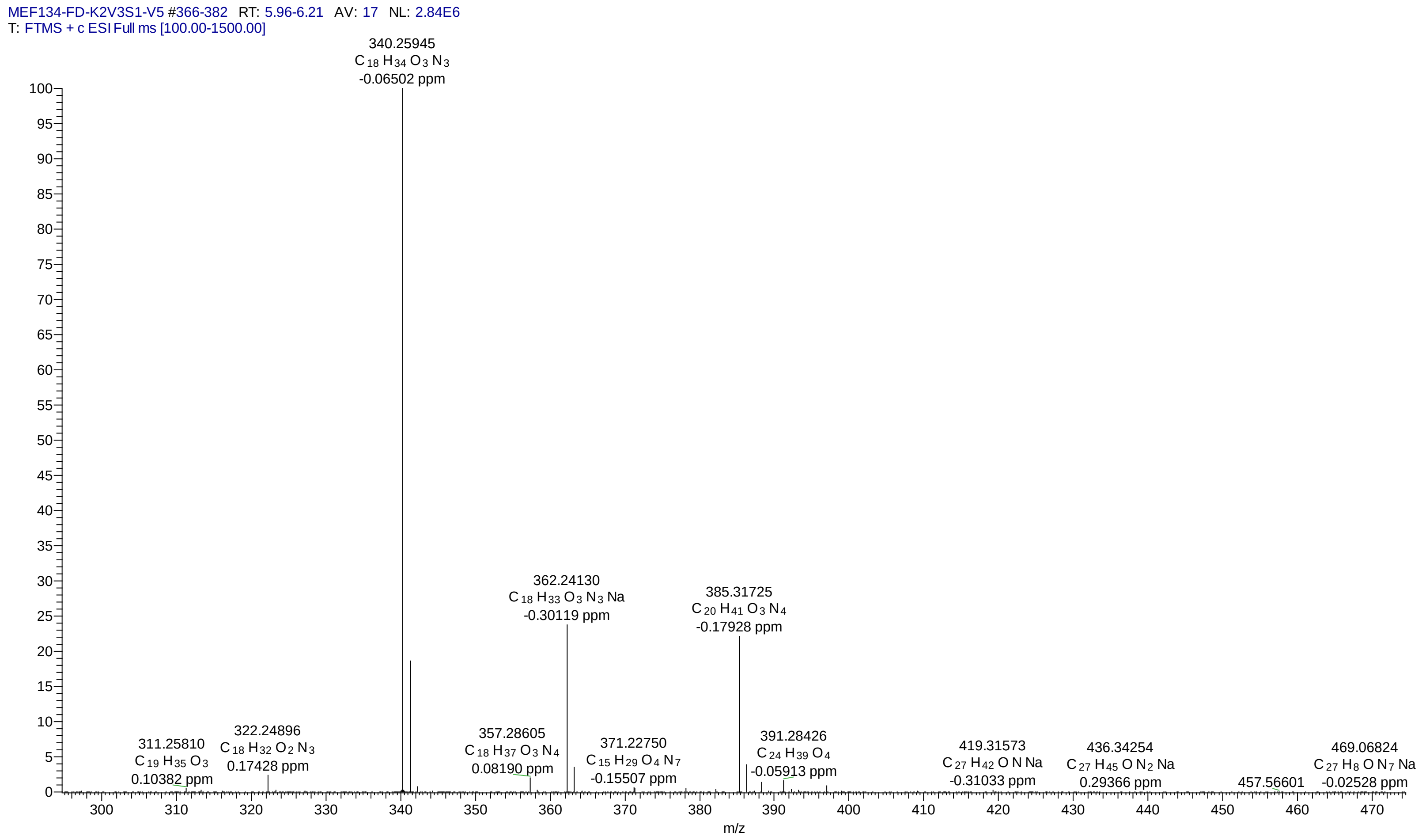

8. MEF134 FD K2V3S1-V5 HPLC-HRMS Spectra

3

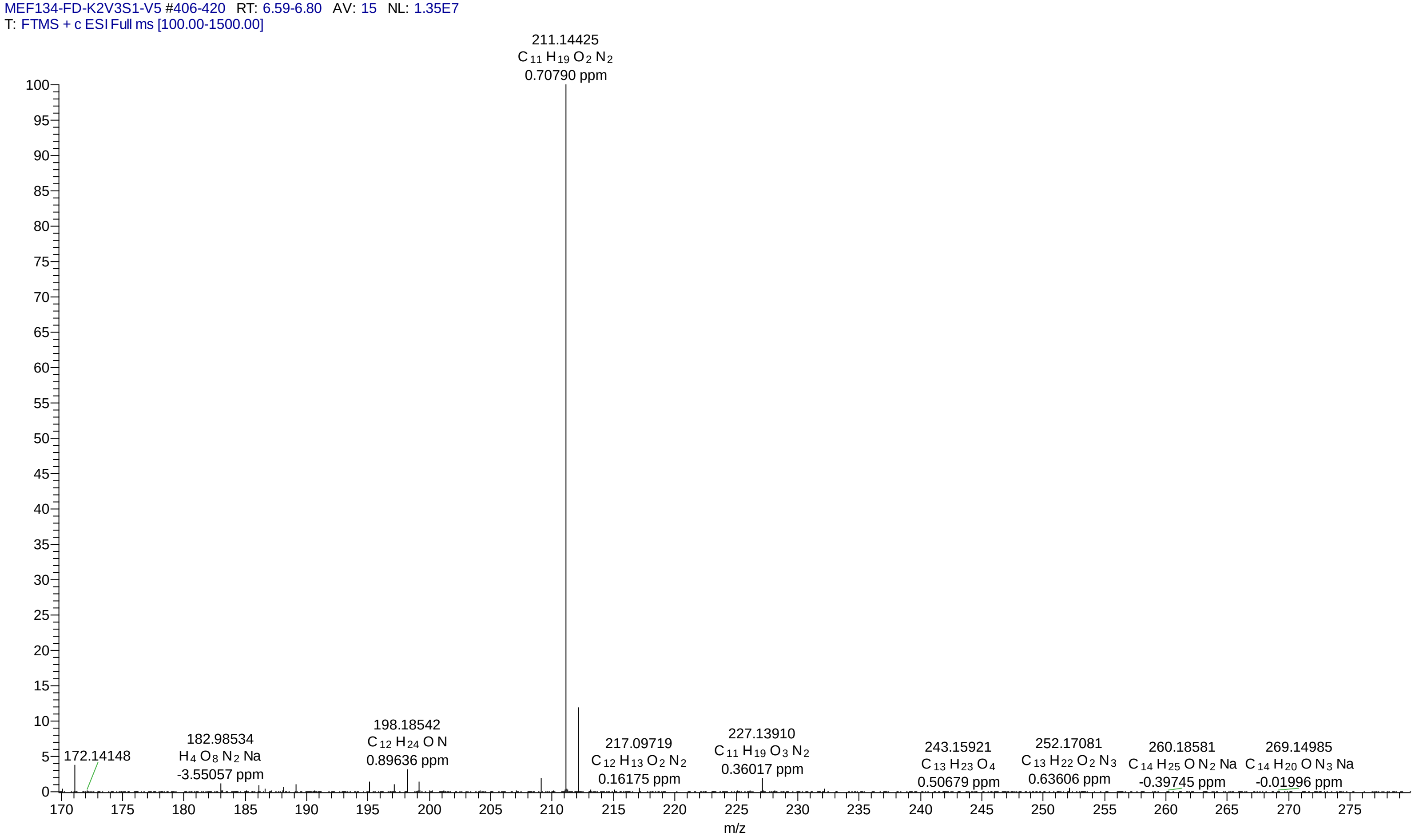

8. MEF134 FD K2V3S1-V5 HPLC-HRMS Spectra

4

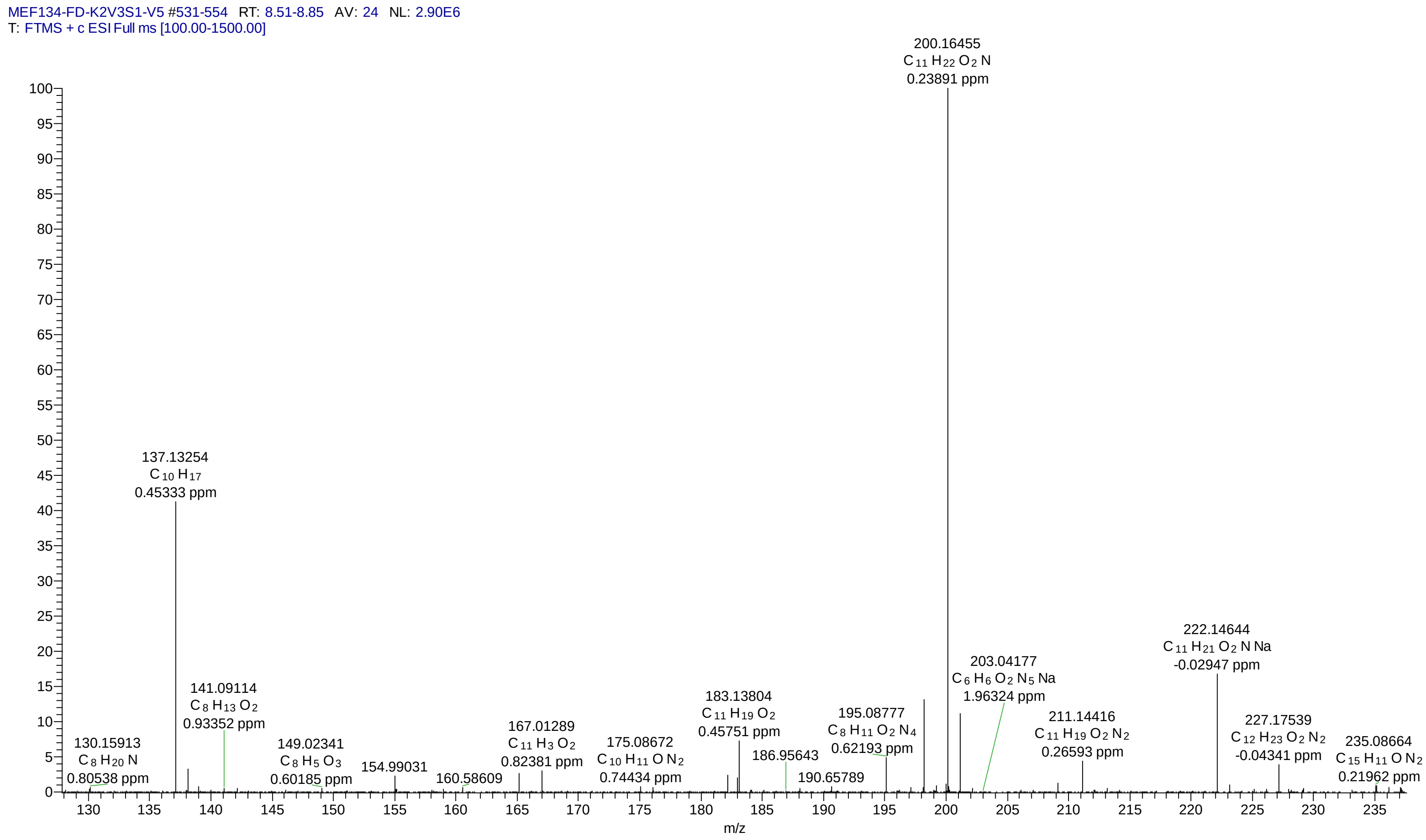

8. MEF134 FD K2V3S1-V5 HPLC-HRMS Spectra

5

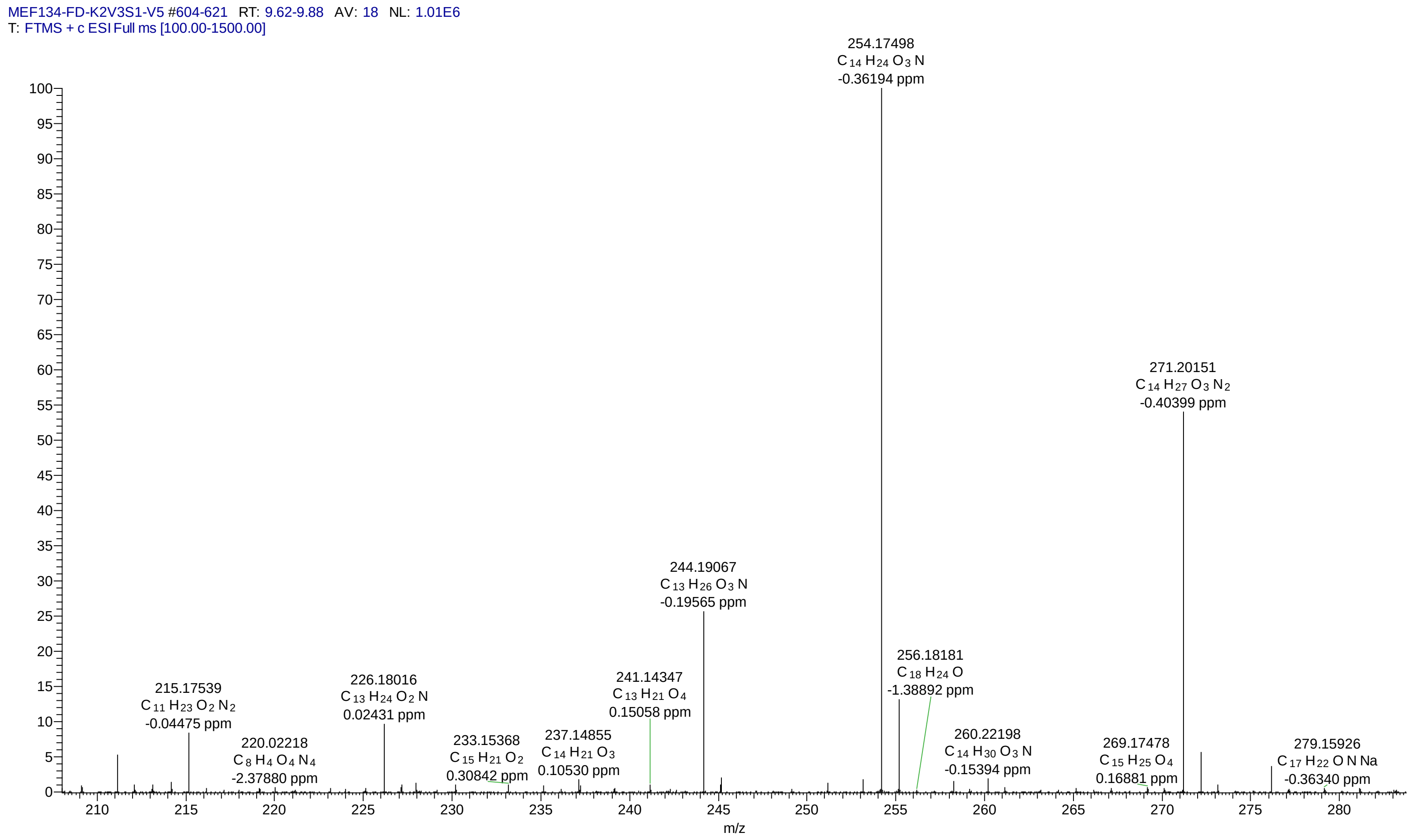

8. MEF134 FD K2V3S1-V5 HPLC-HRMS Spectra

6

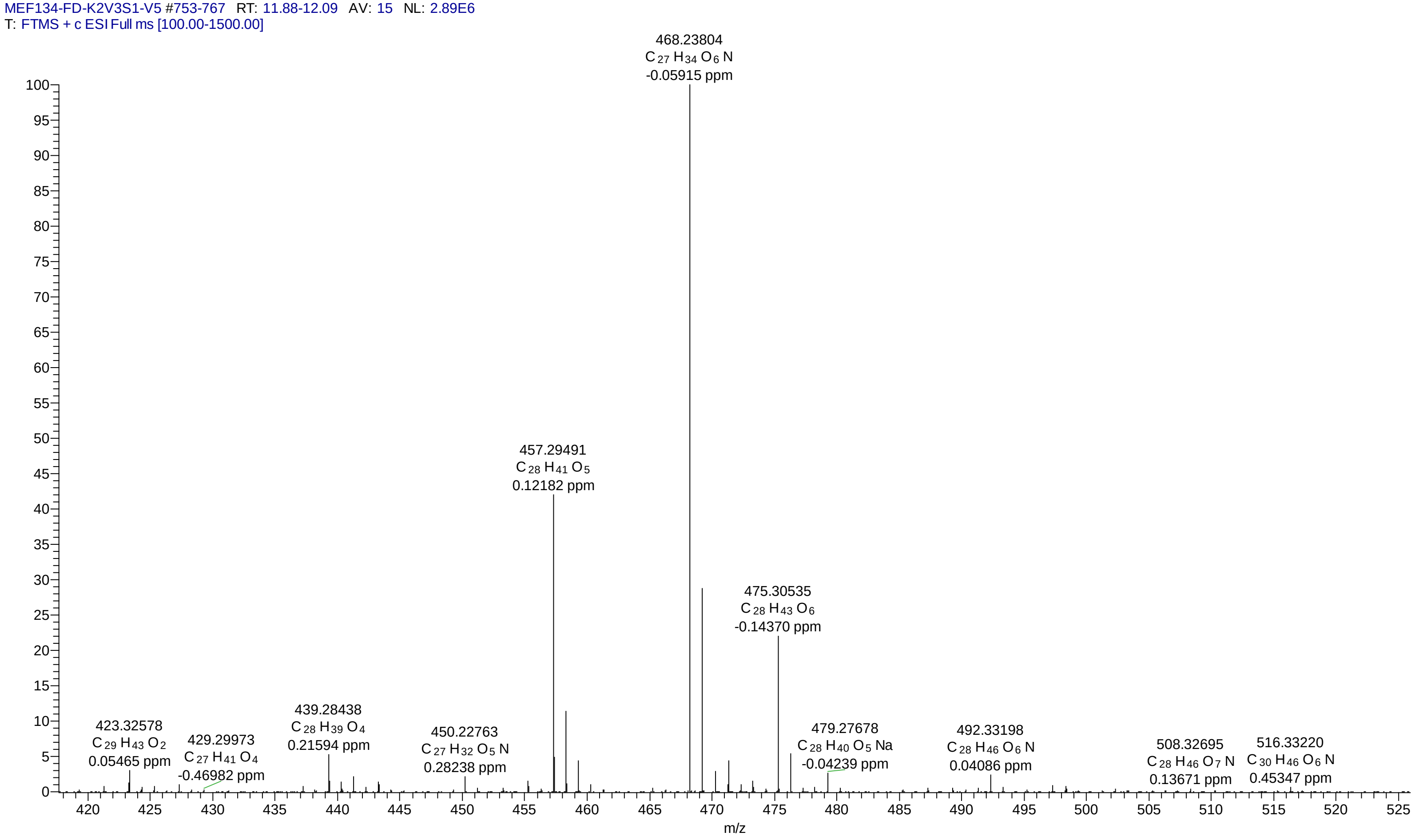

8. MEF134 FD K2V3S1-V5 HPLC-HRMS Spectra

Impurity

7

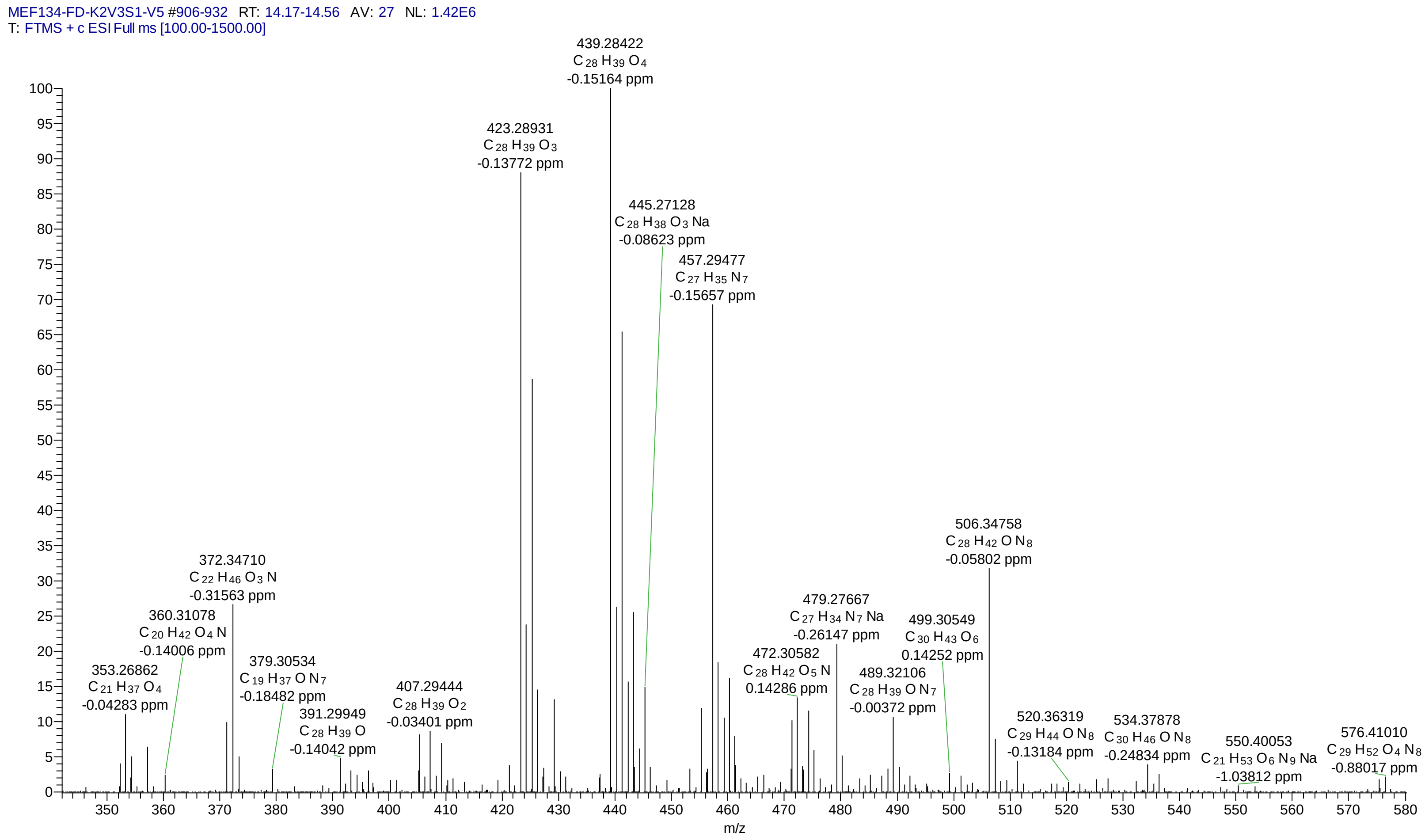

8. MEF134 FD K2V3S1-V5 HPLC-HRMS Spectra

8

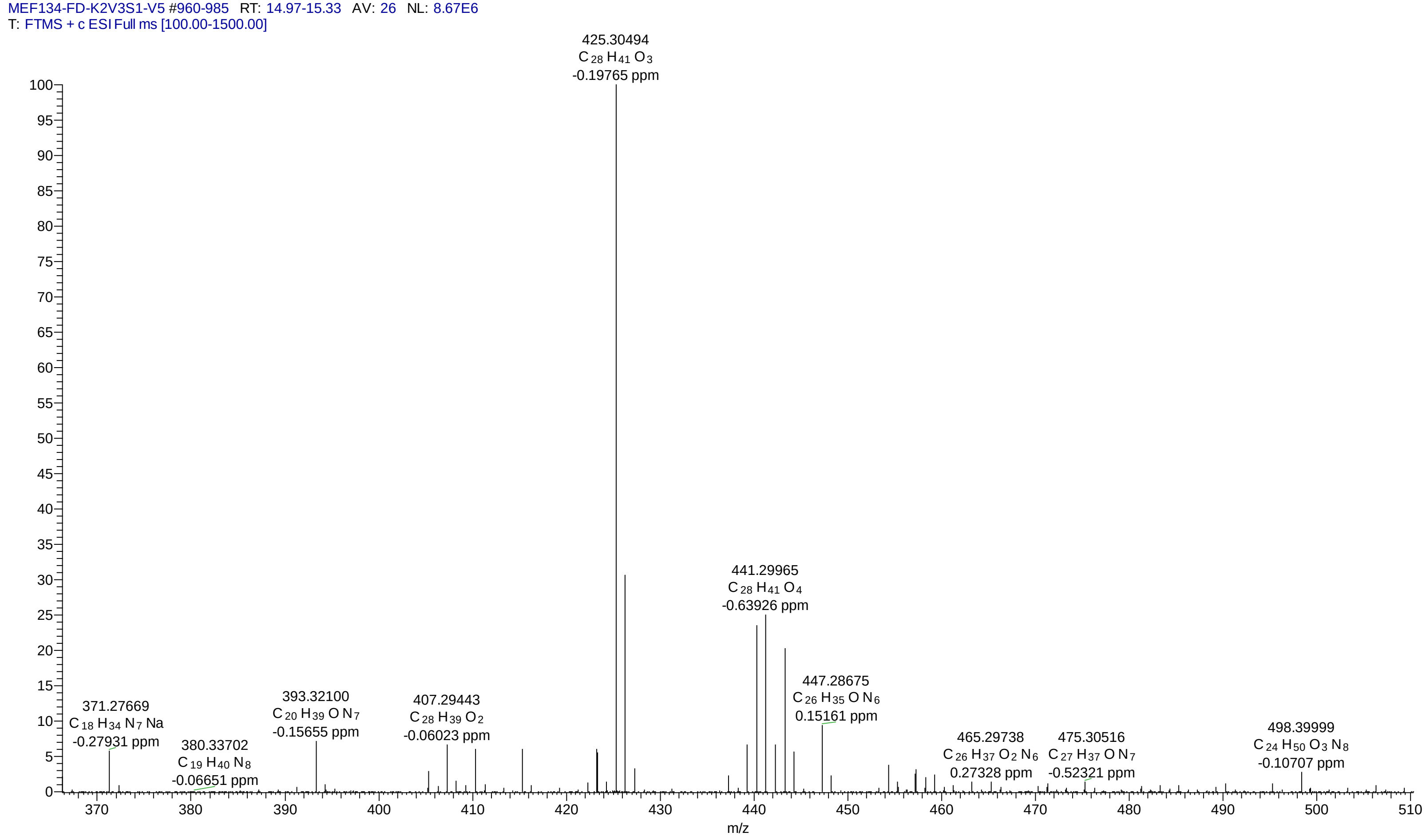

8. MEF134 FD K2V3S1-V5 HPLC-HRMS Spectra

9

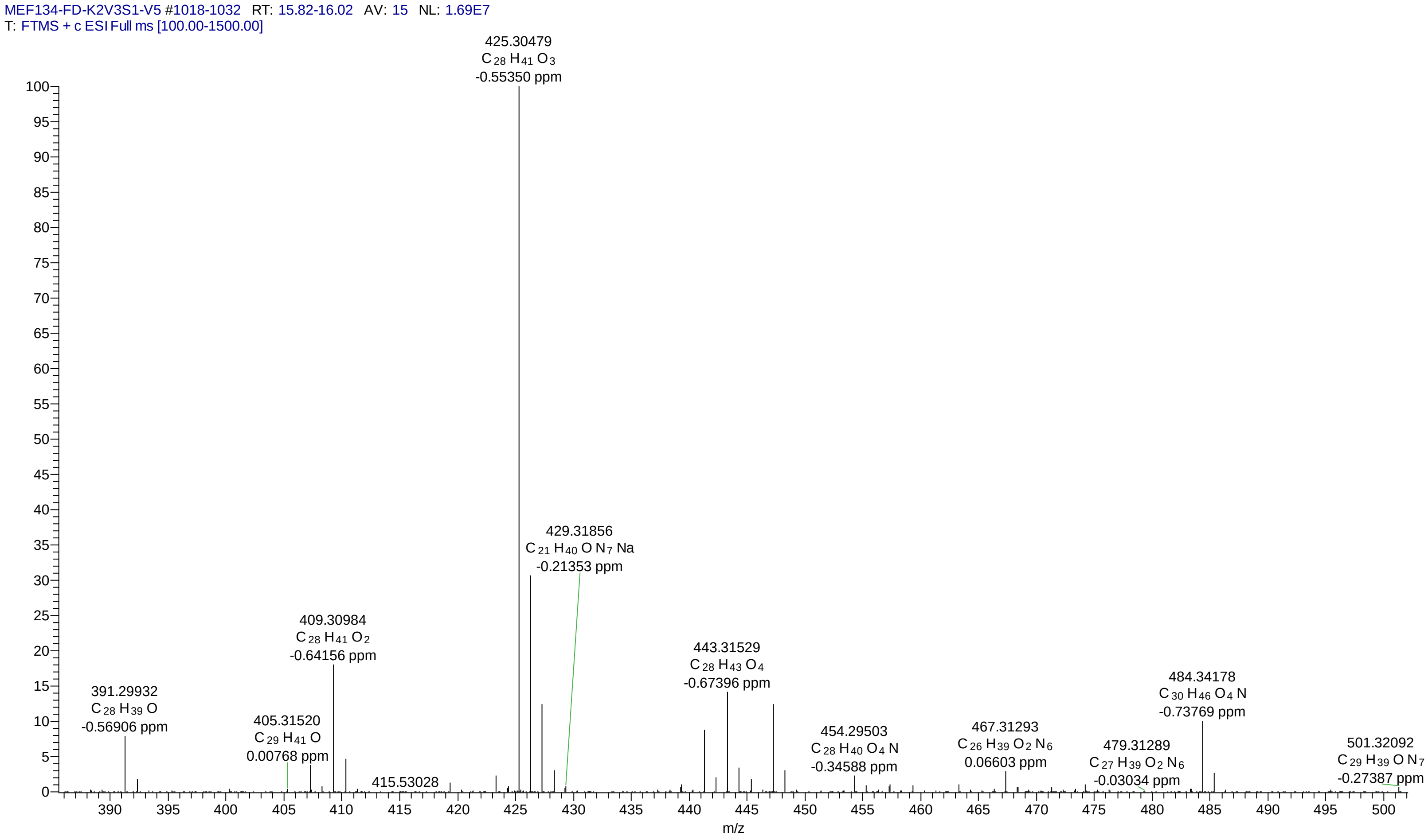

8. MEF134 FD K2V3S1-V5 HPLC-HRMS Spectra

10

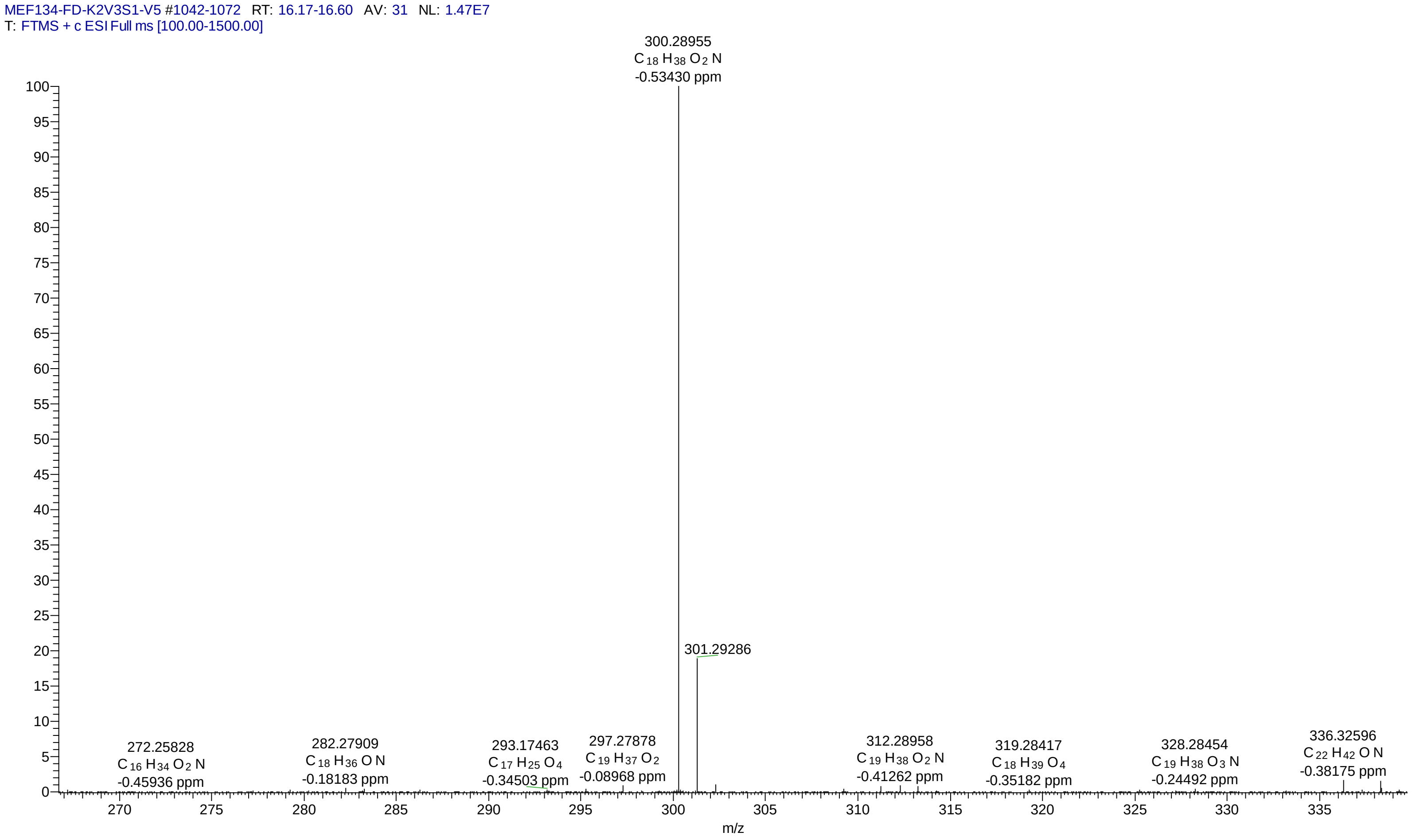

8. MEF134 FD K2V3S1-V5 HPLC-HRMS Spectra

11

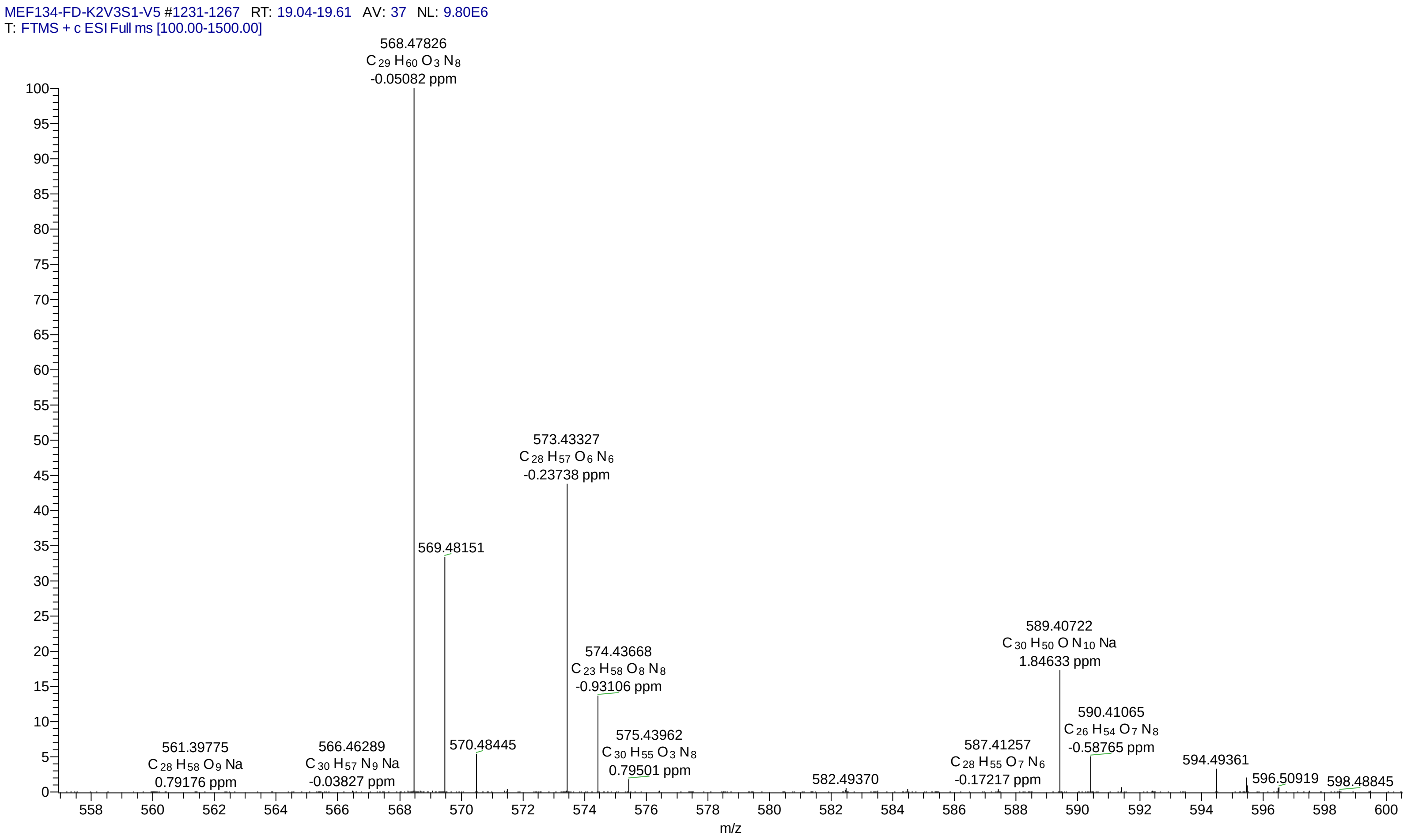

Supplement: Supplementary file 1 — Additional file 1:. MEF 134 Sample Analysis by Mass Spectrometry. [file 12864_2020_6684_MOESM1_ESM.pdf]
