# Supplementary figures and images for: Transcriptomic analysis of marine endophytic fungi extract identifies highly enriched anti-fungal fractions targeting cancer pathways in HepG2 cell lines
Source: BMC Genomics. 2020 Mar 30;21:265. doi: 10.1186/s12864-020-6684-z (PMC7106652; doi:10.1186/s12864-020-6684-z)

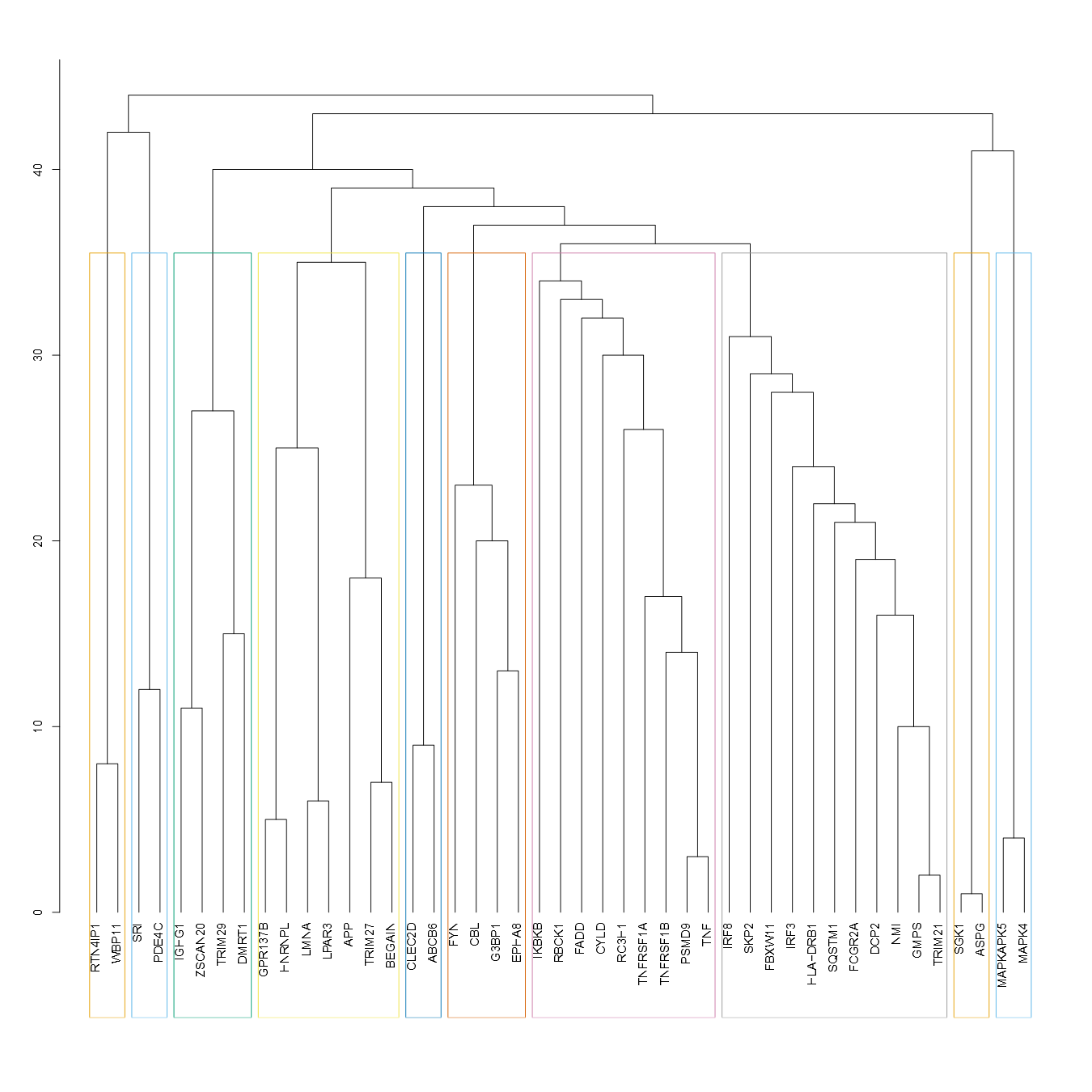

Supplement: Supplementary file 9 — Additional file 9: Figure S7. Dendrogram of community clustering of protein interaction networks of HepG2 cells treated with V1, V3 and V5. [file 12864_2020_6684_MOESM9_ESM.tif]
